# Supplementary figures and images for: The ROCEEH Out of Africa Database (ROAD): A large-scale research database serves as an indispensable tool for human evolutionary studies
Source: PLoS One. 2023 Aug 1;18(8):e0289513. doi: 10.1371/journal.pone.0289513 (PMC10393170; doi:10.1371/journal.pone.0289513)

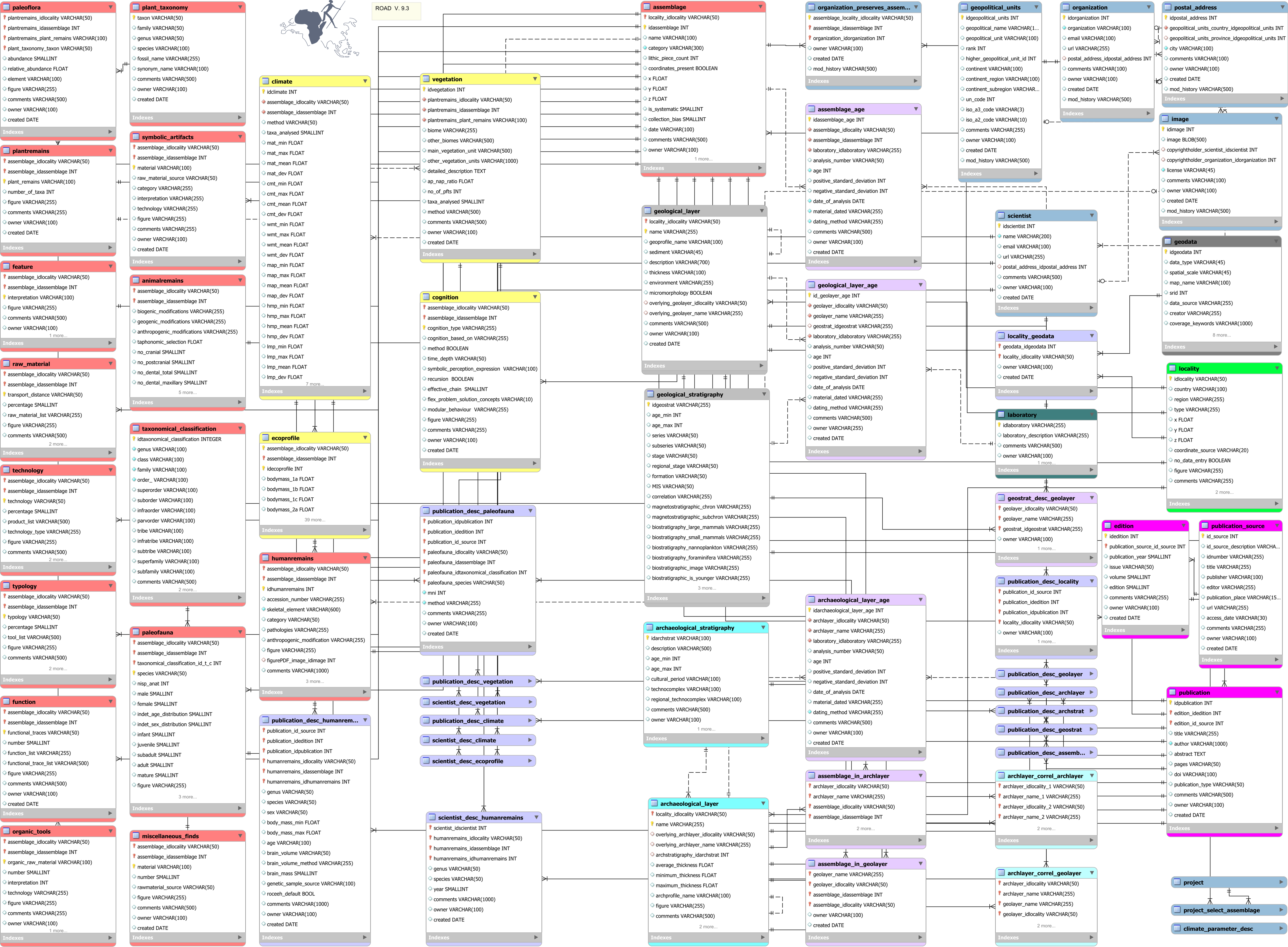

Supplement: S1 Fig — We developed this data model using the visual tool MySQL Workbench to create this Entity Relationship Diagram. The model depicts all tables and attributes in ROAD and shows primary and foreign keys with their relationships within the database. (TIF) [file pone.0289513.s001.tif]
