# Supplementary material for: The ROCEEH Out of Africa Database (ROAD): A large-scale research database serves as an indispensable tool for human evolutionary studies
Source: PLoS One. 2023 Aug 1;18(8):e0289513. doi: 10.1371/journal.pone.0289513 (PMC10393170; doi:10.1371/journal.pone.0289513)
Supplement: S1 Table — This list of the tables used for data entry is divided according to the four scientific disciplines. Tables are listed in the order they would be completed while entering data. Note that archaeological finds always have an archaeological layer that contains the cultural designation, while the other categories do not. Colors are coded to the disciplines, although entries in black are shared by all disciplines. (DOCX) [file pone.0289513.s002.docx]

| **ROAD TABLE NAMES** | **ARCH-AEOLOGY** | **HUMAN REMAINS** | **FAUNAL REMAINS** | **PLANT REMAINS** |
| --- | --- | --- | --- | --- |
| locality | X | X | X | X |
| locality_name_synonym | X | X | X | X |
| publication_desc_locality | X | X | X | X |
| geological_layer | X | X | X | X |
| publication_desc_geolayer | X | X | X | X |
| laboratory | X | X | X | X |
| geological_stratigraphy | X | X | X | X |
| geological_layer_age | X | X | X | X |
| publication_desc_geostrat | X | X | X | X |
| geostrat_desc_geolayer | X | X | X | X |
| archaeological_layer | X |  |  |  |
| archlayer_correl_archlayer | X |  |  |  |
| archlayer_correl_geolayer | X |  |  |  |
| publication_desc_archlayer | X |  |  |  |
| archaeological_layer_age | X |  |  |  |
| archaeological_stratigraphy | X |  |  |  |
| publication_desc_archstrat | X |  |  |  |
| assemblage | X | X | X | X |
| publication_desc_assemblage | X | X | X | X |
| assemblage_age | X | X | X | X |
| assemblage_in_geolayer | X | X | X | X |
| assemblage_in_archlayer | X |  |  |  |
| raw_material | X |  |  |  |
| typology | X |  |  |  |
| technology | X |  |  |  |
| function | X |  |  |  |
| organic_tools | X |  |  |  |
| symbolic_artifacts | X |  |  |  |
| feature | X |  |  |  |
| miscellaneous_finds | X |  |  |  |
| humanremains |  | X |  |  |
| publication_desc_humanremains |  | X |  |  |
| postal_address |  | X |  |  |
| organization |  | X |  |  |
| organization_preserves_assemblage |  | X |  |  |
| image |  | X |  |  |

| **ROAD TABLE NAMES** | **ARCH-AEOLOGY** | **HUMAN REMAINS** | **FAUNAL REMAINS** | **PLANT REMAINS** |
| --- | --- | --- | --- | --- |
| taxonomical_classification |  |  | X |  |
| paleofauna |  |  | X |  |
| publication_desc_paleofauna |  |  | X |  |
| animalremains |  |  | X |  |
| plantremains |  |  |  | X |
| plant_taxonomy |  |  |  | X |
| paleoflora |  |  |  | X |
| vegetation |  |  |  | X |
| publication_desc_vegetation |  |  |  | X |
| scientist_desc_vegetation |  |  |  | X |
| climate |  |  |  | X |
| publication_desc_climate |  |  |  | X |
| scientist_desc_climate |  |  |  | X |
