# Supplementary material for: The ROCEEH Out of Africa Database (ROAD): A large-scale research database serves as an indispensable tool for human evolutionary studies
Source: PLoS One. 2023 Aug 1;18(8):e0289513. doi: 10.1371/journal.pone.0289513 (PMC10393170; doi:10.1371/journal.pone.0289513)
Supplement: S1 File — This guide provides detailed definitions of all tables and their attributes, specifications for data entry and examples of possible entries. (PDF) [file pone.0289513.s003.pdf]

Heidelberger Akademie der Wissenschaften
FORSCHUNGSSTELLE “The Role of Culture in Early Expansions of Humans”

Heidelberg Academy of Sciences and Humanities
ROCEEH Research Center

Table Descriptions for the ROCEEH Out of Africa Database (ROAD):

A User’s Manual

ROCEEH
11.05.2022

TABLE OF CONTENTS

Introduction 5
Frequently Asked Questions (FAQs) 6
ROAD structure 7
Glossary of basic terms used in ROAD 9
General notes and tips for entering data 11
Database table descriptions 13
Locality 14
Table: locality 14
Table: locality_name_synonym 17
Table: geopolitical_units 18
Table: country_continent 19
Geological layers & dating 20
Table: geological_layer 20
Table: laboratory 22
Table: geological_layer_age 23
Table: geological_stratigraphy 26
Table: geostrat_desc_geolayer (geological stratigraphy describes geological layer) 31
Archaeological layers & dating 32
Table: archaeological_layer 32
Table: archaeological_layer_age 34
Table: archaeological_stratigraphy 37
Table: archlayer_correl_geolayer (archaeological layer correlates with geological layer) 39
Table: archlayer_correl_archlayer (archaeological layer correlates with archaeological layer) 40
Assemblage & dating 41
Table: assemblage 41
Table: assemblage_age 45
Table: assemblage_in_geolayer (assemblage in geological layer) 48
Table: assemblage_in_archlayer (assemblage in archaeological layer) 49
Archaeology 50
Table: raw_material 52
Table: typology 54

Table: technology 56
Table: function 58
Table: organic_tools 60
Table: symbolic_artifacts 62
Table: miscellaneous_finds 64
Table: feature 66
Cognition 68
Table: cognition 68
Paleoanthropology 72
Table: humanremains 72
Table: scientist_desc_humanremains (scientist describes human remains) 74
Botany 76
Table: plantremains 76
Table: paleoflora 78
Table: plant_taxonomy 80
Climate & vegetation 82
Table: climate 82
Table: scientist_desc_climate (scientist describes climate) 87
Table: vegetation 88
Table: scientist_desc_vegetation (scientist describes vegetation) 91
Ecoprofiles 92
Table: ecoprofile 92
Table: scientist_desc_ecoprofile (scientist describes ecoprofile) 96
Fauna 98
Table: taxonomical_classification 98
Table: paleofauna 100
Table: animalremains 103
Postal address, people & organizations 107
Table: postal_address 107
Table: scientist 108
Table: organization 109
Table: organization_preserves_assemblage 110
Geographical information 111
Table: geodata (geographic data) 111
Table: locality_geodata (locality has geographic data) 114

Source data ... 115
Table: publication_source ... 115
Table: edition ... 117
Table: publication ... 118
Publication data ... 120
Table: publication_desc_locality (publication describes locality) ... 120
Table: publication_desc_assemblage (publication describes assemblage) ... 121
Table: publication_desc_geolayer (publication describes geological layer) ... 122
Table: publication_desc_geostrat (publication describes geological stratigraphy) ... 123
Table: publication_desc_archlayer (publication describes archaeological layer) ... 124
Table: publication_desc_archstrat (publication describes archaeological stratigraphy) ... 125
Table: publication_desc_humanremains (publication describes human remains) ... 126
Table: publication_desc_climate (publication describes climate) ... 129
Table: publication_desc_vegetation (publication describes vegetation) ... 130
Table: publication_desc_paleofauna (publication describes paleofauna) ... 131
Table: image ... 133
Project tables ... 135
Table: project ... 135
Table: project_select_assemblage ... 136
ROAD table attribute descriptions ... 137
Table: road_table_attr_desc ... 137
References ... 138

Introduction

In 2008 the Heidelberg Academy of Sciences and Humanities inaugurated a new research center called “The Role of Culture in Early Expansions of Humans”, or ROCEEH for short. The research is projected to continue until the year 2027. The long-term objective of ROCEEH is to answer the question of when, where and in which form the interplay of changing climatic conditions, biological evolution and cultural development allowed the genus *Homo* to move beyond the behavioral niche of a large African ape and develop culturally defined niches outside of Africa. These niches are notably characterized by a wide spectrum of material and cognitive innovations. The goal of the research center is to identify the spatial and temporal patterns of migration in Africa and Eurasia and to reconstruct possible causes of early human expansions between three million years ago and the last glacial maximum about 20,000 years ago. Through the investigation of early migrations in Africa and the subsequent settlement of subtropical, warm temperate, and finally cold temperate and polar regions of Eurasia, the project will follow the transformation of the human species from its biologically determined nature into a culturally driven organism. The history of human expansions opens up new perspectives on the temporal and spatial dimensions of the increasing independence of the genus *Homo* from its purely biological limitations.

Frequently Asked Questions (FAQs)

What is the geographical range of ROCEEH?

ROCEEH covers the continents of Africa, Asia and Europe.

What is the chronological range of ROCEEH?

ROCEEH spans the period from 3,000,000 to 20,000 years before present.

When did the ROCEEH Out-of-Africa Database (ROAD) start?

The design and development of ROAD commenced in 2008. The current state of the database represents a progressive and continuous labor from its initial developmental phase, through its implementation, maintenance and upgrading. Data entry began in 2009 and is projected to continue until 2027.

What is the purpose of the ROAD database?

ROAD was developed as a tool to serve the ROCEEH research center in assimilating and analyzing large amounts of data from diverse scientific disciplines that include archaeology, paleoanthropology, paleontology, paleobotany, paleoclimatology and paleogeography. The database can be expanded and adapted to suit approaches from other scientific disciplines. For example, possible future links could include datasets from paleogenetics or paleotectonics, or data from younger periods.

What does ROAD do?

ROAD connects datasets about sites and assemblages with chronological and geographical data. Additionally, ROAD enables large scale analyses of derived datasets. This enhanced functionality enables ROAD to perform chrono-spatial analyses based on relevant information. As a result, ROAD generates distribution maps of various find categories and allows the depiction of multiple aspects of finds from different time ranges.

ROAD structure

The logical structure of ROAD is divided into six main themes. Each theme includes tables that constitute the basic building blocks of ROAD. In this manual, tables are designated by underlining. The tables can be further divided into individual elements or attributes. In this manual, **attributes** are designated by bold typeface. A detailed description of each table and its attributes follows in this manual. The six main themes include:

1) *Localities and assemblages* – Includes spatial information about localities (sites) and details about the assemblages (collections of finds) they contain. The assemblages may stem from archaeological, paleoanthropological, paleontological and paleobotanical investigations:
    - General tables: locality, assemblage

    ROAD also contains a vast array of geographic data (geodata), stored in vector and raster format, which are provided as map layers and related information (metadata). Geodata contain basic information, such as point type locations, boundaries, rivers and lakes, and include data about soils, geology, biomes, temperature, precipitation, ice coverage and many other physiographic, climatic or cultural aspects:
    - Geographic tables: geodata, locality_geodata

2) *Layers, profiles and dating information* – Combines stratigraphic information and age determinations of geological layers and profiles, archaeological layers and profiles, and assemblages of different types of finds. This theme considers both absolute (radiometric) and relative dating methods to estimate the minimum and maximum age of each layer:
    - Geological dating tables: geological layer, geological_layer_age, geological_stratigraphy, laboratory
    - Archaeological dating tables: archaeological_layer, archaeological_layer_age, archaeological_stratigraphy, laboratory
    - Assemblage dating tables: assemblage_age, assemblage_in_geolayer, assemblage_in_archlayer, laboratory

3) *Hominin behavior* – Comprises archaeological data about stone artifacts; tools made of organic materials such as bone, ivory or shell; symbolic artifacts such as art, musical instruments or ornaments; miscellaneous finds including ochre; and non-portable finds known as features. Information about cognition can also be added:
    - Archaeological tables: raw_material, typology, technology, function, organic tools, symbolic_artifacts, miscellaneous_finds, feature
    - Cognitive tables: cognition

ROAD structure (cont’d)

4) *Human remains* – Records data on hominin fossils, anatomy, sex, and age of the individual. Varying taxonomies are collected in separate tables, a method which allows us to associate more than one taxonomic interpretation to hominin specimens and/or a group of human fossils:
    - Anthropological tables: humanremains

5) *Environment and ecology* – Compiles data about the fauna and flora associated with cultural remains and hominin finds. Further information can be derived from these data, for instance, climate, vegetation, biomes and ecology:
    - Faunal tables: paleofauna, animalremains
    - Botanical tables: paleoflora, plant_taxonomy, plantremains, vegetation, scientist_desc_vegetation
    - Ecological tables: ecoprofile, scientist_desc_ecoprofile, climate, scientist_desc_climate

6) *Data sources* – Cites bibliographic information linking publications to localities, layers, ages, assemblages and nalyses. Other references can be added, for example, information about scientists, researchers, excavators and institutions. Analytical tables serve as tools to synthesize datasets. Descriptive tables can serve as metadata explaining the purpose of each table and attribute, or provide further information about countries:
    - Bibliographic tables: publication_source, edition, publication
    - Publication tables: publication_desc_locality, publication_desc_geolayer, publication_desc_geostrat, publication_desc_archlayer, publication_desc_archstrat, publication_desc_assemblage, publication_desc_humanremains, publication_desc_paleofauna, publication_desc_climate, publication_desc_vegetation, publication_desc_cognition
    - Refernce tables: scientist, excavator, person, organization, excavation_year
    - Analytical tables: project, project_select_assemblage
    - Descriptive tables: road_table_attr_desc, country_continent

Glossary of basic terms used in ROAD

ROCEEH – The Role of Culture in Early Expansions of Humans is a 20-year research center founded by the Heidelberg Academy of Sciences and Humanities in 2008.

ROAD – The ROCEEH Out-of-Africa Database PostgreSQL software and the University of Minnesota Mapserver to integrate multiple types of scientific information with geographic data.

Table – A table (e.g. locality, geological layer, assemblage) is the basic building block of ROAD where data about a specific topic are entered into the database. Each table contains attributes that allow further information about finds to be entered into the database. In this manual, table names are denoted by underlining.

Attribute – An attribute (e.g. **locality name**, **profile name**, **assemblage name**) is the dataset into which data about a specific characteristic are entered. In this manual, **attribute names** are denoted by bold type.

Primary key – A primary key is a unique identifier that may not be duplicated; it is a required attribute. A primary key can consist of one or more attributes. Primary keys are presented in yellow in the table descriptions.

Foreign key – A foreign key connects a dataset in a specific table "X" to another dataset in the table "Y". In the specific table "X" it may be duplicated, while in table "Y" it is unique. In general, a foreign key is not a mandatory attribute. Foreign keys are presented in pink in the table descriptions.

Primary key/Foreign key – A primary key/foreign key is a unique identifier in its specific table "X", and at the same time is also a foreign key. This means that the primary key/foreign key connects a dataset in a specific table "X" to another dataset in the table "Y". It is a required attribute. A primary key/foreign key is also presented in yellow in the table descriptions.

Different types of datasets:

- *String* – Data field may contain any kind of character, whether alphabet, number or punctuation.
- *Integer* – A whole number without decimal places (can be negative or zero).
- *Float* – Any number with decimal places (can be negative or zero).
- *Date* – The date format is entered as dd.mm.yyyy (d=day; m=month; y=year)
- *Boolean* – yes/no; true/false

Glossary of basic terms used in ROAD (cont’d)

Paired attribute – A paired attribute consists of two primary or foreign keys that, when considered together, comprise a unique identifier for a dataset.

Threefold attribute – A threefold attribute consists of three primary or foreign keys that, when considered together, comprise a unique identifier for a dataset.

Fourfold attribute – A fourfold attribute consists of four primary or foreign keys that, when considered together, comprise a unique identifier for a dataset.

Primary table – A primary table is a table into which basic datasets (primary data) are entered into the database (e.g. locality, assemblage, humanremains).

Linking table – A linking table joins two tables together (e.g. geostrat_desc_geolayer, archlayer_correl_geolayer, assemblage_in_archlayer, publication_desc_vegetation). In most cases, no new information is entered into a linking table, rather already existing datasets are joined together.

Hybrid tables –In some cases, primary data may be entered into a linking table (e.g. publication_desc_humanremains, publication_desc_paleofauna).

Look-up table – A look-up table contains fixed values that are used to complete attributes in other tables (e.g. archeological_stratigraphy, taxonomical_classification, paleoflora, laboratory. When required, a new value can be added to a look-up table.

Analytical table – An analytical table contains new datasets which are derived from primary data entered into ROAD (e.g. climate, ecoprofile, cognition). Analytical tables present new data which have been derived from the basic data.

General notes and tips for entering data

- English is the main language for all data entry in ROAD.
- Localities (sites) should follow the naming and spelling conventions as they appear in the literature including accents (e.g. Châtelperron, Kůlna Cave, Schöningen, Solutré).
- Do not use apostrophes (' ') or quotation marks (" ") in primary keys (e.g., locality, layer name, etc.) Such marks have a technical meaning for the database application and disturb its normal function.
- In cases where a locality has several names or is spelled in different ways, use the name most commonly cited in the literature.
- The table locality_name_synonym contains localities with:
    - multiple names (e.g. Saldanha – Elandsfontein – Hopefield, Broken Hill – Kabwe)
    - alternate transcriptions and spelling variations (e.g. Ain Boucherit – Ein Boucherit, El Kowm – El Khowm – El Qowm, Olduvai – Oldupai)
    - foreign accents (add a synonym using the base letter, e.g. enter e instead of é, o instead of ö, a instead of å, s instead of ş, etc.)
- The names of layers and assemblages should follow the naming and spelling conventions used by the authors, including their use of capital and lower case letters.
- If the name of a layer or assemblage includes a non-Latin letter or punctuation mark, you should write it out: e.g. alpha for α, prime for ´, hypen for -, bracket for ( ) or [ ], slash for /, etc.
- Use capital letters for locality names (e.g. Klasies River Mouth, Hohle Fels) and related acronyms (e.g. Olduvai Gorge FLK North site) as well as for abbreviated layer names (e.g. DAMA, RBS2).
- Avoid capital letters for other classes of data.
- Use lower case letters wherever possible, even when not customary or grammatically correct (e.g. levallois, handaxe, end scraper, oldowan, acheulean, table mountain formation, homo heidelbergensis, australopithecus, betula, quercus, taurotragus, cape zebra, etc.)
- Do not use punctuation marks with the exception of commas and periods.
- Do not use spaces before the first word or after the last word.
- Do not use more than one space between words.
- Do not use hard returns.
- Avoid abbreviations, even if commonly used.

General notes and tips for entering data (cont'd)

- Do not use commas, points or spaces in numbers to separate thousands:

  ✓ 1000000

  ☒ 1,000,000
  ☒ 1.000.000
  ☒ 1 000 000

- Use a decimal point, not a comma, for decimal values:

  ✓ 31.56

  ☒ 31,56

- Enter data hierarchically from largest to smallest entity (e.g. Klasies River Mouth Cave 1A layer SAS).
- Series of data should be entered using a comma followed by a single space to separate values. (e.g. order, family, genus, species).
- When value lists (similar to drop-down menus) are used for data entry, do not use commas. Commas are automatically generated when entries are selected from the menu.
- Enter data as singular, not plural, even when the data entered are not syntactically or grammatically correct:

  ✓ end scraper

  ☒ end scrapers

- If a number must be specified, enter it after the qualified entity:

  ✓ end scraper 15

  ☒ 15 end scrapers

Database table descriptions

The remainder of this document presents a detailed description of each of the tables and attributes used in ROAD. The information presented here is also available online in ROAD and can be viewed by clicking on the question mark symbols next to table names and attributes.

Each table description begins with an overview of a table's significance. Next, a tabularized list of attributes provides an overview of information about the type of data format (string, integer, float, date, Boolean), the key (primary in yellow, foreign in pink) and possible values for entering data. Finally, every attribute is described in detail, with further information added about data formats and rules. The goal of these table descriptions is to maximize the user's understanding and minimize inconsistencies in data entry.

Locality

Table: locality

The primary table locality provides basic information about each place where an assemblage of archaeological, paleoanthropological, paleontological, paleobotanical or other relevant materials was described, recorded, sampled or collected. Every locality (site) is situated in a specific country within a given geographic region. The name of every locality must be unique: If not, the name may consist of two parts including the general name followed by an underscore and the locality name (e.g. Geelbek_Alice). The locality type describes whether it is a cave, rockshelter, open air site, profile, mine, quarry or boring. Every locality must have a unique set of geographic coordinates measured in decimal degrees. If known, an elevation measured in meters above sea level can be entered. At a given locality there must be at least one geological profile. The profile may be idealized and its name should entered here.

**Primary table: locality**			
Attribute	Type	Key	Possible values
idlocality	String	Primary key	Geelbek_Alice
country	String		South Africa
region	String		Western Cape
type	String		open air
x	Float		14.57944
y	Float		-33.21083
z	Float		39.86
coordinate_source	String		unconfirmed, checked, landmark, gps
figures	String		[automatically generated]
no_data_entry	Boolean		true, false
summary	String		brief site description
comments	String		general comments
owner	String		Alicia Keys
created	Date		[automatic date stamp]
mod_history	String		[automatic record of changes]

The attribute **idlocality** specifies the unique name of a locality (e.g. Sibudu Cave, Die Kelders, Olduvai Gorge FLK site). For places with multiple localities, the name can take the format "Place_Locality" (e.g. Geelbek_Stella). Follow precisely the naming conventions used by the authors, including their use of capital and lower case letters, as well as foreign accents. However, single apostrophes (') and quotation marks (") are not allowed in ROAD. In cases where multiple names have been assigned to a locality, use the most common name for the locality and add an alias in the table locality_name_synonym. In cases whether names are spelled differently, use the name that seems most common in the literature and add an alias in the table locality_name_synonym. When entering a new locality, select "new option" from the bottom of the list.

The attribute **country** specifies the name of the country where a locality is situated (e.g. China, Germany, Kenya, Saudi Arabia) and is selected from a fixed drop-down menu.

The attribute **region** specifies the name of a geographical region (e.g. province, state, district, department) in the country where a locality is situated (e.g. Baden-Württemberg, Western Cape) and is selected from a fixed drop-down menu. If the region is not known, select “unknown” from the top of the list.

The attribute **type** specifies the type of locality (e.g. cave, rockshelter, open air, profile, outcrop, mine, quarry, boring). Select the type of locality from the existing list.

The attribute **x** is the geographic longitude of the locality in decimal degrees. East longitude is entered as a positive number, west longitude as a negative number. To convert degrees, minutes and seconds into decimal degrees, use the following formula:

$$\frac{\left(\frac{Seconds}{60} + Minutes\right)}{60} + Degrees = Decimal\ Degrees$$

e.g. E 14° 34’ 46”

$$\frac{\left(\frac{46}{60} + 34\right)}{60} + 14 = 14.57944$$

The attribute **y** is the geographic latitude of the locality in decimal degrees. North latitude is entered as a positive number, south latitude as a negative number. To convert degrees, minutes and seconds into decimal degrees, use the following formula:

$$\frac{\left(\frac{Seconds}{60} + Minutes\right)}{60} + Degrees = Decimal\ Degrees$$

e.g. S 33° 12.65’

$$\frac{\left(\frac{0}{60} + 12.65\right)}{60} + 33 = -33.21083$$

The attribute **z** is the elevation of a locality in meters above sea level, and should be entered if it is known. A negative elevation would indicate that a locality is below sea level.

The attribute **coordinate_source** contains information about the source of coordinates for a locality. This attribute helps determine the reliability and precision of the coordinates. The four fixed entries for coordinate source are selected from a drop-down menu:

1. unconfirmed: coordinates known from literature or other sources, but whose precision is unknown or has not yet been checked by ROCEEH
2. checked: coordinates that have been checked by ROCEEH through a map resource such as Google Earth and are considered to reliably indicate a locality's position
3. landmark: coordinates that have been corrected by ROCEEH with a geographic landmark and can now be considered as a very good indicator of a locality's position
4. gps: coordinates that have been directly measured in the field using a handheld GPS and are considered as a very good indicator of a locality's position.

The attribute **figures** contains an automatically generated path name for images which have been uploaded to the database. This attribute represents a link to a visualization of the locality and can be a sketch, drawing, or photo.

The attribute **no_data_entry** should be clicked for localities where only the table locality is completed, but no further information is available about stratigraphy, dating or assemblages. This attribute may be used to indicate well-known localities which were not entered into ROAD. It is used mainly in cases where sources of literature are unreliable or unavailable. The name of such a locality can still be entered in ROAD, but with no further data entry. For normal data entry, this box remains unticked and f (false) appears in the data table. However, if the option for no data entry is necessary, tick this box and t (true) will appear in the data table. This option can also be used for sites which were reviewed, but not entered into ROAD. Enter a note in the comments explaining why the site was excluded, e.g. old excavation, no dating, no stratigraphy, site too old, site too young, literature not available, etc.

The attribute **summary** presents an abbreviated text summarizing the important features of a site in 2-3 sentences. This brief text may present highlights such as special or unusual finds, first appearances of specific finds, time frames, cultural periods, or important facts about the excavation history. Try to be original and interesting!

The attribute **comments** contains general comments about the nature of the locality.

The attribute **owner** automatically indicates the last person who entered or changed the dataset.

The attribute **created** automatically indicates the date this dataset was originally entered. An additional attribute **mod_history** automatically records the modification history of a dataset including the names of users who made previous changes and the dates those changes were made.

Table: locality_name_synonym

This primary table joins the locality name from the table locality with its commonly used synonyms, spelling variations and abbreviations. If more than two synonyms are used, each synonym must be entered separately (e.g. Peers Cave – Fish Hoek – Skildergat Cave). Use the locality synonym to enter common spelling variations (e.g. Olduvai – Oldupai), different transliterations (e.g. Nahr al Kabir – Nar el Kebir), commonly used abbreviations (e.g. Duinefontein – DFT) and variants without foreign accents (e.g. Schöningen – Schoningen, Saint Côme de Fresné – Saint Come de Fresne) using the base letter.

**Primary table: locality_name_synonym**			
Attribute	Type	Key	Possible values
id_locality_name_synonym	String	Primary key	[automatically generated]
locality_name_1	String	Foreign key	[chosen from list]
locality_name_2	String		Saldanha, Hopefield
comments	String		general comments
owner	String		Nathaniel Hawthorne
created	Date		[automatic date stamp]

The attribute **id_locality_name_synonym** is a unique, automatically generated, consecutive number assigned to each dataset.

The attribute **locality_name_1** specifies the locality from the table locality (e.g. Kabwe). Select the locality from the list.

The attribute **locality_name_2** contains a synonym for the locality (e.g. Broken Hill). Enter the synonym name here.

The attribute **comments** contains general comments about the synonyms of the locality.

The attribute **owner** automatically indicates the last person who entered or changed the dataset.

The attribute **created** automatically indicates the date this dataset was originally entered. An additional attribute **mod_history** automatically records the modification history of a dataset including the names of users who made previous changes and the dates those changes were made.

Table: geopolitical_units

The look-up table geopolitical_units contains information about the administrative entities used in ROAD. These are hierarchically structured depending on the system used in a given country. For example the county Pima (rank 3) is located in the state Arizona (rank 2) which is located in the country United States of America (rank 1).

**Look-up table: geopolitical_units**			
Attribute	Type	Key	Possible values
idgeopolitical_units	Integer	Primary key	[automatically generated]
geopolitical_name	String		Tübingen, Baden-Württemberg, Germany
geopolitical_unit	String		city, country, area, region, district
rank	String		1, 2, 3, 4
higher_geopolitical_unit_id	Integer	Foreign key	[chosen from list]
owner	String		Jonathan Stroud
created	Date		[automatic date stamp]

The attribute **idgeopolitical_units** is a unique, automatically generated, consecutive number assigned to each dataset.

The attribute **geopolitical_name** provides the name of the administrative entity (e.g. Tübingen, Baden-Württemberg, Germany).

The attribute **geopolitical_unit** indicates the type of administrative entity (e.g. district, province, country). The units depend on the hierarchical structure used in a given country.

The attribute **rank** provides the hierarchical level used for ordering the administrative entity.

The attribute **higher_geopolitical_unit_id** specifies to which higher ordered administrative entity the geopolitical unit belongs (e.g. Trieste → Friuli-Venezia Giulia → Italy). These are specified as numbers.

The attribute **owner** automatically indicates the last person who entered or changed the dataset.

The attribute **created** automatically indicates the date this dataset was originally entered. An additional attribute **mod_history** automatically records the modification history of a dataset including the names of users who made previous changes and the dates those changes were made.

Table: country_continent

The look-up table country_continent contains a list of countries and the continent to which they belong, as determined by geography and not politics. For example, Egypt is located in Africa, while Israel is in Asia. We consider Armenia, Azerbaijan, Georgia and Cyprus as part of Asia, but Moldova and the Ukraine belong to Europe. We consider Russia, Turkey and Kazakhstan as part of Asia, although they straddle both Asia and Europe. For disputed territories, we enter the customary name of the territory without ascribing it to a specific country. In addition, we exclude several island countries from ROAD, e.g. Comoros, Iceland, Japan.

**Look-up table: country_continent**			
Attribute	Type	Key	Possible values
idcountry_continent	Integer	Primary key	[automatically generated]
country	String		Namibia, China, France
continent	String		Africa, Asia, Europe
region	String		East Africa, Southwest Asia, Northern Europe
comments	Date		general comments including sub-regions

The attribute **idcountry_continent** is a unique, automatically generated, consecutive number assigned to each dataset.

The attribute **country** provides the name of the country, as defined politically.

The attribute **continent** provides the name of the continent in which the country is situated. The scope of ROCEEH encompasses only Africa, Asia and Europe.

The attribute **region** provides the name of the region to which a country is attributed. Region is a subjective grouping made using information about the prehistory, geography and politics of a specific region (e.g. Central Africa, Southern Europe, East Asia). The region helps the user when writing queries by allowing certain countries in a region to be grouped together.

The attribute **comments** contains general comments about the country. Predefined sub-regions are listed here, (e.g. Nile Valley, Maghreb, Levant, Arabia, Scandinavia, Baltics).

The attribute **created** automatically indicates the date this dataset was originally entered. An additional attribute **mod_history** automatically records the modification history of a dataset including the names of users who made previous changes and the dates those changes were made.

Geological layers & dating

Table: geological_layer

The primary table geological_layer characterizes the lithology of a single geological layer within an idealized geological profile at a locality where an assemblage was documented. A geological layer can be defined based on its sediment type, description, thickness and depositional environment, as well as its stratigraphic relationship to the other geological layers in the same profile. A geological layer can be analyzed using micromorphological methods.

**Primary table: geological_layer**			
Attribute	Type	Key	Possible values
locality_idlocality	String	Primary key	Darbas-2
name	String	Primary key	L23, 3a, VII, SGS
geoprofile_name	String		Darbas-2a
sediment	String		silty diatomite
description	String		sandy body, yellow, cross-bedding, clayey pebbles, fine sand, silt and clay
thickness	String		0.75, 1-4
environment	String		lacustrine
micromorphology	Boolean		true, false
overlying_geolayer_idlocality	String	Foreign key	[chosen from list]
overlying_geolayer_name	String	Foreign key	[chosen from list]
comments	String		general comments
owner	String		Boris Becker
created	Date		[automatic date stamp]

The paired attributes **locality_idlocality** and **name** specify the locality that contains the geological layer and the name of that geological layer.

The attribute **geoprofile_name** designates the name of the geological profile. If possible, use one idealized profile that synthesizes the entire sedimentary sequence. When necessary, add additional profiles. Name the profiles according to the authors' designations (e.g. West), but if needed add the author's name and/or year of excavation (e.g. Wadley 1999 Upper). Do not use the word “profile” in the name!

The attribute **sediment** describes the predominant lithology of the geological layer (e.g. clay, sandy silt, fine sand, gravel, marl, silty diatomite, peat, clayey loam).

The attribute **description** describes the lithology and other geological features of the geological layer in more detail (e.g. yellowish brown (7.5YR 4/4) silty fine sand with carbonate concretions in the upper portion, poorly cemented).

The attribute **thickness** indicates the thickness of the geological layer in **meters**, if known. Use a period for the decimal point (not a comma). The data can be entered as a single number (e.g. 1.5) or as a range (e.g 1.2-2.75). Do not use spaces around the hyphen, and do not use symbols such as >, < or ~. Convert inches, feet and yards to meters as follows:

$$1\,inch\ =\ 2.54\,cm\ =\ 0.0254\,m$$
$$1\,foot\ =\ 12\,inches\ =\ 30.48\,cm\ =\ 0.348\,m$$
$$1\,yard = 3\,feet = 36\,inches = 91.44\,cm = 0.914\,m$$
$$1\,meter\ =\ 100\,cm = 3.28\,feet\ =\ 39.37\,inches$$

The attribute **environment** describes the environment in which the sediments were deposited (e.g. aeolian, lacustrine, fluviatile, marine, swamp, cave).

The attribute **micromorphology** indicates whether or not micromorphological studies have been conducted. True indicates “yes”; false indicates “no”.

The paired attributes **overlying_geolayer_idlocality** and **overlying_geolayer_name** indicate the locality and name of the geological layer lying directly above the given layer. In some cases, there may be a gap between two geological layers. If a gap is known and its thickness can be measured (e.g. 5 m inaccessible, 13 m not exposed), it should be entered as a “dummy” geological layer, defined and described accordingly. If the size of the gap is unknown and the horizontal distance between the profiles is great, the two profiles can be considered as separate profiles.

The attribute **comments** contains general comments about the nature of the geological layer.

The attribute **owner** automatically indicates the last person who entered or changed the dataset.

The attribute **created** automatically indicates the date this dataset was originally entered. An additional attribute **mod_history** automatically records the modification history of a dataset including the names of users who made previous changes and the dates those changes were made.

Table: laboratory

The look-up table laboratory provides information about analytical laboratories used to radiometrically date samples or conduct other types of chemical or physical analyses. Each laboratory has a unique identification (**idlaboratory**) and full name (**laboratory_description**). Make sure to follow naming conventions exactly with regard to upper and lower case letters.

**Look-up table: laboratory**			
Attribute	Type	Key	Possible values
idlaboratory	String	Primary key	Beta, KIA, GrA
laboratory_description	String		Beta Analytic, Miami, Florida, USA
owner	String		Chelsea Clinton
created	Date		[automatic date stamp]

The attribute **idlaboratory** specifies the official abbreviation of laboratories which conduct analyses, if known. Lists of laboratories conducting radiocarbon analysis are documented in the publication *Radiocarbon* and its website. Other types of laboratories have their own sets of abbreviations. Make sure to follow naming conventions exactly with regard to upper and lower case letters. Additional laboratories may be added to the list as needed, but **idlaboratory** may not be duplicated. The most recent list (2021) of current and retired radiocarbon laboratories can be downloaded at: http://radiocarbon.webhost.uits.arizona.edu/node/11.

The attribute **laboratory_description** specifies the full name of a laboratory used to date samples or conduct other types of chemical or physical analyses. Include details such as address, city, state and country for each new entry as well as contact details and website, if available. An asterisk (*) in the table indicates laboratories that are closed, no longer measuring 14C, or operating under a different code designation.

The attribute **owner** automatically indicates the last person who entered or changed the dataset.

The attribute **created** automatically indicates the date this dataset was originally entered. An additional attribute **mod_history** automatically records the modification history of a dataset including the names of users who made previous changes and the dates those changes were made.

Table: geological_layer_age

The primary table geological_layer_age specifies the age of a geological layer based on results reported by an analytical laboratory for a single sample from a specific geological layer. Geological dating results help determine the age of the geological layer. Dating results for an archaeological layer should be entered in the table archaeological_layer_age, while dating results for an assemblage should be entered in the table assemblage_age. Only enter a dating result once, regardless of the age table selected. Each dated sample has a unique identification number generated by the laboratory as reported in a publication. The analytical laboratory provides a report containing a result, stated as a number in years before present, and a positive and negative standard deviation which are not always the same. The dating method and the material dated should be specified.

**Primary table: geological_layer_age**			
Attribute	Type	Key	Possible values
id_geolayer_age	Integer	Primary key	[automatically generated]
geolayer_idlocality	String	Foreign key	[chosen from list]
geolayer_name	String	Foreign key	[chosen from list]
laboratory_idlaboratory	String	Foreign key	[chosen from list]
analysis_number	String		Pta-2345, GrA-1234
age	Integer		20000, 225000
positive_standard_deviation	Integer		5000
negative_standard_deviation	Integer		4500
date_of_analysis	Date		2007
dating_method	String		14C, OSL, U-series
material_dated	String		Sand
comments	String		general comments
owner	String		Dwight Eisenhower
created	Date		[automatic date stamp]

The attribute **id_geolayer_age** is a unique, automatically generated, consecutive number assigned to each dataset.

The paired attributes **geolayer_idlocality** and **geolayer_name** indicate the locality and name of the geological layer from the table geological_layer.

The attribute **laboratory_idlaboratory** is the official abbreviation for the designated analytical laboratory and should match the entry from the table laboratory. Make sure to follow naming conventions exactly with regard to upper and lower case letters. If the laboratory is not in the table laboratory, you should first enter it there.

The attribute **analysis_number** specifies the official laboratory number assigned to the dated sample (e.g. Pta-2345, GrA-1234, OxA-X-2456-45). Always include the lab prefix, followed by a single hyphen, and then the official number provided by the laboratory. Make sure to follow naming conventions exactly with regard to upper and lower case letters. Use hyphens as separators instead of spaces.

Please note that the **analysis number** is usually <u>not</u> the same as a sample number! A sample number is often related to a specific locality, profile, layer or square, the year the sample was taken, or the person who took the sample. For example, DRS-E-10 could designate sample number 10 taken from the east profile of Diepkloof Rockshelter, GK-2001-06 could indicate sample 6 taken at Geissenklösterle in 2001, or JK-1559 might signify sample 1559 taken by J. Krause. Sample numbers should rather be entered in the comments, unless no other identifying information for the sample is provided in the publication.

The attribute **age** specifies the result of the dating analysis as reported by the laboratory in years before present. If the result is 142 ka, then enter 142000. For radiocarbon dates, always enter <u>uncalibrated</u> radiocarbon ages. All other dates are entered as years before present (BP). Do not use decimals places or thousand separators such as commas, periods or spaces. Do not enter "+" or "-".

The attribute **positive_standard_deviation** specifies the positive standard deviation of the dating analysis in years. Do not use decimals places or thousand separators such as commas, periods or spaces. Do not enter "+" or "-". Enter a positive standard deviation even if it is the same as the negative standard deviation. If the date is a minimum (or infinite) age, meaning that the dating results are beyond the limits of the specific method, enter "1000000".

The attribute **negative_standard_deviation** specifies the negative standard deviation of the dating analysis in years. Do not use decimals places or thousand separators such as commas, periods or spaces. Do not enter "+" or "-". Enter a negative standard deviation even if it is the same as the positive standard deviation. If the date is a minimum (or infinite) age, meaning that the dating results are beyond the limits of the specific method, enter "0".

The attribute **date_of_analysis** specifies the year of the analysis/report or the first publication of the results in the format yyyy. If the year is unknown, enter "0".

The attribute **dating_method** specifies the method of analysis (e.g. 14C, AMS, TL, OSL, IRSL, ESR, U-Series, Ar/Ar, Amino Acid Racemization, etc.) More detailed information about the dating method can be entered under **comments**.

The attribute **material_dated** specifies the general type of material analyzed (e.g. bone, tooth, antler, ivory, charcoal, ostrich eggshell, shell marine, wood, plant remains, calcrete, lithic, unknown, etc.) More detailed information about the material dated can be entered under **comments**.

The attribute **comments** contains general comments about the dating results.

The attribute **owner** automatically indicates the last person who entered or changed the dataset.

The attribute **created** automatically indicates the date this dataset was originally entered. An additional attribute **mod_history** automatically records the modification history of a dataset including the names of users who made previous changes and the dates those changes were made.

Table: geological_stratigraphy

The primary table geological_stratigraphy characterizes the geological age of a stratigraphic unit, for a geological layer, an archaeological layer or an assemblage, in chronostratigraphic terms as defined by the international commission for stratigraphy. See http://www.stratigraphy.org or Cohen et al. (2013). The minimum and maximum ages of a layer can be more precisely defined by combing information about the dating of the geological and archaeological layers, the dating of assemblages contained in the layer, and other relevant information such as marine isotope stage, magnetostratigraphy, biostratigraphy or geological correlation with another dated layer. This table lists the methods used to confirm the chronostratigraphic age. Depending on their source, results from absolute dating should also be entered in one of the following tables:

1. geological_layer_age
2. archaeological_layer_age
3. assemblage_age.

**Primary table: geological_stratigraphy**			
Attribute	Type	Key	Possible values
idgeostrat	String	Primary key	Sibudu BMOD-OMOD2, Nachukui Kaitio Member, Middle Awash Aduma Ardu B
age_min	Integer		75000
age_max	Integer		100000
series	String		Pleistocene, Pleistocene/Holocene
subseries	String		Early Pleistocene, Middle/Late Pleistocene
stage	String		Calabrian, Gelasian, Ionian, Piacenzian, Zanclean
regional_stage	String		Aspheronian, Gurian, , Kuyalnikian Santernian
formation	String		Sisian Suite, Sabbie di Imola Formation
mis	String		31, 23-21, 10-7, 5e, 5b-a, 2
correlation	String		Archaeological stratigraphy, geology, large mammal biostratigraphy, magnetostratigraphy, oxygen isotope stratigraphy, radiocarbon (C14) dating, uranium series dating
magnetostratigraphic_chron	String		Matuyama
magnetostratigraphic_subchron	String		Brunhes(C1n), Matuyama(C1r)/Brunhes(C1n), Matuyama(C1r), Olduvai(C2n)/Matuyama(C1r)
biostratigraphy_large_mammals	String		Villafranchium

biostratigraphy_small_mammals	String		
biostratigraphy_nannoplankton	String		NN 15, NN 21
biostratigraphy_foraminifera	String		PL 5, PT 1b
biostratigraphic_image	String		[automatically generated]
comments	String		general comments
owner	String		Eustache Chapuys
created	Date		[automatic date stamp]

The attribute **idgeostrat** is the name of the stratigraphic unit described. In most cases, **idgeostrat** consists of **idlocality** followed by the **name** of the geological layer (e.g. Bushman Rock Shelter layer 2). However, in some cases, a geological profile can be a regional reference section and therefore used at more than one locality, for example at Olduvai Gorge, Koobi Fora, Sangiran, and others. If this is the case, do not enter the **idlocality** as part of the **idgeostrat**. Instead, enter the name of the geological reference profile followed by the name of the geological layer (e.g. Nachukui Formation Nariokotome Member). This reference profile can be used at more than one locality (e.g. the Nachukui Formation Nariokotome Member is found at the localities of Nariokotome, Kokiselei and Lokalalei.)

The attributes **age_min** and **age_max** indicate the age range of a stratigraphic unit in years before present. Do not use commas, periods or spaces. The attributes **age_min** and **age_max** are extremely important because they ascribe an age range to each geological layer, archaeological layer or assemblage. The age can be determined by dating the geological layer, by dating the archaeological layer, or by dating an assemblage. Additionally, an age can be determined by correlating layers geologically or archaeologically, by comparing archaeological typology and stratigraphy, or by combining any of these factors to help narrow the age range of a geological layer. Thus, **age_min** and **age_max** serve an analytical function and contain the quintessential information that delivers the narrowest possible age for a layer. When several dates are available from multiple sources, **age_min** and **age_max** enable multiple dating results to be summarized into a single, combined result. In cases where one intervening layer is undated, **age_min** of the immediately overlying layer and **age_max** of the immediately underlying layer can be used. In cases where two or more intervening layers are undated, **age_min** from the uppermost dated layers and **age_max** from the lowermost dated layers can be used for each of the intervening layers. If these attributes are not completed here, it will not be possible to use the geological stratigraphy of this layer or assemblage in further analyses.

The attribute **series** provides the name of the international chronostratigraphic series determined for the stratigraphic unit described (e.g. Pliocene, Pleistocene, Holocene). Use a slash if the entry straddles two series (e.g. Pliocene/Pleistocene, Pleistocene/Holocene). Choose carefully from the drop-down menu, and note that new entries are not allowed. See http://www.stratigraphy.org.

The attribute **subseries** indicates the full name of the international chronostratigraphic subseries (e.g. Early Pliocene, Middle Pliocene, Late Pliocene, Early Pleistocene, Middle Pleistocene, Late Pleistocene). Use a slash if the entry straddles two subseries (e.g.

Early/Middle Pliocene, Late Pliocene/Early Pleistocene, Middle/Late Pleistocene). Choose carefully from the drop-down menu, and note that new entries are not allowed. See http://www.stratigraphy.org.

The attribute **stage** indicates the name of the international chronostratigraphic **stage**, if determined for the stratigraphic unit described (e.g. Zanclean, Piacenzian, Gelasian, Calabrian, Ionian, Tarrantian). Choose carefully from the drop-down menu, and note that new entries are not allowed. See http://www.stratigraphy.org.

The attribute **regional_stage** indicates the name of the regional chronostratigraphic stage, if determined for the stratigraphic unit described. Only use terms valid in the region of investigation (e.g. in the Eastern Mediterranean: Dacian, Romanian, Emilian, Milazzian, Tyrrenian; in the Black Sea region: Kimmerian, Kuyalnikian, Gurian, Chaudian, Euxino-Uzunlarian). Choose carefully from the drop-down menu, but if a regional stage is not available, a new value may be entered. See http://www.stratigraphy.org.

The attribute **formation** indicates the name of the formation, i.e., a locally defined lithostratigraphic unit, if determined for the stratigraphic unit described.

The attribute **mis** signifies the Marine Isotope Stage, if known, for the stratigraphic unit described (see Lisiecki & Raymo 2005). Do not enter "MIS" before the stage number (e.g. 14, 6, 5d). The MIS may be entered as a range with the older value first, followed by a hyphen and no extra spaces (e.g. "15-13", "6-5e" or "5b-3"). If the MIS is "5 or 3", enter the data as "5/3". Choose carefully from the drop-down menu. If an MIS is not available, a new value may be entered.

The attribute **correlation** describes how the chronostratigraphic age of the stratigraphic unit was determined (e.g. archaeological stratigraphy geomorphology, large mammal biostratigraphy, magnetostratigraphy, mollusc biostratigraphy, tephrostratigraphy). If the correlation is based on absolute dating, enter the details in the table geological_layer_age. The attribute **correlation** must be completed if the attributes **age_min** and **age_max** contain data. Otherwise, it will not be clear how **age_min** and **age_max** were determined. Multiples entries for correlation are possible and will be automatically separated by commas. Choose carefully from the drop-down menu. If a correlation is not available, a new value may be entered after consulting with an advisor.

The attribute **magnetostratigraphic_chron** indicates the name of the magnetostratigraphic chron, if determined for the stratigraphic unit described: Gauss(C2An), Matuyama(C1r), Brunhes(C1n). Use a slash if the entry straddles two magnetostratigraphic chrons (e.g. Olduvai(C2n)/Matuyama(C1r), Matuyama(C1r)/Brunhes(C1n), Matuyama(C1r)/Brunhes(C1n). Choose carefully from the drop-down menu, and note that new entries are not allowed.

The attribute **magnetostratigraphic_subchron** indicates the name of the magnetostratigraphic subchron, excursion or event, if determined for the stratigraphic unit described: e.g.

Gauss(C2An.3n), Mammoth(C2An.2r), Gauss(C2An.2n), Kaena(C2An.1r), Gauss(C2An.1n), Matuyama(C2r.2r), Reunion(C2r.1n), Matuyama(C2r.1r), Matuyama(C1r.2r), Jaramillo(C1r.1n), Matuyama(C1r.1r). Use a slash if the entry straddles two magnetostratigraphic subchrons: e.g. Gauss(C2An.3n)/Mammoth(C2An.2r), Matuyama(C1r.2r)/Jaramillo(C1r.1n), Brunhes(C1n)/Matuyama(C1r.1r). Choose carefully from the drop-down menu, and note that new entries are not allowed.

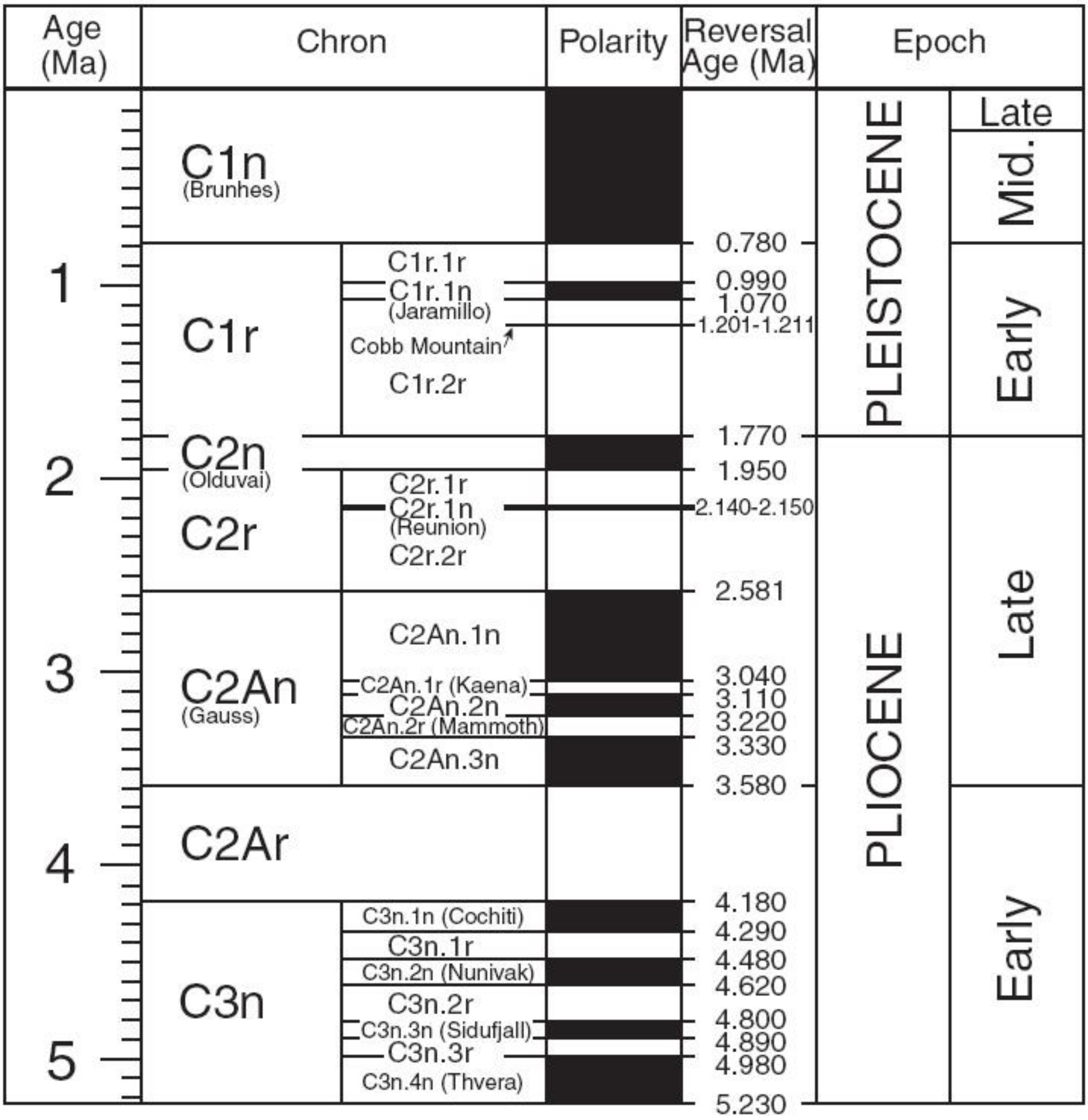

The attribute **biostratigraphy_large_mammals** indicates the name of the biostratigraphic unit based on large mammals, if determined for the stratigraphic unit described.

The attribute **biostratigraphy_small_mammals** indicates the name of the biostratigraphic unit based on small mammals, if determined for the stratigraphic unit described.

The attribute **biostratigraphy_nannoplankton** indicates the name of the biostratigraphic unit based on nannoplankton, if determined for the stratigraphic unit described: NN 15, NN 16, NN 17, NN 18, NN 19, NN 20, NN 21.

The attribute **biostratigraphy_foraminifera** indicates the name of the biostratigraphic unit based on foraminifera, if determined for the stratigraphic unit described: PL 5, PL 6, PT 1a, PT 1b.

The attribute **biostratigraphic_image** represents a link to a visualization of the stratigraphy. The figure can be a sketch, drawing, or photo.

The attribute **comments** contains general comments about the nature of the stratigraphic unit.

The attribute **owner** automatically indicates the last person who entered or changed the dataset.

The attribute **created** automatically indicates the date this dataset was originally entered. An additional attribute **mod_history** automatically records the modification history of a dataset including the names of users who made previous changes and the dates those changes were made.

Table: geostrat_desc_geolayer (geological stratigraphy describes geological layer)

This linking table joins information from two tables, geological_stratigraphy and geological_layer, to create a link between a geological layer and the geological stratigraphy that describes its age determination.

Linking table: geostrat_desc_geolayer			
Attribute	Type	Key	Possible values
geolayer_idlocality	String	Primary key/ Foreign key	[chosen from list] Geelbek_Alice, Hohle Fels
geolayer_name	String	Primary key/ Foreign key	[chosen from list] L23, 3a, VII, SGS
geostrat_idgeostrat	String	Primary key/ Foreign key	[chosen from list] 11, 2, Sibudu_BMOD-OMOD2
owner	String		Fidel Castro
created	Date		[automatic date stamp]

The paired attributes **geolayer_idlocality** and **geolayer_name** create a link to a dataset in the table geological_layer.

The attribute **geostrat_idgeostrat** creates a link to a dataset in the table geological_stratigraphy.

The attribute **owner** automatically indicates the last person who entered or changed the dataset.

The attribute **created** automatically indicates the date this dataset was originally entered. An additional attribute **mod_history** automatically records the modification history of a dataset including the names of users who made previous changes and the dates those changes were made.

Archaeological layers & dating

Table: archaeological_layer

The primary table archaeological_layer characterizes a single archaeological layer within an archaeological profile at a locality where an archaeological assemblage was documented. In some cases, the archaeological layer may be the same as the geological layer. In other cases, an archaeological layer may include only part of one geological layer or extend over several geological layers. An archaeological layer can be defined based on its mean, minimum and maximum thickness and its relationship to other archaeological layers within the same profile.

**Primary table: archaeological_layer**			
Attribute	Type	Key	Possible values
locality_idlocality	String	Primary key/ Foreign key	[chosen from list] Geelbek_Alice, Hohle Fels
name	String	Primary key	AD2, CAL, AH I, AH IIIcf
overlying_archlayer_idlocality	String	Foreign key	[chosen from list]
overlying_archlayer_name	String	Foreign key	[chosen from list]
archstratigraphy_idarchstrat	String	Foreign key	[chosen from list]
average_thickness	Float		0.10
minimum_thickness	Float		0.02
maximum_thickness	Float		0.35
archprofile_name	String		GP26
figure	String		[automatically generated]
comments	String		general comments
owner	String		Isabella Rossellini
created	Date		[automatic date stamp]

The paired attributes **locality_idlocality** and **name** specify the locality and name of the archaeological layer.

The paired attributes **overlying_archlayer_idlocality** and **overlying_archlayer_name** indicate the locality and name of the archaeological layer lying directly above the given layer. In some cases, there may be a gap between two archaeological layers. If a gap is known and its thickness is measured (e.g. 2.2 m sterile), it should be entered as a dummy archaeological layer and defined and described accordingly.

The attribute **archstratigraphy_idarchstrat** contains the variable **idarchstrat** from the look-up table archaeological_stratigraphy. This attribute should be used in order to relate an assemblage to a specific archaeological stratigraphy, if a chronostratigraphic designation is known (e.g. Howiesons Poort, Aurignacian, Sangoan).

The attribute **average_thickness** indicates the average thickness of the archaeological layer in **meters**. Use a period for the decimal place (not a comma).

The attribute **minimum_thickness** indicates the minimum thickness of the archaeological layer in **meters**. Use a period for the decimal place (not a comma).

The attribute **maximum_thickness** indicates the maximum thickness of the archaeological layer in **meters**. Use a period for the decimal place (not a comma).

The attribute **archprofile_name** designates the name of the archaeological profile. If there are two or more archaeological profiles at a locality, then a single ideal profile can be created from the archaeological layers and entered as the "ideal" profile. The ideal profile allows the archaeological layers from different profiles to be correlated with one another. If more than one profile is entered, each profile must have its own unique **archprofile_name** and be correlated using the linking table archlayer_correl_archlayer. Do not use the word "profile" in the name!

The attribute **figure** represents a link to a visualization of the archaeological profile and/or the archaeological layer. The figure can be a sketch, drawing, or photo.

The attribute **comments** contains general comments about the nature of the archaeological layer.

The attribute **owner** automatically indicates the last person who entered or changed the dataset.

The attribute **created** automatically indicates the date this dataset was originally entered. An additional attribute **mod_history** automatically records the modification history of a dataset including the names of users who made previous changes and the dates those changes were made.

Table: archaeological_layer_age

The primary table archaeological_layer_age specifies the age of an archaeological layer based on results reported by an analytical laboratory for a single sample from a specific archaeological layer. Archaeological dating results help determine the age of the archaeological layer. Dating results for a geological layer should be entered in the table geological_layer_age, while dating results for an assemblage should be entered in the table assemblage_age. Only enter a dating result once, regardless of the age table selected. Each dated sample has a unique identification number generated by the laboratory as reported in a publication. The analytical laboratory provides a report containing a result, stated as a number in years before present, and a positive and negative standard deviation which are not always the same. The dating method and the material dated should be specified.

**Primary table: archaeological_layer_age**			
Attribute	Type	Key	Possible values
idarchaeological_layer_age	Integer	Primary key	[automatically generated]
archlayer_idlocality	String	Foreign key	[chosen from list]
archlayer_name	String	Foreign key	[chosen from list]
laboratory_idlaboratory	String	Foreign key	[chosen from list]
analysis_number	String		Pta-2345, GrA-1234
age	Integer		20000, 225000
positive_standard_deviation	Integer		5000
negative_standard_deviation	Integer		4500
date_of_analysis	Date		2007
dating_method	String		OSL
material_dated	String		sand
comments	String		general comments
owner	String		Jörg Weissenborn
created	Date		[automatic date stamp]

The attribute **idarchaeological_layer_age** is a unique, automatically generated, consecutive number assigned to the dataset.

The paired attributes **archlayer_idlocality** and **archlayer_name** indicate the designation of the locality and name of the archaeological layer from the table archaeological_layer.

The attribute **laboratory_idlaboratory** is the official abbreviation for the designated analytical laboratory and should match the entry from the table laboratory. Make sure to follow naming conventions exactly with regard to upper and lower case letters. If the laboratory is not in the table laboratory, you should first enter it there.

The attribute **analysis_number** specifies the official laboratory number assigned to the dated sample (e.g. Pta-2345, GrA-1234, OxA-X-2456-45). Always include the lab prefix, followed by a single hyphen, and then the official number provided by the laboratory. Make sure to follow naming conventions exactly with regard to upper and lower case letters. Use hyphens as separators instead of spaces.

Please note that the **analysis number** is ususally not the same as a sample number! A sample number is often related to a specific locality, profile, layer or square, the year the sample was taken, or the person who took the sample. For example, DRS-E-10 could designate sample number 10 taken from the east profile of Diepkloof Rockshelter, GK-2001-06 could indicate sample 6 taken at Geissenklösterle in 2001, or JK-1559 might signify sample 1559 taken by J. Krause. Sample numbers should rather be entered in the comments, unless no other identifying information for the sample is provided in the publication.

The attribute **age** specifies the result of the dating analysis as reported by the laboratory in years before present. If the result is 142 ka, then enter 142000. For radiocarbon dates, always enter uncalibrated radiocarbon ages. All other dates are entered as years before present (BP). Do not use decimals places or thousand separators such as commas, periods or spaces. Do not enter "+" or "-".

The attribute **positive_standard_deviation** specifies the positive standard deviation of the dating analysis in years. Do not use decimals places or thousand separators such as commas, periods or spaces. Do not enter "+" or "-". Enter a positive standard deviation even if it is the same as the negative standard deviation. If the date is a minimum (or infinite) age, meaning that the dating results are beyond the limits of the specific method, enter "1000000".

The attribute **negative_standard_deviation** specifies the negative standard deviation of the dating analysis in years. Do not use decimals places or thousand separators such as commas, periods or spaces. Do not enter "+" or "-". Enter a negative standard deviation even if it is the same as the positive standard deviation. If the date is a minimum (or infinite) age, meaning that the dating results are beyond the limits of the specific method, enter "0".

The attribute **date_of_analysis** specifies the year of the analysis/report or the first publication of the results in the format yyyy. If the year is unknown, enter "0".

The attribute **dating_method** specifies the method of analysis (e.g. 14C, AMS, TL, OSL, IRSL, ESR, U-Series, Ar/Ar, Amino Acid Racemization, etc.) More detailed information about the dating method can be entered under **comments**.

The attribute **material_dated** specifies the general type of material analyzed (e.g. bone, tooth, antler, ivory, charcoal, ostrich eggshell, shell marine, wood, plant remains, calcrete, lithic, unknown, etc.) More detailed information about the material dated can be entered under **comments**.

The attribute **comments** contains general comments about the dating results.

The attribute **owner** automatically indicates the last person who entered or changed the dataset.

The attribute **created** automatically indicates the date this dataset was originally entered. An additional attribute **mod_history** automatically records the modification history of a dataset including the names of users who made previous changes and the dates those changes were made.

Table: archaeological_stratigraphy

The look-up table archaeological_stratigraphy is a combined stratigraphic depiction, a compilation of archaeological data grouping cultural and technological entities on global and regional scales. The table archaeological_stratigraphy allows the user to choose from a list of pre-selected cultural complexes that have a specific time span associated with them. New entries into this table may be made after approval by project scientists.

**Look-up table: archaeological_stratigraphy**			
Attribute	Type	Key	Possible values
idarchstrat	String	Primary key	ELSA, Howiesonspoort, ESA/MSA
description	String		leaf points, handaxes
age_min	Integer		40000
age_max	Integer		80000
cultural_period	String		Middle Paleolithic
technocomplex	String		Mousterian
regional_technocomplex	String		Blattspitzen
comments	String		general comments
owner	String		Katrina Wodjahoricz
created	String		[automatic date stamp]

The attribute **idarchstrat** is a unique cultural complex that pertains to a specific period of time. Additional archaeological stratigraphies can be added after approval by project scientists.

The attribute **description** provides an archaeological description for the attribute **idarchstrat** (e.g. Classic Wilton: microlithic assemblage with high incidence of backed bladelets and especially segments or crescents, borers, small scrapers on truncated flakes and blades, oval double scrapers with steep retouch along both margins (= double segments), ornaments, polished bone tools).

The attribute **age_min** indicates the minimum age of the cultural complex in years according to the information given in this table. Do not use commas or periods.

The attribute **age_max** indicates the maximum age of the cultural complex in years according to the information given in this table. Do not use commas or periods.

The attribute **cultural_period** specifies the main cultural epoch and includes the Eurasian Paleolithic (Lower, Middle, Upper, Epi) and the African Stone Age (Earlier, Middle, Later).

The attribute **technocomplex** specifies the general technocomplex, including Oldowan, Acheulean, Mousterian, etc.

The attribute **regional_technocomplex** specifies local or regional technocomplexes or subphases, including Blattspitzen, Aurignacian, Howiesons Poort, etc.

The attribute **comments** contains general comments about the archaeological stratigraphy.

The attribute **owner** automatically indicates the last person who entered or changed the dataset.

The attribute **created** automatically indicates the date this dataset was originally entered. An additional attribute **mod_history** automatically records the modification history of a dataset including the names of users who made previous changes and the dates those changes were made.

Table: archlayer_correl_geolayer (archaeological layer correlates with geological layer)

The linking table archlayer_correl_geolayer correlates an archaeological layer with the ideal geological profile.

**Linking table: archlayer_correl_geolayer**			
Attribute	Type	Key	Possible values
archlayer_idlocality	String	Primary key/ Foreign key	[chosen from list] Aar, Bambata Cave
archlayer_name	String	Primary key/ Foreign key	[chosen from list] Layer 1, Bambata
geolayer_idlocality	String	Primary key/ Foreign key	[chosen from list] Aar, Bambata Cave
geolayer_name	String	Primary key/ Foreign key	[chosen from list] Layer 3, Upper cave earth
owner	String		Jo Biden
created	Date		[automatic date stamp]

The paired attributes **archlayer_idlocality** and **archlayer_name** specify the locality and name of the archaeological layer in an archaeological profile.

The paired attributes **geolayer_idlocality** and **geolayer_name** specify the locality and name of the geological layer in the geological profile to which the archaeological layer correlates.

The attribute **owner** automatically indicates the last person who entered or changed the dataset.

The attribute **created** automatically indicates the date this dataset was originally entered. An additional attribute **mod_history** automatically records the modification history of a dataset including the names of users who made previous changes and the dates those changes were made.

Table: archlayer_correl_archlayer (archaeological layer correlates with archaeological layer)

The linking table archlayer_correl_archlayer correlates an archaeological layer with the ideal archaeological profile.

Linking table: archlayer_correl_archlayer			
Attribute	Type	Key	Possible values
archlayer_idlocality_1	String	Primary key/ Foreign key	[chosen from list] Border Cave, Diepkloof Rock Shelter
archlayer_name_1	String	Primary key/ Foreign key	[chosen from list] BACO A, B6 - I6 1st hearth
archlayer_idlocality_2	String	Primary key/ Foreign key	[chosen from list] Border Cave, Diepkloof Rock Shelter
archlayer_name_2	String	Primary key/ Foreign key	[chosen from list] Layer 11b, J6–M6 1st hearth
owner	String		Eric the Red
created	Date		[automatic date stamp]

The paired attributes **archlayer_idlocality_1** and **archlayer_name_1** specify the locality and name of the archaeological layer in the ideal profile.

The paired attributes **archlayer_idlocality_2** and **archlayer_name_2** specify the locality and name of the archaeological layer in the secondary profile to which the ideal archaeological layer correlates.

The attribute **owner** automatically indicates the last person who entered or changed the dataset.

The attribute **created** automatically indicates the date this dataset was originally entered. An additional attribute **mod_history** automatically records the modification history of a dataset including the names of users who made previous changes and the dates those changes were made.

Assemblage & dating

Table: assemblage

The primary table assemblage contains information about classes of finds. An assemblage is defined as a collected find consisting of grouped classes of materials, for example: archaeological finds (including raw material, typology, technology, function, organic tools, symbolic artifacts, feature and miscellaneous finds), human remains, faunal remains or botanical remains. An assemblage comes from a single geological layer at a locality, whether the nature of the locality is geological, archaeological or paleontological. **Each physical object contained in any assemblage can appear only once in this table.** In other words, duplicate entries for finds are not allowed. For each assemblage (geological, paleontological, archaeological, etc.), at least one additional table must be completed to describe that assemblage. In assemblages consisting of stone artifacts, the lithic piece count reflects the number of artifacts contained in the assemblage. In some cases, an assemblage may consist of one item. If an assemblage has coordinates differing from those entered in the table locality, those coordinates can be entered here. The degree to which an assemblage was collected systematically is indicated by scoring the sampling procedure. The representativeness of an assemblage is indicated by scoring the collection bias. An assemblage may be collected during a single year, a series of years, or over discontinuous years.

**Primary table: assemblage**			
Attribute	Type	Key	Possible values
locality_idlocality	String	Primary key/ Foreign key	[chosen from list] Geelbek_Alice, HohleFels
idassemblage	Integer	Primary key	[automatically generated]
name	String		Geelbek_Alice archaeological assemblage
category	String		raw material, paleofauna, humanremains
lithic_piece_count	Integer		5, 178, 2089
coordinates_present	Boolean		true, false
x	Float		12.34
y	Float		-56.78
z	Float		1234.56
is_systematic	Integer		3, 2, 1 or 0
collection_bias	Integer		3, 2, 1 or 0
date	String		1997-1999, 2005
comments	String		general comments
owner	String		George Sands
created	Date		[automatic date stamp]

The paired attributes **locality_idlocality** and **idassemblage** specify the locality and the unique, automatically generated, consecutive number of the dataset.

The attribute **name** consists of a unique entry that identifies the assemblage. It can consist of a find or sample number, an ID number, a local identifier or simply a unique description of an assemblage (e.g. Mumbwa Stratum V Hominids, Layer 30 assemblage, BBC M1 assemblage).

The attribute **category** lists all attribute tables in ROAD that contain further information about the assemblage. An assemblage consisting of human remains would include the entry "human remains". In the case of archaeological assemblages, multiple categories are the norm and may include "raw material, typology, technology, function, organic tools, symbolic artifacts, feature, miscellaneous finds". A faunal assemblage can also contain multiple entries including: "paleofauna, animal remains", while a botanical assemblage can only include the entry "plant remains".

The attribute **lithic_piece_count** represents the total number of stone artifacts (n) included in a lithic assemblage from an archaeological locality. This attribute should only be used for stone artifacts, and not for human, faunal or botanical remains. For example, in an assemblage containing 1000 stone artifacts, the **lithic_piece_count** would be "1000". This number includes the total number of chipped stone artifacts (tools, cores, debitage), unchipped stone artifacts (ground stone tools) and unchipped stones (manuports). If the number is unknown, enter "-1".

The Boolean attribute **coordinates_present** indicates whether x,y,z-coordinates for an assemblage differ from the coordinates of the locality. This may be the case at large localities where assemblages can be collected over broad areas. If additional x,y,z-coordinates are known, then tick the box to indicate this, and be sure to enter the specific coordinates, as described for the attributes **x**, **y**, **z** below. If there are no additional x,y,z-coordinates for the assemblage (i.e. they are not known or do not differ from the main locality), then leave the box unticked to indicate that the assemblage was not collected with specific coordinates. It may be possible that only an elevation (z-coordinate) is known for an assemblage, for example, in the case of a geological profile extending over several kilometers.

The attribute **x** is the geographic longitude of the assemblage in decimal degrees. East longitude is entered as a positive number, west longitude as a negative number. To convert degrees, minutes and seconds into decimal degrees, use the following formula:

$$\frac{\left(\frac{Seconds}{60} + Minutes\right)}{60} + Degrees = Decimal\ Degrees$$

e.g. W 98° 28' 5.2"

$$\frac{\left(\frac{5.2}{60} + 28\right)}{60} + 98 = -98.46811$$

The attribute **y** is the geographic latitude of the assemblage in decimal degrees. North latitude is entered as a positive number, south latitude as a negative number. To convert degrees, minutes and seconds into decimal degrees, use the following formula:

$$\frac{\left(\frac{Seconds}{60} + Minutes\right)}{60} + Degrees = Decimal\ Degrees$$

e.g. N 48° 45.87'

$$\frac{\left(\frac{0}{60} + 45.87\right)}{60} + 48 = 48.7645$$

The attribute **z** is the elevation of a locality in meters above sea level, and can be entered if it is known. Unlike the table locality, the attribute **z** in the table assemblage is used to enter the relative elevation of an assemblage, in meters, with respect to the actual elevation of the locality, as entered in the table locality under the attribute **z**. This attribute is used to enter the depth of individual samples from a profile or boring. If only the "real" elevation of the assemblage is known, then the difference between the elevation of the locality and the elevation of the assemblage should be entered. Positive or negative values may be used. Positive values indicate an elevation above the locality, while negative values are below the elevation of the locality. Use a decimal point to signify values smaller than a meter (e.g. 2.28 or -7.05).

The attribute **is_systematic** indicates the degree to which an assemblage of finds was collected systematically. This attribute attempts to quantify whether an intentional research plan stands behind the collection of finds and therefore ranks the quality of the data. The assemblage is scored as follows:

- 3: the assemblage was collected from an excavation or a controlled collection procedure (e.g. dry sieving, wet screening, pollen retrieval).
- 2: the assemblage results from a survey where exact x,y-coordinates of the locality are known.
- 1: the assemblage results from opportunistic or random surface collections without context.
- 0: the degree of systematic collection is unknown.

The attribute **collection_bias** indicates the conditions under which an assemblage is stored and curated. This attribute attempts to quantify the degree to which the assemblage was affected after collection and ranks the quality of the data. The assemblage is scored as follows:

- 3: the collection is well documented and complete. Complete in a paleontological sense means that the assemblage contains anatomically or taxonomically undetermined specimens. Complete in an archaeological sense means the collection is intact and remains complete.
- 2: the collection is well documented, but incomplete. Incomplete in a paleontological sense means that indeterminate specimens are lacking, while in an archaeological sense, it means the collection was sampled.
- 1: the assemblage results from an arbitrary exchange of specimens between curators.
- 0: means that the quality of the storage conditions remains unknown.

The attribute **date** lists the years in which a given assemblage was collected. The data can be entered as single year, a series of years, or discontinuous years (e.g. 1987, 1989-1994, 2000, 2005).

The attribute **comments** provides additional information about the assemblage.

The attribute **owner** automatically indicates the last person who entered or changed the dataset.

The attribute **created** automatically indicates the date this dataset was originally entered. An additional attribute **mod_history** automatically records the modification history of a dataset including the names of users who made previous changes and the dates those changes were made.

Table: assemblage_age

The primary table assemblage_age specifies the age of an assemblage based on results reported by an analytical laboratory for a single sample from a specific assemblage. Assemblage dating results help determine the age of the assemblage. Dating results for a geological layer should be entered in the table geological_layer_age, while dating results for an archaeological layer should be entered in the table archaeological age. Only enter a dating result once, regardless of the age table selected. Each dated sample has a unique identification number generated by the laboratory as reported in a publication. The analytical laboratory provides a report containing a result, stated as a number in years before present, and a positive and negative standard deviation which are not always the same. The dating method and the material dated should be specified.

**Primary table: assemblage_age**			
Attribute	Type	Key	Possible values
idassemblage_age	Integer	Primary key	[automatically generated]
assemblage_idlocality	String	Foreign key	[chosen from list]
assemblage_idassemblage	Integer	Foreign key	[chosen from list]
laboratory_idlaboratory	String	Foreign key	[chosen from list]
analysis_number	String		Pta-2345, GrA-1234
age	Integer		225000
positive_standard_deviation	Integer		10000
negative_standard_deviation	Integer		9000
date_of_analysis	Date		1987
dating_method	String		OSL
material_dated	String		Sand
comments	String		general comments
owner	String		P.T. Barnum
created	Date		[automatic date stamp]

The attribute **idassemblage_age** specifies a unique, automatically generated, consecutive number of the dataset.

The paired attributes **assemblage_idlocality** and **assemblage_idassemblage** specify the locality and unique, automatically generated, consecutive number of the dataset of the assemblage to which the dated materials belong.

The attribute **laboratory_idlaboratory** is the official abbreviation for the designated analytical laboratory and should match the entry from the table laboratory. Make sure to follow naming conventions exactly with regard to upper and lower case letters. If the laboratory is not in the table laboratory, you should first enter it there.

The attribute **analysis_number** specifies the official laboratory number assigned to the dated sample (e.g. Pta-2345, GrA-1234, OxA-X-2456-45). Always include the lab prefix, followed by a single hyphen, and then the official number provided by the laboratory. Make sure to follow naming conventions exactly with regard to upper and lower case letters. Use hyphens as separators instead of spaces.

Please note that the **analysis number** is ususally <u>not</u> the same as a sample number! A sample number is often related to a specific locality, profile, layer or square, the year the sample was taken, or the person who took the sample. For example, DRS-E-10 could designate sample number 10 taken from the east profile of Diepkloof Rockshelter, GK-2001-06 could indicate sample 6 taken at Geissenklösterle in 2001, or JK-1559 might signify sample 1559 taken by J. Krause. Sample numbers should rather be entered in the comments, unless no other identifying information for the sample is provided in the publication.

The attribute **age** specifies the result of the dating analysis as reported by the laboratory in years before present. If the result is 142 ka, then enter 142000. For radiocarbon dates, always enter <u>uncalibrated</u> radiocarbon ages. All other dates are entered as years before present (BP). Do not use decimals places or thousand separators such as commas, periods or spaces. Do not enter “+” or “-”.

The attribute **positive_standard_deviation** specifies the positive standard deviation of the dating analysis in years. Do not use decimals places or thousand separators such as commas, periods or spaces. Do not enter “+” or “-”. Enter a positive standard deviation even if it is the same as the negative standard deviation. If the date is a minimum (or infinite) age, meaning that the dating results are beyond the limits of the specific method, enter “1000000”.

The attribute **negative_standard_deviation** specifies the negative standard deviation of the dating analysis in years. Do not use decimals places or thousand separators such as commas, periods or spaces. Do not enter “+” or “-”. Enter a negative standard deviation even if it is the same as the positive standard deviation. If the date is a minimum (or infinite) age, meaning that the dating results are beyond the limits of the specific method, enter “0”.

The attribute **date_of_analysis** specifies the year of the analysis/report or the first publication of the results in the format yyyy. If the year is unknown, enter “0”.

The attribute **dating_method** specifies the method of analysis (e.g. 14C, AMS, TL, OSL, IRSL, ESR, U-Series, Ar/Ar, Amino Acid Racemization, etc.) More detailed information about the dating method can be entered under **comments**.

The attribute **material_dated** specifies the general type of material analyzed (e.g. bone, tooth, antler, ivory, charcoal, ostrich eggshell, shell marine, wood, plant remains, calcrete, lithic, unknown, etc.) More detailed information about the material dated can be entered under **comments**.

The attribute **comments** contains general comments about the dating results.

The attribute **owner** automatically indicates the last person who entered or changed the dataset.

The attribute **created** automatically indicates the date this dataset was originally entered. An additional attribute **mod_history** automatically records the modification history of a dataset including the names of users who made previous changes and the dates those changes were made.

Table: assemblage_in_geolayer (assemblage in geological layer)

This linking table joins data from two tables, assemblage and geological_layer, to provide information about which assemblage belongs in which geological layer.

Linking table: assemblage_in_geolayer			
Attribute	Type	Key	Possible values
geolayer_idlocality	String	Primary key/ Foreign key	[chosen from list] Geelbek_Alice, Hohle Fels
geolayer_name	String	Primary key/ Foreign key	[chosen from list] AD2, CAL, AH I, AH IIIcf
assemblage_idlocality	String	Primary key/ Foreign key	[chosen from list] Geelbek_Alice, Hohle Fels
assemblage_idassemblage	Integer	Primary key/ Foreign key	[chosen from list]
owner	String		H.M.S. Pinafore
created	Date		[automatic date stamp]

The paired attributes **geolayer_idlocality** and **geolayer_name** specify the locality and name of the geological layer.

The paired attributes **assemblage_idlocality** and **assemblage_idassemblage** specify the locality and assemblage.

The attribute **owner** automatically indicates the last person who entered or changed the dataset.

The attribute **created** automatically indicates the date this dataset was originally entered. An additional attribute **mod_history** automatically records the modification history of a dataset including the names of users who made previous changes and the dates those changes were made.

Table: assemblage_in_archlayer (assemblage in archaeological layer)

This linking table joins two tables, assemblage and archaeological_layer, to provide information about which assemblage belongs in which archaeological layer.

Linking table: assemblage_in_archlayer			
Attribute	Type	Key	Possible values
archlayer_idlocality	String	Primary key/ Foreign key	[chosen from list] Geelbek_Alice, Hohle Fels
archlayer_name	String	Primary key/ Foreign key	[chosen from list] AD2, CAL, AH I, AH IIIcf
assemblage_idlocality	String	Primary key/ Foreign key	[chosen from list] Geelbek_Alice, Hohle Fels
assemblage_idassemblage	Integer	Primary key/ Foreign key	[chosen from list]
owner	String		Lady Gaga
created	Date		[automatic date stamp]

The paired attributes **archlayer_idlocality** and **archlayer_name** specify the locality and name of the archaeological layer.

The paired attributes **assemblage_idlocality** and **assemblage_idassemblage** specify the locality and assemblage.

The attribute **owner** automatically indicates the last person who entered or changed the dataset.

The attribute **created** automatically indicates the date this dataset was originally entered. An additional attribute **mod_history** automatically records the modification history of a dataset including the names of users who made previous changes and the dates those changes were made.

Archaeology

The following section presents a brief overview of the eight tables into which data about archaeological assemblages can be entered:

Lithic Artifacts

The total number of lithic artifacts is entered in the table assemblage under the attribute **lithic_piece_count**. For all lithic artifacts, data will be entered into three tables: raw_material, typology, technology, even if some of the information is not known. An example for a hypothetical assemblage containing a total of 1000 lithic artifacts is presented below:

1. raw_material – The raw material analysis includes all stone artifacts in an assemblage, whether chipped or not.

The first analysis describes the types of stone used by prehistoric peoples at an archaeological locality. It describes the distance that each raw material was transported and divides them into five categories. The *percentage* of each transport distance should be entered with regard to the total number of artifacts present in the entire assemblage (i.e., 1000).

**Transport distance**	**Percent (%)**	**Raw material list**
Local (0-5 km)	60	quartzite 300, quartz 200, granite 100
Regional (6-20 km)	10	flint 100
Supra-regional (21-100 km)	10	chalcedony 60, jasper 40
Distant (>100 km)	0	none
Unknown	20	hornfels 200

2. typology – Lithic typology includes mainly tools, but may also comprise cores and debitage that have specific forms (e.g. levallois core, levallois point, burin spall).

The second analysis describes the lithic typologies present in an assemblage and divides them into four categories. The *percentage* of each typology should be entered with regard to the total number of artifacts present in the entire assemblage (i.e., 1000).

**Typology**	**Percent (%)**	**Typology list**
Chipped tools	20	backed piece 100, bifacial tool 50, end scraper 30, miscellaneous retouched piece 20
Unchipped tools	5	ground stone tool (e.g. hammerstone, anvil, grindstone) 50
Non-tools	70	all other lithic artifacts that are not tools (e.g. core, debitage) 700
Unknown	5	unknown typology (no information) 50

3. technology – Lithic technology includes mainly cores and debitage, but may also comprise tools that are manufactured using known technologies.

The third analysis describes the lithic technologies present in an assemblage and divides them into four categories. The *percentage* of each technology should be entered with regard to the total number of artifacts present in the entire assemblage (i.e., 1000).

**Technology**	**Percent (%)**	**Technology list**
Chipping technology	60	levallois 300, bladelet 200, flake 100
Non-chipping technology	5	hammerstone 30, grindstone 20
Manuport	5	imported unchipped stone 50
Unknown	30	unknown technology 300

4. function – Functional analysis includes analysis of a selection of stone artifacts for residues and use wear.

If a functional analysis to determine what activities were performed with selected stone artifacts, then a fourth table should be filled in. The fourth type of analysis addresses only the lithic artifacts that have been specifically analyzed for function and use wear. The analysis divides them into five categories. For example in the assemblage of 1000 artifacts, 40 artifacts underwent a functional analysis with the following results.

**Functional analysis**	**Number (n)***	**Residue list**
Microwear traces	10	striation 7, polish 2, unknown 1
Macrowear traces	15	impact fracture 9, use retouch 4, unknown 2
Residues	25	blood 8, feather barbules 5, wood 2, plant fiber 4, starch grain 1, bitumen 1, pitch 1, unknown 3
No traces of function	10	
Not analyzed	960	1000-40

*Note that one artifact can yield several types of traces, or no traces at all.

Non-lithic archaeological artifacts–See table descriptions for more details.

5. organic_tools – Organic tools include bones, teeth, antler, ivory and shell that have been modified or used as tools (e.g. bone artifacts, shell scrapers).
6. symbolic_artifacts – Symbolic artifacts include pieces of art, musical instruments, engraved materials (e.g. ochre, stone, ostrich eggshell), personal ornaments (e.g. beads of shell, stone, ostrich eggshell).
7. miscellaneous_finds – Miscellaneous finds are not lithics or organic tools, but include inorganic materials such as pigments, bitumen, coprolites as well as some unmodified organic materials (e.g. shell).
8. features – Features are a type of find that exists as a concept, but not as a material object that can be brought back to the laboratory. Examples of features include: hearth, burial, structure, floor, pit.

Table: raw_material

The first lithic table, raw_material, provides specific information about the lithic raw materials in an assemblage that are present at a locality. The raw materials of an assemblage are distinguished based on the distance of transport to a locality. A raw material is a stone (e.g. granite, flint or chert) that was gathered by prehistoric people at a certain transport distance away from a locality and used at that locality in the manufacture of lithic artifacts. In this table, there are five possibilities for entering the transport distance. The percentage of raw materials present for each transport distance is calculated with respect to the number of artifacts present in the entire assemblage, that is, the number entered in the attribute **lithic_piece_count** in the table assemblage. The different types of raw materials can be selected from a list of materials, or new typology may be added to the lists only after discussion with the advisors.

**Primary table: raw_material**			
Attribute	Type	Key	Possible values
assemblage_idlocality	String	Primary key/ Foreign key	[chosen from list] Hohle Fels, Geelbek_Homo
assemblage_idassemblage	Integer	Primary key/ Foreign key	[chosen from list]
transport_distance	String	Primary key	local (0-5 km), regional (6-20 km), supra-regional (21-100 km), distant (>100 km), unknown
percentage	Integer		0, 3, 7, 10, 80, -1
raw_material_list	String		quartzite, chert, flint, radiolarite, quartz, basalt, indeterminate
figure	String		[automatically generated]
comments	String		general comments
owner	String		Mikhail Baryshnikov
created	Date		[automatic date stamp]

The paired attributes **assemblage_idlocality** and **assemblage_idassemblage** define the locality and assemblage of the raw material.

The attribute **transport_distance** includes five categories, each distinguished by specific intervals of transport for the raw materials present in an assemblage. When more than one type of transport distance is present, then each of the transport distances must be entered as a unique dataset. The five fixed types of transport distance are:

1. local (0-5 km)
2. regional (6-20 km)
3. supra-regional (21-100 km)
4. distant (>100 km)
5. unknown

The attribute **percentage** indicates what percentage of the raw materials within an assemblage have been carried over a specific transport distance to the locality. Percentages should be rounded to the nearest whole number. Do not use decimal places. If the result is less than 0.5%, enter "0". If the percentage is unknown, enter "-1".

The attribute **raw_material_list** contains a list of raw materials. New raw materials may be added to the lists only after discussion with the advisors, and multiple entries are allowed. Use the value "unknown" when the raw material is indeterminate. The quantity of each type of raw material may be added after the type of raw material (e.g. "silcrete 148" or "quartz 73").

> Raw material list: Possible examples of raw material include: quartz, quartzite, chert, chert jurassic, chert cretaceous, tabular flint, flint baltic, flint tertiary, radiolarite, basalt, granite, obsidian, unknown, etc.

The attribute **figure** represents a link to a visualization of the raw material. The figure can be a sketch, drawing, or photo.

The attribute **comments** contains general comments about the nature of the raw material.

The attribute **owner** automatically indicates the last person who entered or changed the dataset.
The attribute **created** automatically indicates the date this dataset was originally entered. An additional attribute **mod_history** automatically records the modification history of a dataset including the names of users who made previous changes and the dates those changes were made.

Table: typology

The second lithic table, typology, provides information about the types of lithic artifacts present at a locality. Typology mainly concerns chipped and unchipped stone tools, although certain core and debitage types may be included here as non-tool forms. Chipped tools are defined as intentionally retouched products (e.g. scraper, backed blade, drill), while unchipped tools are rather a result of usage (e.g. hammerstone, grinder, anvil). The percentage of artifacts that have no tool typology is also quantified. Four categories are possible for the basic typologies: chipped tool, non-chipped tool, non-tool and unknown. The percentage of tool types present for each typology is calculated with respect to the number of artifacts present in the entire assemblage, that is, the number entered in the attribute **lithic_piece_count** in the table assemblage. Note that debitage and waste products of tool production are generally not considered here, but rather in the attribute **product_list** in the table technology. Stone ornaments and art objects made of stone are also not considered here, but instead appear in the table symbolic_artifacts.

**Primary table: typology**			
Attribute	Type	Key	Possible values
assemblage_idlocality	String	Primary key/ Foreign key	[chosen from list]<br>Hohle Fels, Geelbek_Homo
assemblage_idassemblage	Integer	Primary key/ Foreign key	[chosen from list]
typology	String	Primary key	chipped tool, non-chipped tool, non-tool, unknown
percentage	Integer		0, 15, 5, 80, -1
tool_list	String		chipped tools<br>end scraper, handaxe, leafpoint, chopper, unknown, etc.<br>non-chipped tools<br>retoucher, hammerstone, grinding stone, unknown, etc.
figure	String		[automatically generated]
comments	String		general comments
owner	String		Nancy Reagan
created	Date		[automatic date stamp]

The paired attributes **assemblage_idlocality** and **assemblage_idassemblage** define the locality and assemblage of the typology.

The attribute **typology** describes groups of tool types present in the lithic assemblage. The four possible entries are chipped tool, non-chipped tool, non-tool and unknown. No other entries are possible. Tools produced by chipping might include end scraper, handaxe and bifacial point. Tools produced by non-chipping could include hammerstone, anvil and grindstone. Non-tools comprise all unretouched stone artifacts including most cores and debitage. Unknown includes unused or battered cobbles, blocks and manuports. New typologies may not be added to the list, and multiple entries are not allowed in the same dataset. When more than one type of typology is present, then each type must be entered as a unique dataset. The four fixed types of **typology** are:

1. chipped tool
2. non-chipped tool
3. non-tool
4. unknown

The attribute **percentage** indicates what percentage of a type of typology is present in a given lithic assemblage. Percentages should be rounded to the nearest whole number. Do not use decimal places. If the result is less than 0.5%, enter "0". If a percentage is unknown, enter "-1".

The attribute **tool_list** contains values that can be entered for various tool types. The first list contains chipped tool types, while the second list contains non-chipped tool types. The third and fourth lists contain the types for non-tools and unknown. New typology may be added to the lists only after discussion with the advisors, and multiple entries are allowed. Use the value "'unknown" when the typology is indeterminate. The quantity of each type may be added after the typology (e.g. "scraper end 23" or "chopper 5").

1. chipped tool: scraper end, scraper side, scraper carinated, burin, handaxe, chopper, cleaver, point, point unifacial, segment, unknown, etc.
2. non-chipped tool: grindstone upper, hammerstone, anvil, retoucher, unknown, etc.
3. non-tool: core, debitage, flake, point, etc.
4. unknown: cobble, block, manuport, unknown, etc.

The attribute **figure** represents a link to a visualization of the typology. The figure can be a sketch, drawing, or photo.

The attribute **comments** contains general comments about the nature of the typology.

The attribute **owner** automatically indicates the last person who entered or changed the dataset.

The attribute **created** automatically indicates the date this dataset was originally entered. An additional attribute **mod_history** automatically records the modification history of a dataset including the names of users who made previous changes and the dates those changes were made.

Table: technology

The third lithic table, technology, differentiates how stone artifacts were manufactured. Technology encompasses mainly cores, blanks and debitage, although certain chipped and non-chipped tools are also included here. Four categories of technology are available: chipping technology, non-chipping technology, manuport and unknown. The percentage of artifacts present for each technology is calculated with respect to the number of artifacts present in the entire assemblage, that is, the number entered in the attribute **lithic_piece_count** in the table assemblage. Stone ornaments and art objects made of stone are not considered here, but rather in the table symbolic_artifacts.

**Primary table: technology**			
Attribute	Type	Key	Possible values
assemblage_idlocality	String	Primary key/ Foreign key	[chosen from list] Hohle Fels, Geelbek_Homo
assemblage_idassemblage	Integer	Primary key/ Foreign key	[chosen from list]
technology	String	Primary key	chipping, non-chipping technology, manuport, unknown
percentage	Integer		0, 2, 17, 81, -1
product_list	String		core, blade, flake, point, debitage, burin spall, manuport, unknown
technology_type	String		levallois method, blade production, soft hammer, unknown
figure	String		[automatically generated]
comments	String		general comments
owner	String		Otto von Bismarck
created	Date		[automatic date stamp]

The paired attributes **assemblage_idlocality** and **assemblage_idassemblage** define the locality and assemblage of the technology.

The attribute **technology** indicates whether a lithic product was produced by chipping or non-chipping technology. An unmodified lithic object brought to a locality by humans would be treated as a manuport, or the technology may be unknown. New technologies may not be added to the list, and multiple entries are not allowed in the same dataset. If more than one type of technology is present, then each type should be entered as a unique dataset. The four fixed types of **technology** are:

1. chipping technology
2. non-chipping technology
3. manuport
4. unknown

The attribute **percentage** indicates what percentage of a technology type is present in a given lithic assemblage. Percentages should be rounded to the nearest whole number. Do not use decimal places. If the result is less than 0.5%, enter "0". If a percentage is unknown, enter "-1".

The attribute **product_list** contains a list of products that can be achieved during the chipping of lithic materials. New products may be added to the list only after discussion with the advisors, and multiple entries are allowed. Use the value "'unknown"' when the product is indeterminate. The quantity of each product may be added after the product (e.g. "core blade 35" or "point 16").

1. Chipping technology: core, core bladelet, core levallois, blade, bladelet, flake, point, debitage, bladelet burin spall, etc.
2. Non-chipping technology: grindstone upper, hammerstone, anvil, retoucher, unknown, etc.
3. Manuport: cobble, block, manuport, etc.
4. Unknown: unknown, i.e. no information available on technology.

The attribute **technology_type** contains different methods used in manufacturing stone artifacts. New types of technology may be added to the list only after discussion with the advisors, and multiple entries are allowed. Use the value "'unknown"' when the type of technology is indeterminate. The quantity of each type of technology may be added after the type of technology (e.g. "discoid method 25" or "bladelet production 54").

Technology type: levallois method, discoid method, blade production, bladelet production, flake production, unidirectional blank production, bidirectional blank production, soft hammer, hard hammer, tool production, unknown, etc.

The attribute **figure** represents a link to a visualization of the technology. The figure can be a sketch, drawing, or photo.

The attribute **comments** contains general comments about the nature of the technology.

The attribute **owner** automatically indicates the last person who entered or changed the dataset.

The attribute **created** automatically indicates the date this dataset was originally entered. An additional attribute **mod_history** automatically records the modification history of a dataset including the names of users who made previous changes and the dates those changes were made.

Table: function

The fourth lithic table, function, examines functional traces on stone artifacts that help determine what tasks they performed. Possible entries include microwear traces, macrowear traces, residues, no traces of function and not analyzed. The total number of each type of functional trace is entered in a separate dataset. Different functions inferred from the traces are listed in a function list. The number of lithic artifacts with the given functional trace can be entered. The number of pieces analyzed for functional traces can be entered in the attribute comments.

**Primary table: function**			
Attribute	Type	Key	Possible values
assemblage_idlocality	String	Primary key/ Foreign key	[chosen from list]<br>Hohle Fels, Geelbek_Homo
assemblage_idassemblage	Integer	Primary key/ Foreign key	[chosen from list]
functional_traces	String	Primary key	microwear trace, macrowear trace, residue, no trace of function, not analyzed
number	Integer		3, 12, 20, 150, -1
function_list	String		cutting, wood working, projectile, unknown
functional_trace_list	String		microwear trace<br>striation, polish, unknown<br>macrowear trace<br>impact fracture, unknown<br>residue<br>blood, feather barbule, wood, bitumen, unknown
figure	String		[automatically generated]
comments	String		general comments
owner	String		Penelope Cruz
created	Date		[automatic date stamp]

The paired attributes **assemblage_idlocality** and **assemblage_idassemblage** define the locality and assemblage of the functional analysis.

The attribute **functional_traces** can contain one of five categories indicating whether microwear traces, macrowear traces or residues are present on stone tools. It is also possible that no traces are present. An entry should also be made for the artifacts that were not analyzed. New functional traces may not be added to the list and multiple entries are not allowed in the same dataset. If more than one type of functional trace is present, then each type should be entered as a unique dataset. The five fixed types of **functional traces** are:

1. microwear traces
2. macrowear traces
3. residues
4. no traces of function
5. not analyzed

The attribute **number** indicates the number of lithic finds in an assemblage which show functional traces. If the exact number is not known, the value "-1" is entered. In order to know how many lithic artifacts were subjected to functional analysis, it is important to enter the number of lithic artifacts in the assemblage which were "not analyzed".

The attribute **function_list** contains a list of functions inferred from the functional traces. New functions may be added to the list only after discussion with the advisors, and multiple entries are allowed. The quantity of each function may be added <u>after</u> the function (e.g. "meat processing 4" or "projectile 18"). The value "'unknown"' is possible.

> <u>Function list</u>: scraping, cutting, hard material processing, soft material processing, wood working, meat processing, hide working, projectile, unknown, etc.

The attribute **functional_trace_list** includes five categories containing the same value list. New functions may be added to the lists only after discussion with the advisors, and multiple entries are allowed. The quantity of each functional trace may be added <u>after</u> the functional traces (e.g. "striation 4, impact fracture 7"), or the functional traces may appear without a number (e.g. "striation, impact fracture").

1. <u>Microwear traces</u>: striation, polish, unknown, etc.
2. <u>Macrowear traces</u>: impact fracture, use retouch, unknown, etc.
3. <u>Residues</u>: blood, feather barbule, wood residue, plant fiber, starch granule, bitumen, pitch, unknown, etc.
4. <u>No traces of function</u>
5. <u>Not analyzed</u>

The attribute **figure** represents a link to a visualization of the functional analysis. The figure can be a sketch, drawing, or photo.

The attribute **comments** contains general comments about the nature of the functional analysis.

The attribute **owner** automatically indicates the last person who entered or changed the dataset.

The attribute **created** automatically indicates the date this dataset was originally entered. An additional attribute **mod_history** automatically records the modification history of a dataset including the names of users who made previous changes and the dates those changes were made.

Table: organic_tools

The primary table organic_tools contains information about artifacts manufactured from an organic raw material, such as antler, bone, ivory, shell, tortoise shell, tusk, or wood. Art objects, musical instruments and ornaments are not considered to be organic tools, but rather belong in the table symbolic_artifacts. Each organic raw material of a tool must be entered separately. The number of tools manufactured from an organic raw material should also be recorded. The interpretation of the tool and the technology used to manufacture it should be selected from a list.

**Primary table: organic_tools**			
Attribute	Type	Key	Possible values
assemblage_idlocality	String	Primary key/ Foreign key	[chosen from list] Geelbek_Stella, Hohle Fels
assemblage_idassemblage	Integer	Primary key/ Foreign key	[chosen from list]
organic_raw_material	String	Primary key	antler, bone, ivory, shell, tortoise shell, tusk, wood, etc.
number	Integer		5
interpretation	String		projectile point, awl
technology	String		abraded, carved, engraved, flaked, ground, incised, etc.
figure	String		[automatically generated]
comments	String		general comments
owner	String		Rachid Taha
created	Date		[automatic date stamp]

The paired attributes **assemblage_idlocality** and **assemblage_idassemblage** define the locality and assemblage of the organic tool.

The attribute **organic_raw_material** indicates the material from which an organic tool is made. Possible values for organic raw materials may be selected from a list and include: antler, bone, ivory, shell, tortoise shell, tusk, wood, etc. New materials may be added to the list only after discussion with the advisors, but multiple entries are not allowed here. If more than one organic raw material is present in an assemblage, then an additional dataset for each organic raw material should be entered. If the organic raw material cannot be determined, enter bone as the general organic raw material and add a comment listing the possible organic raw materials (e.g. bone, antler, ivory).

The attribute **number** indicates the number of individual pieces for each organic raw material in the assemblage.

The attribute **interpretation** contains a list of organic tool types. Possible values for the interpretation may be selected from a list and include: lance/spear, needle eyed, percussor, pin, point, point barbed, point split based, pointed artifact, punch, retoucher, rod, scraper, shaft, etc. New interpretations may be added to the list only after discussion with the advisors, and multiple entries are allowed. The quantity of each interpretation may be added after the artifact type (e.g. “needle eyed 2” or “lance/spear 6”).

The attribute **technology** contains a list of actions used to manufacture organic tools. Possible values for the technology may be selected from a list and include: abraded, carved, engraved, flaked, ground, incised, modified, notched, polished, retouched, unknown, etc. New technologies may be added to the list only after discussion with the advisors, and multiple entries are allowed. The quantity of each technology may be added after the artifact type (e.g. “ground 4” or “incised 2”).

The attribute **figure** represents a link to a visualization of the organic tool. The figure can be a sketch, drawing, or photo.

The attribute **comments** contains general information about the nature of the organic tool. Comments should be used to specify further details about the general standardized information, for literature citations, or to express doubt about an organic tool.

The attribute **owner** automatically indicates the last person who entered or changed the dataset.

The attribute **created** automatically indicates the date this dataset was originally entered. An additional attribute **mod_history** automatically records the modification history of a dataset including the names of users who made previous changes and the dates those changes were made.

Table: symbolic_artifacts

The primary table symbolic_artifacts includes artifacts that convey an abstract meaning. This category includes objects that represent art, music and/or ornaments. Organic tools such as bone points or harpoons are not entered here. The material represents the material of the object, for example, in the case of a bone flute, the material is bone; for a shell bead, the material is shell. However, the material can also be the material onto which an object is made. For example, in the case of a cave painting, the material is the cave wall: the pigment used to make the painting should be entered in the table miscellaneous finds. The material can be: antler, bone, cave wall, clay, ivory, ochre, ostrich eggshell, shell, stone and tooth. The category of the symbolic artifact is defined as art, music and/or ornament, where multiple categories are possible. The interpretation provides further details about the nature of the symbolic artifact, such as abstract, anthropomorphic, instrument, ornament, therianthropic, zoomorphic. The technology used to manufacture the symbolic artifact should be selected from a list. If known, the raw material source of the miscellaneous find material can be indicated, similar to **transport_distance** in the table raw_material. However, here, you may select more than one transport distance.

**Primary table: symbolic_artifacts**			
Attribute	Type	Key	Possible values
assemblage_idlocality	String	Primary key/ Foreign key	[chosen from list] Geelbek_Stella, Hohle Fels
assemblage_idassemblage	Integer	Primary key/ Foreign key	[chosen from list]
material	String	Primary key	antler, bone, cave wall, clay, ivory, ochre, ostrich eggshell, shell, etc.
category	String		art, music, ornament
interpretation	String		abstract, anthropomorphic, instrument, ornament, etc.
technology	String		abraded, carved, engraved, flaked, ground, incised, etc.
raw_material_source	String		local (0-5 km), regional (6-20 km), supra-regional (21-100 km), distant (>100 km), unknown
figure	String		[automatically generated]
comments	String		general comments
owner	String		Stella Artois
created	Date		[automatic date stamp]

The paired attributes **assemblage_idlocality** and **assemblage_idassemblage** define the locality and assemblage of the symbolic artifact.

The attribute **material** represents the material of the object or the material onto which the object is made, for example: antler, bone, cave wall, clay, ivory, ochre, ostrich eggshell, shell, stone,

tooth. New materials may be added to the list only after discussion with the advisors, but multiple entries are not allowed here. If more than one material is present in an assemblage, then additional datasets for those materials should be entered.

The attribute **category** classifies symbolic artifacts into three different groups: art, music and/or ornament. Multiple categories may be selected from the list.

The attribute **interpretation** contains a list of symbolic artifacts. Possible values for the interpretation may be selected from a list and include: abstract, anthropomorphic, instrument, ornament, therianthropic, zoomorphic. New interpretations may be added to the list only after discussion with the advisors, and multiple entries are allowed. The quantity of each interpretation may be added after the chosen interpretation (e.g. "anthropomorphic 1" or "ornament 8").

The attribute **technology** includes a list of technologies such as: painting, engraving, carving, molding, polishing, imprinting, indeterminate, etc. New technologies may be added to the list, and multiple entries are allowed. If the technology is unknown, "indeterminate" should be entered. The quantity of each technology may be added after the technology (e.g. "painting 8" or "carving 2").

The attribute **raw_material_source** includes five fixed categories, each distinguished by specific intervals of transport for the raw material used to manufacture the symbolic artifact. If more than one raw material source is present, you may select more than one transport distance. The five fixed types of raw material source are:

1. local (0-5 km)
2. regional (6-20 km)
3. supra-regional (21-100 km)
4. distant (>100 km)
5. unknown

The attribute **figure** represents a link to a visualization of the symbolic artifact. The figure can be a sketch, drawing, or photo.

The attribute **comments** contains general comments about the nature of the symbolic artifact. Comments should be used to specify further details about the general standardized information, for literature citations, or to express doubt about a symbolic artifact.

The attribute **owner** automatically indicates the last person who entered or changed the dataset.

The attribute **created** automatically indicates the date this dataset was originally entered. An additional attribute **mod_history** automatically records the modification history of a dataset including the names of users who made previous changes and the dates those changes were made.

Table: miscellaneous_finds

The primary table miscellaneous_finds includes finds that cannot be assigned to any of the other existing tables. Miscellaneous finds are classified by their material and may include: beeswax, bitumen, clay, flax fiber, fossil, mineral diverse, mineral pigment, ostrich eggshell, shell, stone, etc. The number of each material of the miscellaneous find can be specified. If known, the raw material source of the material of the miscellaneous find can be indicated, similar to **transport_distance** in the table raw_material. However, here, you may select more than one transport distance.

**Primary table: miscellaneous_finds**			
Attribute	Type	Key	Possible values
assemblage_idlocality	String	Primary key/ Foreign key	[chosen from list] Geelbek_Stella, Hohle Fels
assemblage_idassemblage	Integer	Primary key/ Foreign key	[chosen from list]
material	String	Primary key	beeswax, bitumen, clay, flax fiber, fossil
number	Integer		5
raw_material_source	String		local (0-5 km), regional (6-20 km), supra-regional (21-100 km), distant (>100 km), unknown
figure	String		[automatically generated]
comments	String		general comments
owner	String		Tennessee Williams
created	Date		[automatic date stamp]

The paired attributes **assemblage_idlocality** and **assemblage_idassemblage** define the locality and assemblage of miscellaneous finds.

The attribute **material** indicates the material of the miscellaneous find and may include: beeswax, bitumen, clay, flax fiber, fossil, mineral diverse (materials such as crystals), mineral pigment (colorants such as ochre, hematite, limonite, goethite, specularite, etc.) ostrich eggshell, shell, stone, etc. New materials may be added to the list only after discussion with the advisors, but multiple entries are not allowed here. If more than one type of material is present in an assemblage, then additional materials for those finds should be entered separately.

The attribute **number** indicates how many individual pieces of a given material are present in an assemblage of miscellaneous finds.

The attribute **raw_material_source** includes five fixed categories, each distinguished by specific intervals of transport for the raw material used to manufacture the miscellaneous find category. If more than one raw material source is present, you may select more than one transport distance. The five fixed types of raw material source are:

1. local (0-5 km)
2. regional (6-20 km)
3. supra-regional (21-100 km)
4. distant (>100 km)
5. unknown

The attribute **figure** represents a link to a visualization of the miscellaneous find. The figure can be a sketch, drawing, or photo.

The attribute **comments** contains general information about the nature of the miscellaneous find. Comments should be used to specify further details about the general standardized information, for literature citations, or to express doubt about a miscellaneous find. If the exact source of raw material is known, it can also be entered as a comment.

The attribute **owner** automatically indicates the last person who entered or changed the dataset.

The attribute **created** automatically indicates the date this dataset was originally entered. An additional attribute **mod_history** automatically records the modification history of a dataset including the names of users who made previous changes and the dates those changes were made.

Table: feature

The primary table feature describes non-portable artifacts found at an archaeological locality and includes: bedding, burial, butchering event, combustion feature, cupule, dumping area, dwelling, impact feature, mine, pit, post hole, shell midden, stone construction, windbreak, or other artifacts that cannot be recovered as typical finds. We expand the definition of feature to include collected or grouped set of finds (e.g. the ochre processing toolkit from Blombos Cave which includes a shell containing ochre powder, bone, charcoal and other elements). Finds contained within a feature should be entered in their respective tables, for example, the shell and ochre from Blombos Cave should also be entered in the table miscellaneous finds. Each interpretation of a feature must be entered separately.

**Primary table: feature**			
Attribute	Type	Key	Possible values
assemblage_idlocality	String	Primary key/ Foreign key	[chosen from list] Geelbek_Stella, Hohle Fels
assemblage_idassemblage	Integer	Primary key/ Foreign key	[chosen from list]
interpretation	String	Primary key	bedding, burial, butchering event, combustion feature, cupule, etc.
figure	String		[automatically generated]
comments	String		general comments
owner	String		Quentin Crisp
created	Date		[automatic date stamp]

The paired attributes **assemblage_idlocality** and **assemblage_idassemblage** define the locality and assemblage of the feature.

The attribute **interpretation** defines the feature present in the archaeological assemblage. Examples include: bedding, burial, butchering event, combustion feature, cupule, dumping area, dwelling, impact feature, mine, pit, post hole, shell midden, stone construction, windbreak, etc. New interpretations may be added to the list only after discussion with the advisors. If more than one type of interpretation is present, then each interpretation should be entered as a unique dataset. If known, the number of interpreted features may be entered under comments.

The attribute **figure** represents a link to a visualization of the feature. The figure can be a sketch, drawing, or photo.

The attribute **comments** contains general information about the nature of the feature. Comments should be used to specify further details about the general standardized information, for literature citations, or to express doubt about a feature.

The attribute **owner** automatically indicates the last person who entered or changed the dataset.

The attribute **created** automatically indicates the date this dataset was originally entered. An additional attribute **mod_history** automatically records the modification history of a dataset including the names of users who made previous changes and the dates those changes were made.

Cognition

Table: cognition

The analytical table cognition presents types of cognition that are manifested by the different categories of archaeological remains listed in the archaeological tables: raw material, typology, technology, function, organic tools, symbolic_artifacts, miscellaneous finds and feature. From these artifacts, different and independent cognitive characters can be deduced (e.g. by using the cognigram method). Object behavior encompasses a certain time span of planning depth. The shortest time span involves an acute (non-delayed) problem solution and ranges up to a solution of principal problems where no acute inducement exists. In some objects a symbolic perception is present, or a symbolic representation is manifested, and varies in form. The use or manufacture of tools can also require recursive elements. A tool may be produced without another tool, with the help of a second tool, with a second tool that was produced with a third tool, etc. The length of the analogous effective chain increases accordingly from 1 to 2 to 3, etc. Certain concepts of problem solution can be used with a different flexibility: from one solution for exactly one problem (1:1), to several solutions for one problem (n:1), to one solution for several problems (1:n) to several solutions for a whole group of problems (n:m). Modular behavior can be expressed in different forms: in simple tools, in combined tools, in complex tools or in the transformation of raw materials.

**Analytical table: cognition**			
Attribute	Type	Key	Possible values
assemblage_idlocality	String	Primary key/ Foreign key	[chosen from list] Geelbek_Alice, Hohle Fels
assemblage_idassemblage	Integer	Primary key/ Foreign key	[chosen from list]
cognition_type	String	Primary key	time depth, effective chain
cognition_based_on	String		typology–tool list handaxe; raw material–transport distance distant; technology art–soft hammer use
method	Boolean		true, false
time_depth	String		acute, semi-acute, in principle
symbolic_perception_expression	String		perception ornamental, perception iconic, expression ornamental, expression iconic, expression indexical, expression arbitrary single symbol, expression arbitrary symbolic system
recursion	Boolean		true, false
effective_chain	Integer		minimum number of tool elements
flex_problem_solution_concepts	String		1:1, 1:n, n:1, n:m

modular behaviour	String		unverifiable, simple artifacts, combination of artifacts, complex artifacts, transformation of raw material
figure	String		[automatically generated]
comments	String		general comments
owner	String		Ulysses S. Grant
created	Date		[automatic date stamp]

The paired attributes **assemblage_idlocality** and **assemblage_idassemblage** define the locality and assemblage for the type of cognition.

The attribute **cognition_type** indicates the type of cognition specified and may include: time depth, symbolic perception/expression, recursion, effective chain, flexibility of problem solution-concepts, and modular behavior. New types of cognition may be added to the list, but multiple entries are not allowed here. If more than one type of cognition is specified for an assemblage, then an additional dataset for that type of cognition should be entered.

The attribute **cognition_based_on** refers to the type of archaeological evidence from which the cognitive type is deduced. It consists of the general type of evidence (e.g. raw material, typology, technology, function, organic tools, symbolic artifacts, feature and miscellaneous finds) and if possible, a more detailed description (e.g. typology– tool list handaxe; raw material–transport distance distant; technology art–soft hammer use).

The attribute **method** indicates whether the cognitive manifestation was examined with the help of a cognigram. The usual value for **method** = "false".

The attribute **time_depth** has three fixed values indicating the maximum time range of planning depth that can be concluded from a problem-solution context.

1. acute = action to solve an acute problem, no evidence for delay of problem solution.
2. semi-acute (increased presence/near future) = action to solve a contemporary, but not immediate problem, evidence of short delay of problem solution (e.g. by transport of raw material over several kilometers).
3. in principle = action to solve a distant or principle problem, evidence of marked delay in problem solution (e.g. by supra-regional transport of raw material, stock-keeping, etc.).

The attribute **symbolic_perception_expression** involves a characterization of symbolic perception or symbolic representation. These are manifested in seven fixed object behaviors:

1. ornamental perception (e.g. an unmodified mineral or fossil).
2. iconic perception (e.g. perception of the similarity of a natural object with an animal or human figure, as suggested by minor to moderate modifications that help to enhance the perception of the object, for example, the Berekhat Ram figurine).
3. ornamental representation (creation of an ornamental design, such as the pattern on the Blombos ochre or in the case of Quneitra).
4. iconic representation (creation of an iconic image, such as an animal figurine, or the re-creation of a deer canine made of bone).
5. indexical representation (creation of an indexical image, for example, hand negatives, vulvas).
6. representation of a single arbitrary symbol.
7. representation of an arbitrary system of symbols (e.g. scripture).

A value for **symbolic_perception_expression** or representation is not necessarily required.

The attribute **recursion** indicates whether a tool behavior incorporates recursive elements that refer to mental templates and adjusts the behavior by synchronizing template and work piece. A simple, unmodified flake would receive a value = "false", while the shaping of a handaxe would receive a value = "true". The usual value for **recursion** = "false".

The attribute **effective_chain** describes the minimal number of necessary tools that are consecutively arranged in a hierarchy in order to reach a certain aim:

1. effective chain = 1, using a digging stick and, after that, a probe to catch termites.
2. effective chain = 2, using a hammerstone to produce a flake to dismember a cadaver.

A value for **effective_chain** is not necessarily required.

The attribute **flex_problem_solution_concepts** describes four fixed possibilities for the use of flexible problem-solution concepts:

1. 1:1–a certain solution is used for only a certain problem.
2. 1:n–a certain solution is used for several problems.
3. n:1–several solutions are used for a certain problem.
4. n:m–several solutions are used for a group of problems.

A value for **flex_problem_solution_concepts** is not necessarily required.

The attribute **modular_behaviour** describes five fixed levels of modular tool behavior:

1. not verifiable
2. simple artifacts (e.g. a spear: modular behavior necessary for production).
3. combination of artifacts (e.g. needle and thread, spear thrower and spear).
4. complex artifacts (e.g. shafted tools).
5. transformation of raw material (e.g. birch tar, heat tempered chert, ceramic).

The usual value for **modular_behaviour** = "not verifiable".

The attribute **figure** represents a link to a visualization of the type of cognition or the cognigram. The figure can be a sketch, drawing, or photo.

The attribute **comments** contains general comments about the cognitive meaning of the find.

The attribute **owner** automatically indicates the last person who entered or changed the dataset.

The attribute **created** automatically indicates the date this dataset was originally entered. An additional attribute **mod_history** automatically records the modification history of a dataset including the names of users who made previous changes and the dates those changes were made.

Paleoanthropology

Table: humanremains

The primary table humanremains describes human fossil finds. A human remain is a direct and substantial piece of evidence for the presence of fossil hominids at a particular locality. The human remains table is a dataset that describes such fossils. The chart comprises mainly fossilized remains including parts of the cranium, teeth and postcranial bones. Indirect evidence such as fossil endocasts and footprints are included in this table, and an image of the human remain can be linked.

**Primary table: humanremains**			
Attribute	Type	Key	Possible values
assemblage_idlocality	String	Primary key/ Foreign key	[chosen from list] Geelbek_Stella, Hohle Fels
assemblage_idassemblage	Integer	Primary key/ Foreign key	[chosen from list]
idhumanremains	Integer	Primary key	[automatically generated]
accession_number	String		OH 8, KNM-ER-405
skeletal_element	String		upper L M1, R humeral epiphysis, natural endocast
category	String		C; C,P; C,D,I; E; E,I; C,P,D,E,I
pathologies	String		abscess on root of M2, periodontitis, osteophytes on L3
anthropogenic_modification	String		burnt, cut marks
figure	String		[automatically generated]
figurepdf_image_idimage	Integer	Foreign key	[chosen from list]
comments	String		general comments
owner	String		Veronika Voss
created	Date		[automatic date stamp]

The paired attributes **assemblage_idlocality** and **assemblage_idassemblage** define the locality and assemblage of the human remains.

The attribute **idhumanremains** is an automatically generated, consecutive number of the dataset.

The attribute **accession_number** contains the identification number attributed to a fossil during excavation or collection. Possible values are, OH 8, KNM-ER-405 or KRM 41040, etc.

The attribute **skeletal_element** is an anatomical designation and contains a brief description of the fossil described in the dataset. Cranial and postcranial bones are named by their anatomical appellation in Latin, for example: humerus, (os) capitatum, (os) zygomaticum, os coxae, etc. Left and right are denoted as "L" and "R". Teeth are abbreviated by letters (M = molars, P = premolars, I = incisors, C = canines) combined with Arabic numerals ([automatically generated]) to definitively assign their position in the dental arch. The appellations "'upper" and "lower" are not expressed through a subscript or superscript number but written as words. Possible examples include: upper L M1, R humeral epiphysis, natural endocast, etc.

The attribute **category** indicates the element group to which the fossil belongs. Five categories exist, and new categories may not be added to the list:

- C for cranial bones.
- D for dental remains.
- P for postcranial bones.
- E for natural endocasts.
- I for imprints that are negative molds of body parts, including footprints.

Multiple entries are allowed and should be entered with a comma and a single space separating the values.

The attribute **pathologies** describes possible pathologic evidence (e.g. deformations, porosities) observed on the human fossils. If possible, the entry should contain the medical name of the affliction that caused the observed mark and its anatomical location. Possible entries are "periodontitis on the upper L M3" or "osteophytes on L side of corpus vertebrae of T8", etc.

The attribute **anthropogenic_modification** lists anthropogenic deformations of the fossil that occurred before fossilization. Possible entries are "burnt" for fire marks on fossils or "cut marks" for striations on bone, etc.

The attribute **figure** represents a link to a visualization of the human remain. The figure can be a sketch, drawing, or photo.

The attribute **figurepdf_image_idimage** links the copyrighted image to the human remain.

The attribute **comments** contains general comments about the nature of the human remain.

The attribute **owner** automatically indicates the last person who entered or changed the dataset.

The attribute **created** automatically indicates the date this dataset was originally entered. An additional attribute **mod_history** automatically records the modification history of a dataset including the names of users who made previous changes and the dates those changes were made.

Table: scientist_desc_humanremains (scientist describes human remains)

The analytical table scientist_desc_humanremains provides further specifications about the fossils entered in the table humanremains. These descriptions provide additional information on published identifications of individual specimens. Each dataset is linked to the tables scientist and humanremains. This table will be used by experts who wish to develop dispersal scenarios for hominids including unpublished determinations. It will also be used in frame of "what if" scenarios by ROCEEH scientists.

**Analytical table: scientist_desc_humanremains**			
Attribute	Type	Key	Possible values
scientist_idscientist	Integer	Primary key/ Foreign key	[chosen from list].
humanremains_idlocality	String	Primary key/ Foreign key	[chosen from list] Blombos Cave, Aar 2
humanremains_idassemblage	Integer	Primary key/ Foreign key	[chosen from list]
humanremains_idhumanremains	Integer	Primary key/ Foreign key	[chosen from list]
genus	String		Australopithecus, Homo
species	String		afarensis, erectus, robustus, sapiens
comments	String		general comments
year	Integer		2011 [year stamp]
owner	String		Lawrence of Arabia
created	Date		[automatic date stamp]

The attribute **scientist_idscientist** creates a link to a dataset in the table scientist. Therefore, the name of the scientist who analyzed the human remains must already be entered in the table scientist.

The threefold attributes **humanremains_idlocality**, **humanremains_idassemblage** and **humanremains_idhumanremains** create a link to a dataset in the table humanremains.

The attribute **genus** contains the genus to which the described fossil is attributed in the reference to which the present dataset is linked. If the specimen cannot be identified to genus, the entry should be "indet". Possible entries include: Australopithecus, Homo, indet, etc.

The attribute **species** contains the species to which the described fossil is attributed. If the specimen cannot be identified to the species level, the entry should be "sp.". The absence of information is indicated by "n.c." (not communicated). Possible entries include: afarensis, sapiens, erectus, sp. or n.c.

The attribute **comments** contains general comments about the human remains.

The attribute **year** contains the year in which the data were entered and/or modified (current year stamp).

The attribute **owner** automatically indicates the last person who entered or changed the dataset.

The attribute **created** automatically indicates the date this dataset was originally entered. An additional attribute **mod_history** automatically records the modification history of a dataset including the names of users who made previous changes and the dates those changes were made.

Botany

Table: plantremains

The table plantremains characterizes the general fossil flora of an assemblage. A fossil flora can consist of two types of plant assemblages (pollen (i.e. plant microremains) or plant macroremains.) For each type of plant remain, one separate dataset should be entered, meaning that two assemblages should be created in the table assemblage, even if they were retrieved from the same level. Further details about taxonomic composition can be entered in the table paleoflora. If the assemblage is available in the NEOTOMA Paleoecology Database (https://www.neotomadb.org/) a respective note must be entered in comments.

**Primary table: plantremains**			
Attribute	Type	Key	Possible values
assemblage_idlocality	String	Primary key/ Foreign key	[chosen from list] Geelbek_Alice, Hohle Fels
assemblage_idassemblage	Integer	Primary key/ Foreign key	[chosen from list]
plant_remains	String	Primary key	pollen, plant macroremains
number_of_taxa	Integer		43
figure	String		[automatically generated]
comments	String		pollen counts published in Neotoma: GROBER94 Dataset ID 41357
owner	String		Wilhelm Tell
created	Date		[automatic date stamp]

The paired attributes **assemblage_idlocality** and **assemblage_idassemblage** define the locality and assemblage of the plant remains.

The attribute **plant_remains** indicates the type of fossil flora. Two types of plant remains may be entered, either pollen (plant microremains including other palynomorphs) or plant macroremains. For each type of plant remain, a separate dataset must be entered.

The attribute **number_of_taxa** indicates the number of taxa described in this type of fossil flora.

The attribute **figure** represents a link to a visualization of this type of the plant remain or fossil flora. The figure can be a plate or photo.

The attribute **comments** contains general comments about the nature of the plant remain. If the assemblage is available in the NEOTOMA Paleoecology Database (https://www.neotomadb.org/) a respective note must be entered here, e.g. pollen counts published in Neotoma: GROBER94 Dataset ID 41357. In this case one 'generic' assemblage is sufficient to lead the user to further details in NEOTOMA.

The attribute **owner** automatically indicates the last person who entered or changed the dataset.

The attribute **created** automatically indicates the date this dataset was originally entered. An additional attribute **mod_history** automatically records the modification history of a dataset including the names of users who made previous changes and the dates those changes were made.

Table: paleoflora

The table paleoflora specifies the plant taxa of a fossil flora. It describes the systematic rank of each taxon and gives information about abundance. Data available in the NEOTOMA Paleoecology Database (https://www.neotomadb.org/) will not be entered here, but are retrievable through the ROAD Map Module.

**Primary table: paleoflora**			
Attribute	Type	Key	Possible values
assemblage_idlocality	String	Primary key/ Foreign key	[chosen from list] Geelbek_Alice, Hohle Fels
assemblage_idassemblage	Integer	Primary key/ Foreign key	[chosen from list]
plantremains_plant_remains	String	Primary key/ Foreign key	[chosen from list] plant macroremains, pollen
plant_taxonomy_taxon	String	Primary key/ Foreign key	[chosen from list] Quercus ilex, Acer platanoides, Populus gokhtuniae
abundance	Integer		42
relative_abundance	Float		12.3
element	String		leaf, twig, bud, pollen, spore
figure	String		[automatically generated]
comments	String		general comments
owner	String		Xerxes of Persia
created	Date		[automatic date stamp]

The threefold attributes **assemblage_idlocality**, **assemblage_idassemblage** and **plantremains_plant_remains** define the types of plant remains found at a locality in a given assemblage.

The attribute **plant_taxonomy_taxon** presents the name of the fossil plant at any systematic rank as it was described or identified by the author. Information regarding nomenclature are stored in the look-up table plant_taxonomy.

The attribute **abundance** provides the number of specimens of the described taxon, if available.

The attribute **relative_abundance** indicates the relative abundance of the described taxon in percent, if available.

The attribute **element** specifies the element, part or organ of the fossil taxon identified.

- If the type of fossil flora is a plant macroremain, the element can be entered as: flower, bud, seed, cone, etc. Multiple entries are possible for this attribute because a plant taxon may be recorded by various parts of the plant in the same assemblage (e.g. leaves and fruits from the same species may be found.)

- If the type of flora is pollen (i.e. a plant microremain), the element can be entered as: pollen (angiosperms and gymnosperms), spore (ferns and fern allies, bryophytes), algae, etc. For microfloras multiple entries usually are not necessary, however these may occur in very rare cases.

The attribute **figure** graphically presents a link to a visualization of this type of fossil plant taxon. The figure can be a plate or photo.

The attribute **comments** contains general comments about the fossil plant taxon, especially notes of the original author with respect to state of preservation or abundance (rare, abundant, very abundant, etc.), if no absolute values are available.

The attribute **owner** automatically indicates the last person who entered or changed the dataset.

The attribute **created** automatically indicates the date this dataset was originally entered. An additional attribute **mod_history** automatically records the modification history of a dataset including the names of users who made previous changes and the dates those changes were made.

Table: plant_taxonomy

This look-up table provides the systematic rank of each plant taxon that occurs in the table plantremains.

**Look-up table: plant_taxonomy**			
Attribute	Type	Key	Possible values
taxon	String	Primary key	Quercus ilex, Acer platanoides, Populus gokhtuniae
family	String		Fagaceae
genus	String		Quercus
species	String		Quercus ilex L., Acer platanoides L., Populus alba L.
fossil_name	String		Acer platanoides L. fossilis, Populus gokhtuniae I. Gabr.
synonym_name	String		[enter synonym here]
comments	String		general comments
owner	String		Xerxes of Persia
created	Date		[automatic date stamp]

The attribute **taxon** presents the name of the fossil plant at any systematic rank as it was described or identified by the author. See notes regarding the attributes **fossil_name** and **synonym_name**.

The attribute **family** presents the family name of the described taxon. If the name of the family given by the author is not valid systematically, the attribute **family** indicates the *valid* name of the family.

The attribute **genus** presents the genus name of the described taxon. If the taxon is identified only to family level, the **genus** field should remain empty. If the name of the genus given by the author is not valid systematically, the attribute **genus** indicates the *valid* name of the genus.

The attribute **species** presents the *full and valid* species name including author's name (e.g. *Quercus ilex* L., *Sciadopitys verticillata* (Thunb.) Siebold & Zucc). If the taxon is identified only to family or genus level, the attribute **species** should remain empty. If the name of the species given by the author is not valid systematically, the attribute **species** indicates the *valid* name. The old name then has to be specified in the attribute **synonym_name**. If the name of the species given by the author is the name of a fossil, the attribute **species** represents the modern botanical name or the name of the *nearest living relative*. The full species name of the fossil plant taxon will be specified in the attribute **fossil_name**.

The attribute **fossil_name** provides the full species name of the fossil plant taxon including author's name, if the **taxon** is a fossil name.

The attribute **synonym_name** provides the *full, non valid* species name including author's name, if the **taxon** is not valid systematically.

The attribute **comments** contains general comments about the nature of the plant taxonomy.

The attribute **owner** automatically indicates the last person who entered or changed the dataset.

The attribute **created** automatically indicates the date this dataset was originally entered. An additional attribute **mod_history** automatically records the modification history of a dataset including the names of users who made previous changes and the dates those changes were made.

Climate & vegetation

Table: climate

The analytical table climate characterizes the climate parameters derived from the analysis of a fossil assemblage or taken from literature sources. Each dataset consists of the result of one analysis conducted with one reproducible method. In the future, as new methods are developed for climatic parameters other than those listed below, new attributes will be added to the list.

**Analytical table: climate**			
Attribute	Type	Key	Possible values
idclimate	Integer	Primary key	[automatically generated]
assemblage_idlocality	String	Primary key/ Foreign key	[chosen from list] Geelbek_Alice, Hohle Fels
assemblage_idassemblage	Integer	Primary key/ Foreign key	[chosen from list]
method	String		Coexistence Approach, CLAMP
taxa_analysed	Integer		43
mat_min	Float		13.5
mat_max	Float		13.8
mat_mean	Float		13.65
mat_dev	Float		0.15
cmt_min	Float		0.2
cmt_max	Float		4.1
cmt_mean	Float		2.15
cmt_dev	Float		1.95
wmt_min	Float		22.5
wmt_max	Float		24.3
wmt_mean	Float		23.4
wmt_dev	Float		0.9
map_min	Float		565
map_max	Float		1034
map_mean	Float		799.5
map_dev	Float		234.5
hmp_min	Float		102
hmp_max	Float		134
hmp_mean	Float		118
hmp_dev	Float		16
lmp_min	Float		10
lmp_max	Float		42
lmp_mean	Float		26
lmp_dev	Float		16
wmp_min	Float		49

wmp_max	Float		69
wmp_mean	Float		59
wmp_dev	Float		10
comments	String		general comments
owner	String		Yoyo Ma
created	Date		[automatic date stamp]

The attribute **idclimate** is an automatically generated, consecutive number of the dataset.

The paired attributes **assemblage_idlocality** and **assemblage_idassemblage** define the assemblage type at a given locality which was analyzed to determine climate.

The attribute **method** describes the name of the quantitative method used for this paleoclimatic analysis. Examples of methods may include Coexistence Approach, CLAMP, PDF (probability density function), or MAT (modern analog technique).

The attribute **taxa_analysed** indicates the number of taxa that form the basis of this analysis.

Mean Annual Temperature (MAT)

The attribute **mat_min** contains the minimum value of mean annual temperature given in degrees Celsius, if the climatic analysis results in a range. For example, the method Coexistence Approach results in a Coexistence Interval of 13.5-13.8 °C MAT. In this case, mat_min is 13.5.

The attribute **mat_max** contains the maximum value of mean annual temperature given in degrees Celsius, if the climatic analysis results in a range. For example, the method Coexistence Approach results in a Coexistence Interval of 13.5-13.8 °C MAT. In this case, mat_max is 13.8.

The attribute **mat_mean** contains the value of mean annual temperature given in degrees Celsius, if the climatic analysis results in a single value, and not a range.

The attribute **mat_dev** contains the value of standard deviation or error for **mat_mean** given in degrees Celsius, if available.

Mean Temperature of the Coldest Month (CMT)

The attribute **cmt_min** contains the minimum value of mean temperature of the coldest month given in degrees Celsius, if the climatic analysis results in a range. For example, see **mat_min**.

The attribute **cmt_max** contains the maximum value of mean temperature of the coldest month given in degrees Celsius, if the climatic analysis results in a range. For example, see **mat_max**.

The attribute **cmt_mean** contains the value of mean temperature of the coldest month given in degrees Celsius, if the climatic analysis results in a single value.

The attribute **cmt_dev** contains the value of standard deviation or error for **cmt_mean** given in degrees Celsius, if available.

Mean Temperature of the Warmest Month (WMT)

The attribute **wmt_min** contains the minimum value of mean temperature of the warmest month given in degrees Celsius, if the climatic analysis results in a range. For example, see **mat_min**.

The attribute **wmt_max** contains the maximum value of mean temperature of the warmest month given in degrees Celsius, if the climatic analysis results in a range. For example, see **mat_max**.

The attribute **wmt_mean** contains the value of mean temperature of the warmest month given in degrees Celsius, if the climatic analysis results in a single value.

The attribute **wmt_dev** contains the value of standard deviation or error for **wmt_mean** given in degrees Celsius, if available.

Mean Annual Precipitation (MAP)

The attribute **map_min** contains the minimum value of annual precipitation given in millimeters, if the climatic analysis results in a range. For example, see **mat_min**.

The attribute **map_max** contains the maximum value of annual precipitation given in millimeters, if the climatic analysis results in a range. For example, see **mat_max**.

The attribute **map_mean** contains the mean value of annual precipitation given in millimeters, if the climatic analysis results in a single value.

The attribute **map_dev** contains the value of standard deviation or error for **map_mean** given in millimeters, if available.

Highest Monthly Precipitation (HMP)

The attribute **hmp_min** contains the minimum value of highest monthly precipitation (precipitation of the wettest month) given in millimeters, if the climatic analysis results in a range. For example, see **mat_min**.

The attribute **hmp_max** contains the maximum value of highest monthly precipitation (precipitation of the wettest month) given in millimeters, if the climatic analysis results in a range. For example, see **mat_max**.

The attribute **hmp_mean** contains the mean value of highest monthly precipitation (precipitation of the wettest month) given in millimeters, if the climatic analysis results in a single value.

The attribute **hmp_dev** contains the value of standard deviation or error for **hmp_mean** given in millimeters, if available.

Lowest Monthly Precipitation (LMP)

The attribute **lmp_min** contains the minimum value of lowest monthly precipitation (precipitation of the driest month) given in millimeters, if the climatic analysis results in a range. For example, see **mat_min**.

The attribute **lmp_max** contains the maximum value of lowest monthly precipitation (precipitation of the driest month) given in millimeters, if the climatic analysis results in a range. For example, see **mat_max**.

The attribute **lmp_mean** contains the mean value of lowest monthly precipitation (precipitation of the driest month) given in millimeters, if the climatic analysis results in a single value.

The attribute **lmp_dev** contains the value of standard deviation or error for **lmp_mean** given in millimeters, if available.

Minimum Precipitation of the Warmest Month (WMP)

The attribute **wmp_min** contains the minimum value of monthly precipitation of the warmest month given in millimeters, if the climatic analysis results in a range. For example, see **mat_min**.

The attribute **wmp_max** contains the maximum value of annual precipitation given in millimeters, if the climatic analysis results in a range. For example, see **mat_max**.

The attribute **wmp_mean** contains the mean value of annual precipitation given in millimeters, if the climatic analysis results in a single value.

The attribute **wmp_dev** contains the value of standard deviation or error for **wmp_mean** given in millimeters, if available.

The attribute **comments** contains general comments about the calculation of climate.

The attribute **owner** automatically indicates the last person who entered or changed the dataset.

The attribute **created** automatically indicates the date this dataset was originally entered. An additional attribute **mod_history** automatically records the modification history of a dataset including the names of users who made previous changes and the dates those changes were made.

Table: scientist_desc_climate (scientist describes climate)

The linking table scientist_desc_climate links the scientist to the results of their climate analysis from a given year.

Linking table: scientist_desc_climate			
Attribute	Type	Key	Possible values
scientist_idscientist	Integer	Primary key/ Foreign key	[chosen from list]
climate_idclimate	Integer	Primary key/ Foreign key	[chosen from list]
year	Integer		2010 [year stamp]
comments	String		general comments
owner	String		Zenobia Jacobs
created	Date		[automatic date stamp]

The attribute **scientist_idscientist** identifies the person who conducted the climatic analysis, contributed the data, and is responsible for this dataset.

The attribute **climate_idclimate** identifies the dataset containing analytical results performed by the scientist.

The attribute **year** indicates the year of analysis.

The attribute **comments** contains general comments.

The attribute **owner** automatically indicates the last person who entered or changed the dataset.

The attribute **created** automatically indicates the date this dataset was originally entered. An additional attribute **mod_history** automatically records the modification history of a dataset including the names of users who made previous changes and the dates those changes were made.

Table: vegetation

The analytical table vegetation characterizes vegetation information derived from the analysis of a fossil flora. Each dataset consists of the result of one analysis conducted with one reproducible method. Such methods may include, for example: biomization based on the analysis of plant functional types (PFTs) (after Ni et al. 2010, or other modulations of the method); multivariate statistical methods; or more simple assessments of vegetation structure such as the ratio of arboreal to non-arboreal taxa (AP/NAP ratio). In the future, as new methods are developed for other vegetation parameters that are not listed below, we will add new attributes to the list.

**Analytical table: vegetation**			
Attribute	Type	Key	Possible values
idvegetation	Integer	Primary key	[automatically generated]
plantremains_idlocality	String	Foreign key	[chosen from list]
plantremains_idassemblage	Integer	Foreign key	[chosen from list]
plantremains_plant_remains	String	Foreign key	[chosen from list]
method	String		[chosen from list] Biomization after Ni et al. (2010) with 76 PFTs, Qualitative analysis
biome_classification	String		[chosen from list] Ni et al. (2010)
biome	String		[chosen from list] STEP_Temperate grassland
other_biomes	String		COMX_Cool-temperate evergreen needleleaf and mixed forest, TEDE_Temperate deciduous broadleaf forest
main_vegetation_unit	String		Artemisia-Chenopodiaceae steppe
other_vegetation_units	String		montane meadows, Juniper light forest
detailed_description	String		enter a detailed description of the vegetation (not flora)
ap_nap_ratio	Float		0.7
no_of_pfts	Integer		12
taxa_analysed	Integer		43
comments	String		general comments
owner	String		Aristotle
created	Date		[automatic date stamp]

The attribute **idvegetation** is an automatically generated, consecutive number of the dataset.

The threefold attributes **plantremains_idlocality**, **plantremains_idassemblage** and **plantremains_plant_remains** define the types of plant remains found in a given assemblage at a locality which were analyzed to determine vegetation.

The attribute **method** provides the name of the method used for this analysis. A dropdown-menu gives choices of widely used methods. Still, new methods can be entered here as needed. It is mandatory to fill in this attribute. Further details about the methodology may be entered in **comments** if necessary.

The attribute **biome_classification** specifies the classification system used by the author to describe the past vegetation represented by the given assemblage. It is selected from a fixed drop-down menu. Based on the classification chosen for the attribute **biome_classification**, a list of the respective biomes will be shown. This list is preselected from an internal look-up table for the attribute **biome**.

The attribute **biome** indicates the one biome identified as the main biome (or the most likely biome in a biomization analysis) represented at the given locality for the studied flora. According to the classification chosen for the attribute **biome_classification**, a fixed drop-down menu will restrict choices to the respective biomes for this respective classification.

If a biomization analysis results in more than one most likely biomes, then you should select the entry '2 equal best biomes' or '3 equal best biomes' and list them in the attribute **other_biomes**. If more than 3 biomes are given with the same likelihood, then chose '4 or more most likely biomes' and do not enter anything for the attribute **other_biome** (i.e. leave it empty).

If a biomization analysis results in two or three most likely biomes, then the attribute **other_biomes** lists those biomes separated by a comma. If more than 3 biomes are given with the same likelihood, then the attribute **other_biomes** is left empty.

The attribute **main_vegetation_unit** provides the main (dominant) vegetation unit for the studied flora (on a sub-biome level, i.e. formation, association, or other units), if available. Most often, this information will be derived from a descriptive qualitative analysis of the assemblage based on the experience of the author and summarizes the entry of **detailed_description**.

The attribute **other_vegetation_units** gives a list of other vegetation units (separated by a comma) which are less dominant, but also documented in the studied flora (on sub-biome level, i.e. formation, association, or other units), if available.

The attribute **detailed_description** provides a summary of the qualitative vegetation information available for the assemblage as given in the respective publication, if available. This attribute contains more detailed information than provided by the other attributes, but should be restricted to a description of the vegetation. Avoid entering lists of taxa names here.

The attribute **ap_nap_ratio** gives the ratio of the total number of arboreal pollen grains (AP) to that of the non-arboreal pollen grains (NAP), if the type of flora is pollen and the data are available.

The attribute **no_of_pfts** provides the number of plant functional types (PFTs) that appear in this fossil assemblage, if a PFT analysis was conducted and numbers are available.

The attribute **taxa_analysed** indicates the number of taxa that form the basis of this analysis.

The attribute **comments** contains general comments about the nature of the vegetation or details on the methodology used, if necessary.

The attribute **owner** automatically indicates the last person who entered or changed the dataset.

The attribute **created** automatically indicates the date this dataset was originally entered. An additional attribute **mod_history** automatically records the modification history of a dataset including the names of users who made previous changes and the dates those changes were made.

Table: scientist_desc_vegetation (scientist describes vegetation)

The linking table scientist_desc_vegetation links the scientist to the results of their vegetation analysis from a given year.

Linking table: scientist_desc_vegetation			
Attribute	Type	Key	Possible values
scientist_idscientist	Integer	Primary key/ Foreign key	[chosen from list]
vegetation_idvegetation	Integer	Primary key/ Foreign key	[chosen from list]
year	Integer		2010 [year stamp]
comments	String		general comments
owner	String		Bela Bartok
created	Date		[automatic date stamp]

The attribute **scientist_idscientist** identifies the person who conducted the climatic analysis, contributed the data and is responsible for this dataset.

The attribute **vegetation_idvegetation** identifies the dataset from the table vegetation.

The attribute **year** indicates the year of analysis.

The attribute **comments** contains general comments about the calculation of climate.

The attribute **owner** automatically indicates the last person who entered or changed the dataset.

The attribute **created** automatically indicates the date this dataset was originally entered. An additional attribute **mod_history** automatically records the modification history of a dataset including the names of users who made previous changes and the dates those changes were made.

Ecoprofiles

Table: ecoprofile

The analytical table ecoprofile collects ecological data on large mammal assemblages. A large mammal assemblage is defined as an inventory. Datasets consists of frequencies in which ecovariables are represented in a specific faunal inventory. Ecovariables are body mass, diet and locomotion type. The table ecoprofile serves to document the quantitative ecological evaluation of faunal assemblages.

**Analytical table: ecoprofile**			
Attribute	Type	Key	Possible values
assemblage_idlocality	String	Primary key/ Foreign key	[chosen from list] Geelbek_Stella, Hohle Fels
assemblage_idassemblage	Integer	Primary key/ Foreign key	[chosen from list]
idecoprofile	Integer	Primary key	[automatically generated]
bodymass_1a	Float		0.735
bodymass_1b	Float		0.735
bodymass_1c	Float		0.735
bodymass_2a	Float		0.735
bodymass_2b	Float		0.735
bodymass_2c	Float		0.735
bodymass_3a	Float		0.735
bodymass_3b	Float		0.735
bodymass_3c	Float		0.735
bodymass_4a	Float		0.735
bodymass_4b	Float		0.735
bodymass_4c	Float		0.735
bodymass_5a	Float		0.735
bodymass_5b	Float		0.735
bodymass_5c	Float		0.735
diet_car	Float		0.326
diet_cax	Float		0.326
diet_cin	Float		0.326
diet_cpi	Float		0.326
diet_her	Float		0.326
diet_hfr	Float		0.326
diet_hxb	Float		0.326
diet_hmb	Float		0.326
diet_hmg	Float		0.326
diet_hxg	Float		0.326
diet_omi	Float		0.326

locomotion_aer	Float		0.455
locomotion_amp	Float		0.455
locomotion_arb	Float		0.455
locomotion_fos	Float		0.455
locomotion_gra	Float		0.455
locomotion_sar	Float		0.455
locomotion_swi	Float		0.455
locomotion_ubi	Float		0.455
locomotion_ung1	Float		0.455
locomotion_ung2	Float		0.455
locomotion_ung3	Float		0.455
locomotion_ung4	Float		0.455
profile_refers_to	String		MNI, NISP, Species richness
year	Integer		2010 [year stamp]
comments	String		general comments
owner	String		Xerxes of Persia
created	Date		[automatic date stamp]

The paired attributes **assemblage_idlocality** and **assemblage_idassemblage** define the locality and assemblage of the ecoprofile.

The attribute **idecoprofile** is an automatically generated, consecutive number of the dataset.

Fifteen attributes for body mass **bodymass_1a**, **bodymass_1b**, **bodymass_1c**, **bodymass_2a**, **bodymass_2b**, **bodymass_2c**, **bodymass_3a**, **bodymass_3b**, **bodymass_3c**, **bodymass_4a**, **bodymass_4b**, **bodymass_4c**, **bodymass_5a**, **bodymass_5b** and **bodymass_5c** indicate the frequencies at which defined body mass classes are represented in a given assemblage. Ranges of body mass classes are defined as follows:

Ranges of body mass classes in kg	a	b	c
1	0.1–0.19	0.2–0.49	0.5–0.99
2	1.0–1.9	2.0–4.9	5.0–9.9
3	10–19	20–49	50–99
4	100–199	200–499	500–999
5	1,000–1,999	2,000–4,999	5,000–10,000

Frequencies are based on body mass estimates. Absolute body mass estimates are assigned to a particular body mass class and frequencies in assemblages are calculated.

Eleven attributes for diet **diet_car**, **diet_cax**, **diet_cin**, **diet_cpi**, **diet_her**, **diet_hfr**, **diet_hxb**, **diet_hmb**, **diet_hmg**, **diet_hxg** and **diet_omi** indicate frequencies at which defined dietary categories are represented in a given assemblage. The dietary categories are defined as follows:

Diet class	Definition	Diet class	Definition
CAR	General carnivore	HXB*	Specialized browser
CAX	Specialized (hyper-) carnivore	HMB*	Mixed feeder
CIN	Insectivore	HMG*	Less selective grazer
CPI	Piscivore	HXG*	Specialized grazer
HER	General herbivore	OMI	Omnivore
HFR	Frugivore		

* The diet classes HXB, HMB, HMG and HXG may be quantitatively distinguished on the basis of mesowear analyses or other quantitative approaches working with a similar classification.

Twelve attributes for locomotion: **locomotion_aer**, **locomotion_ung1**, **locomotion_ung2**, **locomotion_ung3**, **locomotion_ung4**, **locomotion_ubi**, **locomotion_gra**, **locomotion_sar**, **locomotion_arb**, **locomotion_swi**, **locomotion_amp** and **locomotion_fos** describe the frequencies in which defined locomotion classes are represented in a given assemblage. The locomotion classes are defined as follows:

Locomotion class	Definition	Locomotion class	Definition
AER	Aerial, flying	GRA	Graviped
UNG1	Ungulate type 1*	SAR	Semiarboreal
UNG2	Ungulate type 2*	ARB	Arboreal, climbing adaptations
UNG3	Ungulate type 3*	SWI	Specialized swimmer
UNG4	Ungulate type 4*	AMP	Amphibic
UBI	Ubiquitous, not specialized	FOS	Fossorial, digging

* Ungulate types [automatically generated]and 4 may be quantitatively assigned on the basis of a morphometric analysis of postcranial elements or other quantitative methods working with a similar classification.

The attribute **profile_refers_to** indicates the type of elements on which the frequencies in the ecological analysis are based. The quantification is based either on the minimum number of individuals (MNI) in the assemblage, the number of identified specimens (NISP) or the number of species (species richness) represented in a given assemblage.

The attribute **year** states the year in which the ecological analysis of the assemblage was completed.

The attribute **comments** contains general comments about the ecoprofile of the assemblage.

The attribute **owner** automatically indicates the last person who entered or changed the dataset.

The attribute **created** automatically indicates the date this dataset was originally entered. An additional attribute **mod_history** automatically records the modification history of a dataset including the names of users who made previous changes and the dates those changes were made.

Table: scientist_desc_ecoprofile (scientist describes ecoprofile)

The linking table scientist_desc_ecoprofile links the scientist who analyzed an ecoprofile with the results of the ecoprofile analysis of one or more assemblages from a specific locality.

**Linking table: scientist_desc_ecoprofile**			
Attribute	Type	Key	Possible values
scientist_idscientist	Integer	Primary key/ Foreign key	[chosen from list]
ecoprofile_idlocality	String	Primary key/ Foreign key	[chosen from list] Geelbek_Alice, Hohle Fels
ecoprofile_idassemblage	Integer	Primary key/ Foreign key	[chosen from list]
ecoprofile_idecoprofile	Integer	Primary key/ Foreign key	[chosen from list]
ecoprofile_class	String		Fynbos, TBSh
year	Integer		2010 [year stamp]
comments	String		general comments
owner	String		Ludwig van Beethoven
created	Date		[automatic date stamp]

The attribute **scientist_idscientist** identifies the person who conducted the ecoprofile analysis, contributed the data, and is responsible for this dataset.

The threefold attributes: **ecoprofile_idlocality**, **ecoprofile_idassemblage** and **ecoprofile_idecoprofile** specifies an ecological profile for a specific assemblage from a locality.

The attribute **ecoprofile_class** describes the specific ecological setting inferred from ecoprofiles. The classification systems differ by region and are externally defined. Possible values are for instance fynbos, a biome valid in South Africa, or TBSh, a quantitatively defined type of tropical shrubland valid in African and Asian tropical systems.

The attribute **year** states the year in which the ecological interpretation of a given assemblage was completed.

The attribute **comments** contains general comments about the ecoprofile of the assemblage.

The attribute **owner** automatically indicates the last person who entered or changed the dataset.

The attribute **created** automatically indicates the date this dataset was originally entered. An additional attribute **mod_history** automatically records the modification history of a dataset including the names of users who made previous changes and the dates those changes were made.

Fauna

Table: taxonomical_classification

The look-up table taxonomical_classification lists the classification of fauna based on its Linnaean taxonomy. The table starts from the genus level and moves taxonomically upwards through infratribe, subtribe, tribe, subfamily, family, superfamily, parvorder, infraorder, suborder, order, superorder to class.

**Look-up table: taxonomical_classification**			
Attribute	Type	Key	Possible values
idtaxonomical_classification	Integer	Primary key	[automatically generated]
genus	String		Panthera, Bos, Cervid
infratribe	String		Rhinoceroti
subtribe	String		Rhinocerotina, Monachina, Gerbillurina
tribe	String		Leithini, Pongini, Canini
subfamily	String		Lutrinae, Caninae, Cervinae
family	String		Canidae, Bovidae, Mustelidae
superfamily	String		Muroidea, Bovoidea
parvorder	String		Anthropoidea, Mustelida, Ursida
infraorder	String		Haplorhini, Arctoidea, Cynoidea
suborder	String		Ruminantia, Fliformia, Caniformia
order_	String		Rodentia, Primates, Carnivora
superorder	String		Afrotheria, Laurasiatheria
class	String		Mammalia, Reptilia, Aves
comments	String		general comments
owner	String		Michael Fassbender
created	Date		[automatic date stamp]

The attribute **idtaxonomical_classification** is an automatically generated, consecutive number of the dataset that will be subsequently linked to the table paleofauna.

The attribute **genus** contains the taxonomic genus for each taxon of fauna.

The attribute **infratribe** contains the taxonomical infratribe of each genus. If the infratribe is unknown this attribute remains empty.

The attribute **subtribe** contains the taxonomical subtribe of each genus. If the subtribe is unknown this attribute remains empty.

The attribute **tribe** contains the taxonomical tribe of each genus. If the tribe is unknown this attribute remains empty.

The attribute **subfamily** contains the taxonomical subfamily of the genus. If the subfamily is unknown this attribute remains empty.

The attribute **family** contains the taxonomical family of each genus. If the family is unknown this attribute remains empty.

The attribute **superfamily** contains the taxonomical superfamily of the genus. If the superfamily is unknown this attribute remains empty.

The attribute **parvorder** contains the taxonomical parvorder of the genus. If the parvorder is unknown this attribute remains empty.

The attribute **infraorder** contains the taxonomical infraorder of the genus. If the infraorder is unknown this attribute remains empty.

The attribute **suborder** contains the taxonomical suborder of the genus. If the suborder is unknown this attribute remains empty.

The attribute **order_** contains the taxonomical order of the genus. If the order is unknown this attribute remains empty. (The underscore after order is required because otherwise order will be understood as a computer command.)

The attribute **superorder** contains the taxonomical superorder of the genus. If the superorder is unknown this attribute remains empty.

The attribute **class** contains the taxonomical class name of each genus.

The attribute **comments** contains general comments about the nature of the taxonomical classification.

The attribute **owner** automatically indicates the last person who entered or changed the dataset.

The attribute **created** automatically indicates the date this dataset was originally entered. An additional attribute **mod_history** automatically records the modification history of a dataset including the names of users who made previous changes and the dates those changes were made.

Table: paleofauna

The primary table paleofauna contains information about the taxonomic classification of the faunal remains in an assemblage. For each taxon occurring in an assemblage, the number of specimens and the composition of age and sex can be entered. The minimum number of individuals (MNI) can additionally be added to the table publication_desc_paleofauna.

**Primary table: paleofauna**			
Attribute	Type	Key	Possible values
assemblage_idlocality	String	Primary key/ Foreign key	[chosen from list]
assemblage_idassemblage	Integer	Primary key/ Foreign key	[chosen from list]
taxonomical_classification_id_t_c	Integer	Primary key/ Foreign key	[chosen from list]
species	String	Primary key	capensis, oreotragus, lupus
nisp_anat	Integer		-1, 0, 734, 789, 798
infant	Integer		0, 1, 27, 398
juvenile	Integer		0, 1, 27, 398
subadult	Integer		0, 1, 27, 398
adult	Integer		0, 1, 27, 398
mature	Integer		0, 1, 27, 398
indet_age_distribution	Integer		0, 1, 27, 398
male	Integer		0, 1, 27, 398
female	Integer		0, 1, 27, 398
indet_sex_distribution	Integer		0, 1, 27, 398
figure	String		[automatically generated]
comments	String		general comments
owner	String		Nelson Mandela
created	Date		[automatic date stamp]

The paired attributes **assemblage_idlocality** and **assemblage_idassemblage** define the locality and assemblage of the faunal remain.

The attribute **taxonomical_classification_id_t_c** is the number of the dataset that identifies the taxonomic classification. This attribute links the genus and its higher taxonomic classification from the table **taxonomical_classification** with the species entered in this table.

The attribute **species** represents the Linnaean classification of the faunal remain. Note that genus (and its higher taxonomic classification levels) will be selected from the table taxonomical_classification. The following conventions should be used:

- (name) – use parentheses to indicate an alternate classification
- / – indicates dual genera (e.g. Bos/Bison, Ovis/Capra)
- ? – indicates uncertainty in the determination
- aff. – with affinity to
- cf. – compare with
- gen. – genus
- gr. – group
- indet. – indeterminate
- sp. – species (singular), when a taxon cannot be specified
- spp. – species (plural), when two or more taxa cannot be specified
- sp. nov. – new species
- ssp. – subspecies
- s.s. – *sensu strictu*, in the strict sense
- var. – variety
- vel – or

The attribute **nisp_anat** (number of identified specimens_anatomical) contains the number of anatomically definable elements in an assemblage. The elements need not be definable taxonomically and the laterality of elements need not be given.

The attribute **infant** contains the number of infant individuals of each taxon. The number may be 0. If age determination is not available, "-1" should be entered.

The attribute **juvenile** contains the number of juvenile individuals of each taxon. The number may be 0. If age determination is not available, "-1" should be entered.

The attribute **subadult** contains the number of subadult individuals of each taxon. The number may be 0. If age determination is not available, "-1" should be entered.

The attribute **adult** contains the number of adult individuals of each taxon. The number may be 0. If age determination is not available, "-1" should be entered.

The attribute **mature** contains the number of matured individuals of each taxon. The number may be 0. If age determination is not available, "-1" should be entered.

The attribute **indet_age_distribution** contains the number of individuals with an undetermined age. The number may be 0. If age determination is not available, "-1" should be entered.

The attribute **male** (male) contains the number of male individuals of each taxon. The number may be 0. If no sex determination is available, "-1" should be entered.

The attribute **female** (female) contains the number of female individuals of each taxon. The number may be 0. If no sex determination is available, "-1" should be entered.

The attribute **indet_sex_distribution** contains the number of individuals with an indeterminate sex. The number may be 0. If no sex determination is available, "-1" should be entered.

The attribute **figure** represents a link to a visualization of this type of faunal remain. The figure can be a plate or photo.

The attribute **comments** contains general comments about the nature of the faunal remain.

The attribute **owner** automatically indicates the last person who entered or changed the dataset.

The attribute **created** automatically indicates the date this dataset was originally entered. An additional attribute **mod_history** automatically records the modification history of a dataset including the names of users who made previous changes and the dates those changes were made.

Table: animalremains

The primary table animalremains contains data on the anatomical composition of the animal remains. This table also includes data about the frequency of different skeletal elements and also different types of taphonomic modification (e.g. anthropogenic, biogenic, geogenic).

**Primary table: animalremains**			
Attribute	Type	Key	Possible values
assemblage_idlocality	String	Primary key/ Foreign key	[chosen from list] Geelbek_Stella, Hohle Fels
assemblage_idassemblage	Integer	Primary key/ Foreign key	[chosen from list]
no_cranial	Integer		-1, 0, 27, 30, 45, 987
no_dental_total	Integer		-1, 0, 17, 23, 765
no_dental_maxillary	Integer		-1, 0, 17, 23, 876
no_dental_mandibular	Integer		-1, 0, 17, 23, 890
no_postcranial	Integer		-1, 0, 17, 23, 765
anthropogenic_modifications	String		impact, cut, burn, saw
biogenic_modifications	String		bite, chew, gnaw, wasp, insect bite, trampling, root
geogenic_modifications	String		dry cracks, exfoliated, etch, sandblast, polish, rounded, encrusted, burn
taphonomic_selection	Integer		4, 3, 2.5, 2, 1, 0
figure	String		[automatically generated]
comments	String		general comments
owner	String		Manuel Noriega
created	Date		[automatic date stamp]

The paired attributes **assemblage_idlocality** and **assemblage_idassemblage** define the locality and assemblage for the type of animal remain.

The attribute **no_cranial** contains the number of cranial elements in an assemblage including mandible fragments.

The attribute **no_dental_total** contains the total number of teeth in an assemblage.

The attribute **no_dental_maxillary** contains the number of maxillary teeth in an assemblage.

The attribute **no_dental_mandibular** contains the number of mandibular teeth in an assemblage.

The attribute **no_postcranial** contains the number of postcranial elements in an assemblage.

The attribute **anthropogenic_modifications** contains a list of man-made marks which can be observed on faunal elements. High inter-study variability exists among researchers, and this affects how modifications are examined, described and interpreted. The list below combines terms that describe these traces and also their interpretations. While we have tried to select frequent categories of modifications, the list is not exhaustive. New modifications may be added to the list only after discussion with the advisors, and multiple entries are allowed. Values for modifications are selected from a drop-down list:

- burn: elements show clear traces of fire (e.g. gray, black or white color, craquelure or pot-lid fractures)
- butchering indet: authors' interpret butchering, but detailed descriptions of the anthropogenic modifications are not available; often used when several types of marks related to butchering are identified (e.g. chop, cut, disarticulation, filleting, skinning)
- chop: elements show coarse traces of chopping
- cut: elements show definite or probable cut marks
- disarticulation: includes arrachement, breakthrough and peeling; arrachement is the loss of cortical bone tissue related to disarticulation; breakthrough occurs on the surface of bones due to manipulation by torsion, wrenching or overextension; peeling is defined as a roughened surface with parallel grooves and a fibrous texture
- flake: products of bone marrow acquisition or butchering; flakes can be preforms for tools, tools or debitage from tool production. Flakes should be entered as anthropogenic modifications in the table animalremains if no information about use wear or percussion directions is given. However, if use wear or percussion from the transverse or inner part of the bone is visible, or if the authors provide good arguments, flakes should instead be entered in the table organic_tools.
- fresh break: elements show fresh, spiral or green breaks; related to bone marrow or raw material acquisition; oblique angles and smooth edges indicates fresh bone breakage
- human tooth marks: elements show crenulated edges, small pits related to broken edges, longitudinal fissures and peeling; often associated with birds or small mammals
- impact: elements show impact, percussion or conchoidal fractures
- meat removal: elements show traces related to acquisition of meat from a carcass, for example defleshing or filleting
- notch: element shows signs of intentional notching or denticulation
- peeling: element shows roughened surface with parallel grooves and a fibrous texture; can also be included under disarticulation
- polish: elements appear polished or smoothed through repeated use or abrasion

- retoucher: elements show peck marks and hammer damage from being used as a retoucher. However, consider entering the object in the table organic_tools if the evidence for retoucher is strong.
- sawing: elements show signs of being sawed
- skinning: elements show traces related to acquisition of skins or fur from a carcass
- striae: elements are scratched, striated or scraped
- unknown: no detailed information about the type of modification is known

The attribute **biogenic_modifications** contains a list of alterations caused by non-human agents that can be observed on faunal elements. High inter-study variability exists among researchers, and this affects how modifications are examined, described and interpreted. The list below combines terms that describe these traces and also their interpretations. While we have tried to select frequent categories of modifications, the list is not exhaustive. New modifications may be added to the list only after discussion with the advisors, and multiple entries are allowed. Values for modifications are selected from a drop-down list:

- carnivore damage: elements show signs of biting, chewing, gnawing or puncture marks caused by carnivores
- gastric: elements show signs such as etching and polish caused by digestion
- insect: elements show marks caused by social insects (e.g. mud wasp boring, termite channeling)
- raptor damage: elements show signs of impact by raptors (e.g. pecking)
- rodent gnawing: elements show signs of gnaw marks by rodents including porcupines
- root etching: elements etched by plant roots
- trampling: elements show signs of trampling by animals
- unknown: no detailed information about the type of modification is known

The attribute **geogenic_modifications** contains a list of changes caused by geological or other natural/environmental factors) that can be observed on elements. High inter-study variability exists among researchers, and this affects how modifications are examined, described and interpreted. The list below combines terms that describe these traces and also their interpretations. While we have tried to select frequent categories of modifications, the list is not exhaustive. New modifications may be added to the list only after discussion with the advisors, and multiple entries are allowed. Values for modifications are selected from a drop-down list:

- abrasion: elements appear abraded by sediments
- chemical weathering: elements show pitting and mineral staining
- dry: elements shows drying or desiccation cracks
- encrusted: elements are at least partially covered by a carbonate crust
- mineralized: elements show evidence for mineralization or fossilization

- natural break: elements are broken geometrically, parallel or perpendicular to the bone
- smooth: elements appear smoothed, but the cause is not known
- water-worn: elements appear rounded by water
- weathering: elements are weathered; if the weathering stage (Behrensmeyer 1978) is known, enter the stage, or a range of stages, in the **comments**.
- unknown: no detailed information about the type of modification is known

The attribute **taphonomic_selection** is a parameter ranging from 0 to 4 that assesses the amount of ecological distortion caused by biostratinomic or taphonomic selection. The amount of distortion is indicated by a number:

- 4: if the inventory is a primary deposit and has not been transported.
- 3: if the inventory has only been transported and sorted physically (e.g. transported in water or non-biological agents). This value is not used if the inventory has been transported by living organisms.
- 2.5: indicates transportation by less selective organisms (e.g. hyenas).
- 2: indicates transportation by more selective organisms (e.g. porcupines).
- 1: indicates that portions of the assemblage have been redeposited (partially secondary deposit, inventory ecologically mixed).
- 0: if the inventory is an anthropogenic selection (e.g. hunting remains), due to possible discordance between the original and the altered condition, these assemblages are not considered to have an ecological value.

The attribute **figure** represents a link to a visualization of this type of animal remain. The figure can be a plate or photo.

The attribute **comments** contains general comments about the nature of the animal remain.

The attribute **owner** automatically indicates the last person who entered or changed the dataset.

The attribute **created** automatically indicates the date this dataset was originally entered. An additional attribute **mod_history** automatically records the modification history of a dataset including the names of users who made previous changes and the dates those changes were made.

Postal address, people & organizations

Table: postal_address

The primary table postal_address specifies basic data about where a scientist or organization is located.

Primary table: postal_address			
Attribute	Type	Key	Possible values
idpostal_address	Integer	Primary key	[automatically generated]
geopolitical_units_country_idgeopolitical_units_country	String	Foreign key	[chosen from list]
geopolitical_units_province_idgeopolitical_units_province	String	Foreign key	[chosen from list]
city	String		Phoenix, Nairobi, Delhi
comments	String		general comments
owner	String		Donald F. Trump
created	Date		[automatic date stamp]

The attribute **idpostal_address** is the automatically generated, consecutive number of the dataset.

The attribute **geopolitical_units_country_id geopolitical_units_country** is an automatically generated, consecutive number of the dataset which is linked to the table geopolitical_units_country. It describes the country in which a scientist or organization is located.

The attribute **geopolitical_units_province_id geopolitical_units_ province** is an automatically generated, consecutive number of the dataset which is linked to the table geopolitical_units_province. It describes the province in which a scientist or organization is located.

The attribute **city** specifies the city in which the scientist or organization is located (e.g. Boston, Addis Ababa, Jakarta).

The attribute **comments** contains general information about the postal address.

The attribute **owner** automatically indicates the last person who entered or changed the dataset.

The attribute **created** automatically indicates the date this dataset was originally entered. An additional attribute **mod_history** automatically records the modification history of a dataset including the names of users who made previous changes and the dates those changes were made.

Table: scientist

The primary table scientist contains the name and contact information of the scientist who analyzed materials from a locality. Specifically, the scientist may have conducted analyses related to the tables humansremains, climate, vegetation, ecoprofile. If more than one scientist is involved, each one must be entered into a separate dataset. A scientist cannot be an organization.

**Primary table: scientist**			
Attribute	Type	Key	Possible values
idscientist	Integer	Primary key	[automatically generated]
name	String		C. Hertler, A. Bruch, M.N. Haidle
email	String		Cambridge, England
url	String		general comments
postal_address_idpostal_address	String	Foreign key	[chosen from list]
comments	String		general comments
owner	String		Friederich der Grosse
created	Date		[automatic date stamp]

The attribute **idscientist** specifies a scientist and is an automatically generated, consecutive number of the dataset. Additional persons must be entered in new datasets.

The attribute **name** contains the name of the scientist.

The attribute **email** specifies the e-mail address of the scientist and is presented in standard format (e.g. email.address@institute.country).

The attribute **url** specifies the Uniform Resource Locator of the scientist and should be presented in standard format (e.g. http://www.institute.department.page).

The attribute **postal_address_idpostal_address** links the location of the scientist through the table postal_address.

The attribute **comments** contains general comments about the scientist.

The attribute **owner** automatically indicates the last person who entered or changed the dataset.

The attribute **created** automatically indicates the date this dataset was originally entered. An additional attribute **mod_history** automatically records the modification history of a dataset including the names of users who made previous changes and the dates those changes were made.

Table: organization

The primary table organization specifies the organization which curates the assemblage. If more than one organization is involved, each one must be entered as a separate dataset. An organization cannot be a scientist.

**Primary table: organization**			
Attribute	Type	Key	Possible values
idorganization	Integer	Primary key	[automatically generated]
organization	String		Cambridge University
email	String		joe.schmoe@uni-southpole.aq
url	String		http://www.roceeh.net
postal_address_idpostal_address	String	Foreign key	[chosen from list]
comments	String		general comments
owner	String		Vladimir Nabokov
created	Date		[automatic date stamp]

The attribute **idorganization** is an automatically generated, consecutive number of the dataset.

The attribute **organization** specifies the organization which curates the assemblage.

The attribute **email** specifies the e-mail address of the organization and is presented in standard format (e.g. email.address@institute.country).

The attribute **url** specifies the Uniform Resource Locator of the organization and should be presented in standard format (e.g. http://www.institute.department.page).

The attribute **postal_address_idpostal_address** links the location of the organization through the table postal_address.

The attribute **comments** contains general comments about the organization.

The attribute **owner** automatically indicates the last person who entered or changed the dataset.

The attribute **created** automatically indicates the date this dataset was originally entered. An additional attribute **mod_history** automatically records the modification history of a dataset including the names of users who made previous changes and the dates those changes were made.

Table: organization_preserves_assemblage

The linking table organization_preserves_assemblage links the organization which curates an assemblage with the actual assemblage. If more than one organization is involved, each organization must be linked to the assemblage.

**Linking table: organization_preserves_assemblage**			
Attribute	Type	Key	Possible values
assemblage_idlocality	Integer	Primary key/ Foreign key	[chosen from list]
assemblage_idassemblage	Integer	Primary key/ Foreign key	[chosen from list]
organization_idorganization	Integer	Primary key/ Foreign key	[chosen from list]
owner	String		Alan Turing
created	Date		[automatic date stamp]

The paired attributes **assemblage_idlocality** and **assemblage_idassemblage** link the locality and assemblage to the organization.

The attribute **organization_idorganization** links the organization to the assemblage.

The attribute **owner** automatically indicates the last person who entered or changed the dataset.

The attribute **created** automatically indicates the date this dataset was originally entered. An additional attribute **mod_history** automatically records the modification history of a dataset including the names of users who made previous changes and the dates those changes were made.

Geographical information

Table: geodata (geographic data)

The primary table geodata describes each individual geographic file contained in ROAD. This metadata table should be filled out for each uploaded file. The table geodata contains information about the thematic and spatial coverage of the data. It also contains information about the spatial reference and file format, as well as the source of the data and modifications to these data.

**Primary table: geodata**			
Attribute	Type	Key	Possible values
idgeodata	Integer	Primary key	[automatically generated]
data_type	String		Vector, geoTIFF, PNG, time series
spatial_scale	String		1:25000, 90x90m, unknown
map_name	String		unique title
srid	Integer		4326, 32737
data_source	String		scanned analog map, digital aerial photo
creator	String		NCDC's Geodata Portal http://gis.ncdc.noaa.gov/
modifications_preprocessing	String		none, DEM pre-processed
coverage_keywords	String		vegetation, ASTER, L-Band, roughness
coverage_continental	String		Africa, Asia, Europe, none
coverage_national	String		Germany, South Africa, Tanzania, none
bbox	box2d		[automatically generated]
the_geom	String		[automatically generated]
comments	String		general comments
owner	String		Knut der Eisbär
created	Date		[automatic date stamp]

The attribute **idgeodata** is an automatically generated, consecutive number of the dataset.

The attribute **data_type** describes the format of the data. Vector data can be stored as shape files (shp). Raster data are handled as images, and simple tables can be in csv or txt format.

The attribute **spatial_scale** describes the spatial resolution of geodata for which scale the original data were generated (e.g. a topographic map with a scale of 1:10,000 or a geologic map with a scale of 1:25,000). If raster data are described, the spatial scale refers to the pixel

length. For example, a raster dataset such as an SRTM-DEM with 90 m pixel length has a spatial resolution of 90 m.

The attribute **map_name** describes the file name of each piece of data (e.g. shapefiles, raster images such as png or tif). The naming convention is predefined as [continent_country_content] (e.g. africa_namibia_admin; africa_vegetation_lgm).

The attribute **srid** contains the European Petroleum Survey Group (EPSG) Spatial Reference ID (SRID). SRID is a unique value used to unambiguously identify projected, unprojected and local spatial coordinate system definitions. These coordinate systems form the heart of all GIS applications. Virtually all major spatial vendors have created their own SRID implementation or refer to those of an authority, such as the EPSG. As of 2005 the EPSG SRID values are now maintained by the International Association of Oil & Gas Producers Surveying & Positioning Committee. SRIDs are the primary key for the Open Geospatial Consortium spatial_ref_sys metadata table. The SRID must be determined for the geodata that are uploaded. In order to attribute the right SRID you must know the datum and the coordinate system. The individual SRID or EPSG number can be found at: http://spatialreference.org/ref/epsg/.

The attribute **data_source** describes the source of the data if original digital data are utilized, or the source data from which the geodata were derived (e.g. DEM raster with 5 m resolution derived from official topographic map 1:10,000 Sheet Tübingen, Baden Württemberg, 1998 paper format).

The attribute **creator** is the person or institution that authored the data (e.g. geologisches Landesamt NRW, Manfred Muster).

The attribute **modifications_preprocessing** describes the creation or preprocessing procedure which are performed before the data were uploaded into ROAD (transformation from SRTM image to GRID, fill sink with ARCGIS 9.3, filtering with 3 x 3 simple filter after Conrad (2007), conversion from GRID to PNG).

The attribute **coverage_keywords** describes the thematic specification of the data. A comma-separated list contains one or several these keywords: LiDAR, fallow land, ASTER, X-Band, Roughness, Vegetation, and so on. Please orient yourself to existing entries when creating a new one. More than one entry is allowed, each separated by a comma.

The attribute **coverage_continental** denotes the spatial coverage of the project. It can be different from the actual data created. Our convention is: none, Europe, Asia, Africa, North America, South America, Antarctica, Australia or World.

The attribute **coverage_national** indicates the individual sovereign state, as named by the United Nations. A state should be entered with its customary name in English, e.g. Tanzania and NOT United Republic of Tanzania, United Kingdom and NOT England, China and NOT People's Republic of China. If it is a methodical work, enter "none".

The attribute **bbox** describes the bounding box corners (lower left, upper right) in the Spatial Reference System units of the data. The coordinates (bbox=minx,miny,maxx,maxy) are generated automatically.

In the attribute **the_geom** the geometry of the respective data is stored. It is generated automatically.

The attribute **comments** contains general comments about the geodata.

The attribute **owner** automatically indicates the last person who entered or changed the dataset.

The attribute **created** automatically indicates the date this dataset was originally entered. An additional attribute **mod_history** automatically records the modification history of a dataset including the names of users who made previous changes and the dates those changes were made.

Table: locality_geodata (locality has geographic data)

This table links a locality with the geographical information that describes it.

Linking table: locality_geodata			
Attribute	Type	Key	Possible values
geodata_idgeodata	Integer	Primary key/<br>Foreign key	[chosen from list]
locality_idlocality	String	Primary key/<br>Foreign key	[chosen from list]<br>Geelbek_Alice, Hohle Fels
owner	String		Vitali Klitschko
created	Date		[automatic date stamp]

The attribute **geodata_idgeodata** creates a link to a dataset in the table geodata.

The attribute **locality_idlocality** creates a link to a dataset in the table locality.

The attribute **owner** automatically indicates the last person who entered or changed the dataset.

The attribute **created** automatically indicates the date this dataset was originally entered. An additional attribute **mod_history** automatically records the modification history of a dataset including the names of users who made previous changes and the dates those changes were made.

Source data

Table: publication_source

The primary table publication source provides information about a journal, book, database or web article. A publication source (e.g. ISSN, ISBN, DOI) can be identified through its code number and title. A publication source usually has a publisher and one or more editors. Some publication sources are identified through URLs.

Primary table: publication_source			
Attribute	Type	Key	Possible values
id_source	Integer	Primary key	[automatically generated]
id_source_description	String		ISSN, ISBN, DOI
idnumber	String		3-934054-20-X, 0724-8679
title	String		Nature, Bild der Wissenschaft
publisher	String		Oldenburg Verlag
publication_place	String		Munich
editor	String		Kemper, H., Eickler, A., Becker, B.
url	String		http://www.neotomadb.org/
comments	String		general comments
access_date	Date		28 March 2022
owner	String		Christiane Amanpour
created	Date		[automatic date stamp]

The attribute **id_source** is an automatically generated, consecutive number of the dataset.

The attribute **id_source_description** can be selected from one of the following strings:

- **ISSN:** International Standard Serial Number is a unique identifying number used by journals and publications.
- **ISBN:** International Standard Book Number is a unique 10-13 digit identifier to denote books and other individual non-serial publications (e.g. multimedia products or software).

The attribute **idnumber** contains the identifying number of the ISBN or ISSN.

The attribute **title** is the name of the publication source and represents the title of a book, journal or database.

The attribute **publisher** contains the name of the publisher. Do not use abbreviations.

The attribute **publication_place** contains the location of the publisher. Only include the first city of publication. Do not include additional information such as province, state or country. If the **publication_place** is not known, leave this field blank.

The attribute **editor** contains the name(s) of the person(s) who edited or prepared a scientific text for publication. Names should be entered using the complete last name (e.g. Cabrera Valdes, Dominguez-Rodrigo, Bernaldo de Quiros, van den Bergh, Ameloot-Van der Heijden, d'Errico, de la Torre, etc.), followed by a comma and the initial(s) of the first name(s) (e.g. Wood, B., Herries, A.I.R., Tillier, A.-M.) Do not use spaces between multiple initials. To separate multiple authors' names, use a comma (e.g. Matmon, A., Ron, H., Chazan, M., Porat, N., Horwitz, L.K.). Do not use "and" or "&" to separate names.

The attribute **url** (Uniform Resource Locator) contains the internet address of a publication source in standard format (e.g. https://www.sajs.co.za/). Alternatively, a Digital Object Identifier (DOI) may be entered instead, using the format: https://doi.org/10.1002/evan.21870.

The attribute **access_date** represents the date a web page was last accessed.

The attribute **comments** contains general comments about the publication source.

The attribute **owner** automatically indicates the last person who entered or changed the dataset.

The attribute **created** automatically indicates the date this dataset was originally entered. An additional attribute **mod_history** automatically records the modification history of a dataset including the names of users who made previous changes and the dates those changes were made.

Table: edition

The primary table edition provides the year of publication, the volume and issue of a publication source.

**Primary table: edition**			
Attribute	Type	Key	Possible values
publication_source_id_source	Integer	Primary key/ Foreign key	[chosen from list]
idedition	Integer	Primary key	[automatically generated]
publication_year	Integer		1974, 2009
volume	Integer		[automatically generated]
issue	String		1, 2, 5-7, XIII, XV-XVII, XIX/XX
comments	String		general comments
owner	String		Dalai Lama
created	Date		[automatic date stamp]

The paired attributes **publication_source_id_source** and **idedition** define the volume and issue of a given book or journal. Both attributes are automatically generated, consecutive numbers.

The attribute **publication_year** contains the year in which the publication appeared. In rare cases, the year may be unknown, in which case you should enter “9999”.

The attribute **volume** contains the number of the volume which contains the given issue. The volume must be an integer. In the case of some annual publications (e.g. yearbooks), the volume may be the year in which the publication appeared.

The attribute **issue** contains the number of the issue in which the publication appeared. The issue consists of an alphanumeric string, and can be a single number, a series of numbers, roman numerals, or text.

The attribute **comments** contains general comments about the volume or issue.

The attribute **owner** automatically indicates the last person who entered or changed the dataset.

The attribute **created** automatically indicates the date this dataset was originally entered. An additional attribute **mod_history** automatically records the modification history of a dataset including the names of users who made previous changes and the dates those changes were made.

Table: publication

The primary table publication specifies the title, author and page numbers of an article in a journal, a contribution to a book, an entire book or a database entry in a publication source. An abstract may be added here.

**Primary table: publication**			
Attribute	Type	Key	Possible values
edition_id_source	Integer	Primary key/ Foreign key	[chosen from list]
edition_idedition	Integer	Primary key/ Foreign key	[chosen from list]
idpublication	Integer	Primary key	[automatically generated]
title	String		Out of Africa, Datenbanksysteme
publication_type	String		book, article, inbook, etc.
author	String		Bolus, M., Conard, N.J., Kandel, A.W., Kemper (Sr.), Eickler
abstract	String		[enter abstract here]
pages	String		18-32, 480-525
doi	String		10.1002/evan.21870
comments	String		general comments
owner	String		Englebert Humperdink
created	Date		[automatic date stamp]

The paired attributes **edition_id_source** and **edition_idedition** define the publication source and edition of the given book or journal. Both attributes are automatically generated, consecutive numbers.

The attribute **idpublication** is an automatically generated, consecutive number of the dataset.

The attribute **title** represents the name of an article, a book or a database.

The attribute **publication_type** represents the type of publication, for example: article or journal article; book (entire book); book section or inbook (chapter of book); thesis (Ph.D., M.A. or B.A.); conference proceedings; database; website; technical report; unpublished work, etc.

The attribute **author** contains the name(s) of the author(s) of a publication. Names should be entered using the complete last name (e.g. Cabrera Valdes, Dominguez-Rodrigo, Bernaldo de Quiros, van den Bergh, Ameloot-Van der Heijden, d'Errico, de la Torre, etc.), followed by a comma and the initial(s) of the first name(s) (e.g. Wood, B., Herries, A.I.R., Tillier, A.-M.) Do not use spaces between multiple initials. To separate multiple authors' names, use a comma (e.g. Matmon, A., Ron, H., Chazan, M., Porat, N., Horwitz, L.K.). Do not use "and" or "&" to separate names.

The attribute **abstract** contains a short summary taken from the publication.

The attribute **pages** contains the page numbers of a publication separated by a simple hyphen. Do not use long dashes or spaces. In cases where an article has no page numbers, use the digital article number instead.

The attribute **doi** contains the Digital Object Identifier for a publication. The format may be entered in two ways: https://doi.org/10.1002/evan.21870, or simply as 10.1002/evan.21870.

The attribute **comments** contains general comments about the publication.

The attribute **owner** automatically indicates the last person who entered or changed the dataset.

The attribute **created** automatically indicates the date this dataset was originally entered. An additional attribute **mod_history** automatically records the modification history of a dataset including the names of users who made previous changes and the dates those changes were made.

Publication data

Table: publication_desc_locality (publication describes locality)

The linking table publication_desc_locality creates a link between a locality and the publication describing this locality.

Linking table: publication_desc_locality			
Attribute	Type	Key	Possible values
locality_idlocality	String	Primary key/ Foreign key	[chosen from list] Geelbek_Alice, Hohle Fels
publication_id_source	Integer	Primary key/ Foreign key	[chosen from list]
publication_idedition	Integer	Primary key/ Foreign key	[chosen from list]
publication_idpublication	Integer	Primary key/ Foreign key	[chosen from list]
owner	String		David Hilbert
created	Date		[automatic date stamp]

The attribute **locality_idlocality** creates a link to a dataset in the table locality.

The threefold attributes: **publication_id_source**, **publication_idedition** and **publication_idpublication** create a link to a dataset in the table publication.

The attribute **owner** automatically indicates the last person who entered or changed the dataset.

The attribute **created** automatically indicates the date this dataset was originally entered. An additional attribute **mod_history** automatically records the modification history of a dataset including the names of users who made previous changes and the dates those changes were made.

Table: publication_desc_assemblage (publication describes assemblage)

The linking table publication_desc_assemblage creates a link between an assemblage and the publication describing this assemblage.

Linking table: publication_desc_assemblage			
Attribute	Type	Key	Possible values
assemblage_idlocality	String	Primary key/ Foreign key	[chosen from list] Geelbek_Alice, Hohle Fels
assemblage_idassemblage	Integer	Primary key/ Foreign key	[chosen from list]
publication_id_source	Integer	Primary key/ Foreign key	[chosen from list]
publication_idedition	Integer	Primary key/ Foreign key	[chosen from list]
publication_idpublication	Integer	Primary key Foreign key	[chosen from list]
ecoprofile_class	String		Fynbos, TBSh
owner	String		Nancy Sinatra
created	Date		[automatic date stamp]

The paired attributes **assemblage_idlocality** and **assemblage_idassemblage** create a link to a dataset in the table assemblage.

The threefold attributes **publication_id_source**, **publication_idedition** and **publication_idpublication** create a link to a dataset in the table publication.

The attribute **ecoprofile_class** describes the ecological setting infered by the author from faunal datasets. The classification systems differ by region and are externally defined. Possible values are for instance fynbos, a biome valid in South Africa, or TBSh, a quantitatively defined type of tropical shrubland valid in African and Asian tropic systems.

The attribute **owner** automatically indicates the last person who entered or changed the dataset.

The attribute **created** automatically indicates the date this dataset was originally entered. An additional attribute **mod_history** automatically records the modification history of a dataset including the names of users who made previous changes and the dates those changes were made.

Table: publication_desc_geolayer (publication describes geological layer)

The linking table publication_desc_geolayer creates a link between a geological layer and the publication describing this geological layer.

Linking table: publication_desc_geolayer			
Attribute	Type	Key	Possible values
geological_layer_idlocality	String	Primary key/ Foreign key	[chosen from list] Geelbek_Alice, Hohle Fels
geological_layer_name	String	Primary key/ Foreign key	[chosen from list] L23, 3a, VII, SGS
publication_id_source	Integer	Primary key/ Foreign key	[chosen from list]
publication_idedition	Integer	Primary key/ Foreign key	[chosen from list]
publication_id publication	Integer	Primary key/ Foreign key	[chosen from list]
owner	String		Othello
created	Date		[automatic date stamp]

The paired attributes **geological_layer_idlocality** and **geological_layer_name** create a link to a dataset in the table geological_layer.

The threefold attributes **publication_id_source**, **publication_idedition** and **publication_idpublication** create a link to a dataset in the table publication.

The attribute **owner** automatically indicates the last person who entered or changed the dataset.

The attribute **created** automatically indicates the date this dataset was originally entered. An additional attribute **mod_history** automatically records the modification history of a dataset including the names of users who made previous changes and the dates those changes were made.

Table: publication_desc_geostrat (publication describes geological stratigraphy)

The linking table publication_desc_ geostrat creates a link between a geological stratigraphy and the publication describing this geological stratigraphy.

**Linking table: publication_desc_ geostrat**			
Attribute	Type	Key	Possible values
geostratigraphy_idgeostrat	String	Primary key/ Foreign key	[chosen from list] 1, 2, Sibudu_BMOD-OMOD2
publication_id_source	Integer	Primary key/ Foreign key	[chosen from list]
publication_idedition	Integer	Primary key/ Foreign key	[chosen from list]
publication_id publication	Integer	Primary key/ Foreign key	[chosen from list]
owner	String		Pieter Bruegel
created	Date		[automatic date stamp]

The attribute **geostratigraphy_idgeostrat** creates a link to a dataset in the table geological_stratigraphy.

The threefold attributes **publication_id_source**, **publication_idedition** and **publication_idpublication** create a link to a dataset in the table publication.

The attribute **owner** automatically indicates the last person who entered or changed the dataset.

The attribute **created** automatically indicates the date this dataset was originally entered. An additional attribute **mod_history** automatically records the modification history of a dataset including the names of users who made previous changes and the dates those changes were made.

Table: publication_desc_archlayer (publication describes archaeological layer)

The linking table publication_desc_archlayer creates a link between an archaeological layer and the publication describing this archaeological layer.

**Linking table: publication_desc_archlayer**			
Attribute	Type	Key	Possible values
archaeologic_layer_idlocality	String	Primary key/ Foreign key	[chosen from list] Geelbek_Alice, Hohle Fels
archaeological_layer_name	String	Primary key/ Foreign key	[chosen from list] AH I, IIa, IIIcf, Ve
publication_id_source	Integer	Primary key/ Foreign key	[chosen from list]
publication_idedition	Integer	Primary key/ Foreign key	[chosen from list]
publication_id publication	Integer	Primary key/ Foreign key	[chosen from list]
owner	String		Robert de Niro
created	Date		[automatic date stamp]

The paired attributes **archaeologic_layer_idlocality** and **archaeological_layer_name** create a link to a dataset in the table archeological_layer.

The threefold attributes **publication_id_source**, **publication_idedition** and **publication_idpublication** create a link to a dataset in the table publication.

The attribute **owner** automatically indicates the last person who entered or changed the dataset.

The attribute **created** automatically indicates the date this dataset was originally entered. An additional attribute **mod_history** automatically records the modification history of a dataset including the names of users who made previous changes and the dates those changes were made.

Table: publication_desc_archstrat (publication describes archaeological stratigraphy)

The linking table publication_desc_archstrat creates a link between an archaeological stratigraphy and the publication describing this archaeological stratigraphy.

**Linking table: publication_desc_ archstrat**			
Attribute	Type	Key	Possible values
archstratigraphy_idarchstrat	Integer	Primary key/ Foreign key	[chosen from list]
publication_id_source	Integer	Primary key/ Foreign key	[chosen from list]
publication_idedition	Integer	Primary key/ Foreign key	[chosen from list]
publication_id publication	Integer	Primary key/ Foreign key	[chosen from list]
owner	String		Stella McCarthy
created	Date		[automatic date stamp]

The attribute **archstratigraphy_idarchstrat** creates a link to a dataset in the table archaeological_stratigraphy.

The threefold attributes **publication_id_source**, **publication_idedition** and **publication_idpublication** create a link to a dataset in the table publication.

The attribute **owner** automatically indicates the last person who entered or changed the dataset.

The attribute **created** automatically indicates the date this dataset was originally entered. An additional attribute **mod_history** automatically records the modification history of a dataset including the names of users who made previous changes and the dates those changes were made.

Table: publication_desc_humanremains (publication describes human remains)

The hybrid table publication_desc_humanremains provides further specifications about the fossils listed in the table humanremains and links the publication describing these results. These descriptions can be based on either physical specimens or genetic results, both of which provide additional insight into the fossils’ relevant characteristics. Each dataset is linked to three tables that describe the publication of the relevant information (e.g. publication, database).

**Hybrid table: publication_desc_humanremains**			
Attribute	Type	Key	Possible values
humanremains_idlocality	String	Primary key/ Foreign key	Geelbek_Alice, Hohle Fels
humanremains_idassemblage	Integer	Primary key/ Foreign key	[chosen from list]
humanremains_idhumanremains	Integer	Primary key/ Foreign key	[chosen from list]
publication_id_source	Integer	Primary key/ Foreign key	[chosen from list]
publication_idedition	Integer	Primary key/ Foreign key	[chosen from list]
publication_idpublication	Integer	Primary key/ Foreign key	[chosen from list]
genus	String		Australopithecus, Homo
species	String		afarensis, sapiens, n.c.
sex	String		M/F
genetic_sample_source	String		bone, tooth, sediment, block
body_mass_min	Integer		42, 28, 60
body_mass_max	Integer		68, 45, 110
age	String		adult, juvenile, subadult
brain_volume	String		1100, >800, 520-525
brain_volume_ method	String		Water immersion of reconstructed endocast
brain_mass	Integer		200, 348, 137
roceeh_default	Boolean		true, false
comments	String		general comments
owner	String		Lawrence of Arabia
created	Date		[automatic date stamp]

The threefold attributes **humanremains_idlocality**, **humanremains_idassemblage** and **humanremains_idhumanremains** create a link to a dataset in the table humanremains.

The threefold attributes **publication_id_source**, **publication_idedition** and **publication_idpublication** create a link to a dataset in the table publication.

The attribute **genus** contains the genus to which the described fossil was attributed. If the species is indeterminate or not communicated, the genus alone is sufficient. If no information can be found at all, "n.c." (not communicated) indicates the absence of information. Possible entries are: Australopithecus, Paranthropus, Homo, n.c.

The attribute **species** contains the species to which the described fossil was attributed. If the species is indeterminate or not communicated, the genus alone is sufficient. If no information can be found at all, "n.c." (not communicated) indicates the absence of information. Possible entries are: afarensis, sapiens, erectus, n.c.

The attribute **sex** describes the sex of the hominid if it can be determined. Two values are permitted: "M" for male and "F" for female. If the sex is indeterminate, no entry is required.

The attribute **genetic_sample_source** is used for publications about human remains which identify the genus and species of a hominin based on genetic analysis. Fill in this attribute only in cases where a sample yields diagnostic genetic material. (Do not use, if the results are not informative.) This attribute indicates the source of the sample. Possible entries are selected from a fixed list including: bone, tooth, petrous, sediment (i.e. from loose sediment), or block (i.e. from a hardened micromorphological sample). Only one entry may be selected. Additional entries may be added to the list only after discussion with the advisors. N.B.: When human DNA is present, information about body mass, age and brain size should not be entered.

The attribute **body_mass_min** indicates the minimum reconstructed body mass in kilograms if such reconstruction is possible or available for the fossil. (Do not complete for genetic data.)

The attribute **body_mass_max** indicates the maximum reconstructed body mass in kilograms if such reconstruction is possible or available for the fossil. (Do not complete for genetic data.)

The attribute **age** indicates the age category to which the fossil can be attributed. Only six values are possible: infant, juvenile, subadult, adult, mature and senile. (Do not complete for genetic data.)

The attribute **brain_volume** indicates the cerebral volume determined for the fossil in cubic centimeters. This entry applies only to cranial remains. (Do not complete for genetic data.)

The attribute **brain_volume_method** contains the description of the procedure used for estimating the cerebral volume. This entry applies only to datasets that contain values in the attribute **brain volume**. Possible values are: "water immersion of reconstructed endocast", "based on external cranial measurements", etc. (Do not complete for genetic data.)

The attribute **brain_mass** indicates the reconstructed value of the cerebral mass determined for the fossil in grams. This entry applies only to datasets that contain values in the attribute **brain_volume**. (Do not complete for genetic data.)

The attribute **roceeh_default** indicates whether the dataset represents ROCEEH’s default interpretation of a given human remain. True (t) indicates that this choice is preferred; false (f) indicates that this choice is not preferred. This attribute is only used when publications do not agree on the taxonomic classification of a human fossil. If there is a consensus on the fossil, no data are entered (NULL). (Do not complete for genetic data.)

The attribute **comments** contains general comments about the human remains.

The attribute **owner** automatically indicates the last person who entered or changed the dataset.

The attribute **created** automatically indicates the date this dataset was originally entered. An additional attribute **mod_history** automatically records the modification history of a dataset including the names of users who made previous changes and the dates those changes were made.

Table: publication_desc_climate (publication describes climate)

The linking table publication_desc_climate creates a link between the climate analytical results and the publication describing these results.

**Linking table: publication_desc_climate**			
Attribute	Type	Key	Possible values
publication_id_source	Integer	Primary key/ Foreign key	[chosen from list]
publication_idedition	Integer	Primary key/ Foreign key	[chosen from list]
publication_idpublication	Integer	Primary key/ Foreign key	[chosen from list]
climate_idclimate	Integer	Primary key/ Foreign key	[chosen from list]
owner	String		Theodor Heuss
created	Date		[automatic date stamp]

The threefold attributes **publication_id_source**, **publication_idedition** and **publication_idpublication** create a link to a dataset in the table publication.

The attribute **climate_idclimate** create a link to a dataset in the table climate.

The attribute **owner** automatically indicates the last person who entered or changed the dataset.

The attribute **created** automatically indicates the date this dataset was originally entered. An additional attribute **mod_history** automatically records the modification history of a dataset including the names of users who made previous changes and the dates those changes were made.

Table: publication_desc_vegetation (publication describes vegetation)

The linking table publication_desc_vegetation creates a link between the vegetation analytical results and the publication describing these results.

Linking table: publication_desc_vegetation			
Attribute	Type	Key	Possible values
publication_id_source	Integer	Primary key/ Foreign key	[chosen from list
publication_idedition	Integer	Primary key/ Foreign key	[chosen from list
publication_idpublication	Integer	Primary key/ Foreign key	[chosen from list
vegetation_idvegetation	Integer	Primary key/ Foreign key	[chosen from list
owner	String		Ursula Andress
created	Date		[automatic date stamp]

The threefold attributes **publication_id_source**, **publication_idedition** and **publication_idpublication** create a link to a dataset in the table publication.

The attribute **vegetation_idvegetation** create a link to a dataset in the table vegetation.

The attribute **owner** automatically indicates the last person who entered or changed the dataset.

The attribute **created** automatically indicates the date this dataset was originally entered. An additional attribute **mod_history** automatically records the modification history of a dataset including the names of users who made previous changes and the dates those changes were made.

Table: publication_desc_paleofauna (publication describes paleofauna)

The hybrid table publication_desc_paleofauna provides further specifications about the analytical results of faunal remains and links the publication describing these results. The table also allows the entry of data about the minimum number of individuals (MNI) for a given taxon.

**Hybrid table: publication_desc_paleofauna**			
Attribute	Type	Key	Possible values
publication_id_source	Integer	Primary key/ Foreign key	[chosen from list]
publication_idedition	Integer	Primary key/ Foreign key	[chosen from list]
publication_idpublication	Integer	Primary key/ Foreign key	[chosen from list]
paleofauna_idlocality	String	Primary key/ Foreign key	[chosen from list]
paleofauna_idassemblage	Integer	Primary key/ Foreign key	[chosen from list]
paleofauna_idtaxonomical_classification	Integer	Primary key/ Foreign key	[chosen from list]
paleofauna_species	String	Primary key/ Foreign key	[chosen from list]
mni	Integer		
method	String		
comments	String		general comments
owner	String		Winnie the Pooh
created	Date		[automatic date stamp]

The threefold attributes **publication_id_source**, **publication_idedition** and **publication_idpublication** create a link to a dataset in the table publication.

The fourfold attributes **paleofauna_idlocality**, **paleofauna_idassemblage**, **paleofauna_idtaxonomical_classification** and **paleofauna_species** create a link to a dataset in the table paleofauna.

The attribute **mni** contains the minimum number of individuals for each taxon in an assemblage. If it is indeterminate, no entry is required.

- The MNI calculation may only include finds that have been correctly identified taxonomically as well as anatomically. Finds which are too fragmentary to identify the exact anatomical position or which cannot be classified to a specific taxon must be excluded from the MNI calculation.
- Exact anatomical position means: if the correct anatomical position cannot be determined (e.g. M sup sin) or if the laterality remains unknown (e.g. McII), the item should be excluded from the MNI calculation.
- Rib fragments are only included if the anatomical position can be exactly determined.
- Exact taxonomic identification means: if the anatomical position is exactly identified but the taxonomical determination is impossible or insufficient, the finds should be excluded from the MNI calculation.
- If the data are taken from the literature, it must be ensured that the published MNI follows the given criteria. If this does not apply, the MNI should not be entered and the dataset will remain empty.

The attribute **method** provides the name of the method used for this analysis or describes the methodology.

The attribute **comments** contains general comments about the publication that describes paleofauna.

The attribute **owner** automatically indicates the last person who entered or changed the dataset.

The attribute **created** automatically indicates the date this dataset was originally entered. An additional attribute **mod_history** automatically records the modification history of a dataset including the names of users who made previous changes and the dates those changes were made.

Table: image

The primary table image contains images that are copyrighted. ROAD stores these images directly in the database as binary objects. The table contains specific information about the image as related to its copyright and permission to use the image. This information includes who the copyright holder is, whether an individual or an institution, as well as the type of license. Keywords describing the content of the image should be added to help make the image findable and searchable.

**Primary table: image**			
Attribute	Type	Key	Possible values
idimage	Integer	Primary key	[automatically generated]
image	String		[binary data]
copyrightholder_scientist_idscientist	Integer	Foreign key	[chosen from list]
copyrightholder_organization_idorganization	Integer	Foreign key	[chosen from list]
license	String		CC BY 4.0 (Attribution),<br>CC BY-SA 4.0 (Attribution Share Alike),<br>CC BY-NC 4.0 (Attribution NonCommercial), et al.
keywords	String		hominin, homo sapiens, humerus
comments	String		general comments
owner	String		Alexei Navalny
created	Date		[automatic date stamp]

The attribute **idimage** is the automatically generated, consecutive number of the image dataset.

The attribute **image** contains the actual image as a binary object saved in the database.

The attribute **copyrightholder_scientist_idscientist** identifies the person who has defined the license for the image.

The attribute **copyrightholder_organization_idorganization** identifies the organization which has defined the license for the image.

The attribute **license** contains information about the type of license (e.g. CC BY 4.0, CC BY-SA 4.0, etc.) For more details, see: https://creativecommons.org/licenses/). For non-standard copyright, please contact the database administrators (Z. Kanaeva or A. Kandel) at road@roceeh.net.

The attribute **keywords** contains words that describe the image. Use a comma to separate keywords (e.g. hominin, homo erectus, radius).

The attribute **comments** contains general comments about the image.

The attribute **owner** automatically indicates the last person who entered or changed the image dataset.

The attribute **created** automatically indicates the date this dataset was originally entered. An additional attribute **mod_history** automatically records the modification history of a dataset including the names of users who made previous changes and the dates those changes were made.

Project tables

Table: project

The primary table project defines the names of projects and describes their purpose. A project is essentially a device which allows a user to create a list of pre-selected assemblages and then use the list as a template for writing further queries. A project can be used when different scientists want to analyze a list of pre-selected assemblages for different characteristics. For example, a list of Neanderthal sites and assemblages dated to MIS 6 can be selected and then analyzed by different researchers to determine which raw materials, typologies, organic tools, human remains, animal remains or botanical remains are present in the same assemblages.

**Primary table: project**			
Attribute	Type	Key	Possible values
idproject	Integer	Primary key	[automatically generated]
name	String		MIS6
description	String		Brief text about project MIS6, Bolus 2018
created	Date		[automatic date stamp]

The attribute **idproject** is a unique, automatically generated, consecutive number assigned to each dataset.

The attribute **name** provides the name of the project.

The attribute **description** provides a brief description of the project. Be as specific as possible about the project and indicate who initiated the project, and in which year, e.g. Bolus 2018.

The attribute **created** automatically indicates the date this dataset was originally entered. An additional attribute **mod_history** automatically records the modification history of a dataset including the names of users who made previous changes and the dates those changes were made.

Table: project_select_assemblage

The look-up table project_select_assemblage creates a list of assemblages that the user wishes to analyze by linking a project to the selected assemblages. This linking table creates a collection of assemblages that a researcher can use as a template for writing further queries.

**Look-up table: project_select_assemblage**			
Attribute	Type	Key	Possible values
project_idproject	String	Primary key/ Foreign key	[chosen from list]
assemblage_idlocality	Integer	Primary key/ Foreign key	[chosen from list]
assemblage_idassemblage	Integer	Primary key/ Foreign key	[chosen from list]
comments	String		general comments
created	Date		[automatic date stamp]

The attribute **project_idproject** is a unique, automatically generated, consecutive number assigned to each dataset which is linked to the table project.

The paired attributes **assemblage_idlocality** and **assemblage_idassemblage** define the locality and assemblage which are part of a specific project.

The attribute **comments** contains general comments about the project_select_assemblage.

The attribute **created** automatically indicates the date this dataset was originally entered. An additional attribute **mod_history** automatically records the modification history of a dataset including the names of users who made previous changes and the dates those changes were made.

ROAD table attribute descriptions

Table: road_table_attr_desc

The primary table road_table_attr_desc contains information describing all of the tables and attributes used in ROAD. The descriptions contained in this table are the same as the information presented in this user's manual. Like this user's manual, each table in ROAD begins with a general overview. Then each attribute is described in further detail. General information about each table can be viewed in ROAD by clicking on the button "description" in the upper part of each table view. Specific information about each attribute can be viewed in ROAD by clicking on the blue and white question mark placed next to each attribute in each table view, as well as in the data entry form.

**Primary table: road_table_attr_desc**			
Attribute	Type	Key	Possible values
table_name	String	Primary key	locality, assemblage
attribute_name	String	Primary key	idlocality, idassemblage
description	Date		The attribute locality contains…
owner	String		Donald J. Trump
created	Date		[automatic date stamp]

The attribute **table_name** is the name of the table in ROAD that is described.

The attribute **attribute_name** is the name of the attribute in ROAD that is described.

The attribute **description** is a description of the table or attribute in ROAD.

The attribute **owner** automatically indicates the last person who entered or changed the dataset.

The attribute **created** automatically indicates the date this dataset was originally entered. An additional attribute **mod_history** automatically records the modification history of a dataset including the names of users who made previous changes and the dates those changes were made.

References

Behrensmeyer, A.K. 1978. Taphonomic and ecologic information from bone weathering. *Paleobiology* 4: 150-162. https://doi.org/10.1017/S0094837300005820

Cohen, K.M., Finney, S.C., Gibbard, P.L., Fan, J.-X. (2013; updated) The ICS International Chronostratigraphic Chart. Episodes 36: 199-204. [Last accessed on 19 Mar. 2021, https://stratigraphy.org/ICSchart/ChronostratChart2020-03.pdf]

Conrad, O. 2007. *SAGA–Entwurf, Funktionsumfang und Anwendung eines Systems für Automatisierte Geowissenschaftliche Analysen*. Ph.D. dissertation, Göttingen: University of Göttingen. [Last accessed on 19 Mar. 2021, https://docplayer.org/15522797-Saga-entwurf-funktionsumfang-und-anwendung-eines-systems-fuer-automatisierte-geowissenschaftliche-analysen.html]

Lisiecki, L.E., Raymo, M.E. 2005 A Pliocene-Pleistocene stack of 57 globally distributed benthic δ18O records. Paleooceanography 20, PA1003, https://doi.org/10.1029/2004PA001071, 2005, [Last accessed on 19 Mar. 2021, see also https://www.ncdc.noaa.gov/paleo-search/study/5847]

Olson, D.M., Dinerstein, E., Wikramanayake, E.D., Burgess, N.D., Powell, G.V.N., Underwood, E.C., D'Amico, J.A., Itoua, I., Strand, H.E., Morrison, J.C., Loucks, C.J., Allnutt, T. F., Ricketts, T.H., Kura, Y., Lamoreux, J.F., Wettengel, W.W., Hedao, P., Kassem, K.R. 2001. Terrestrial ecoregions of the world: A new map of life on Earth. *Bioscience* 51: 933-938. [Last accessed on 19 Mar. 2021, https://www.sciencebase.gov/catalog/item/508fece8e4b0a1b43c29ca22]

Radiocarbon, List of current and retired laboratories. 2021. See pdf at end of web page. [Last accessed on 19 Mar. 2021, http://radiocarbon.webhost.uits.arizona.edu/node/11]
