# Supplementary material for: The ROCEEH Out of Africa Database (ROAD): A large-scale research database serves as an indispensable tool for human evolutionary studies
Source: PLoS One. 2023 Aug 1;18(8):e0289513. doi: 10.1371/journal.pone.0289513 (PMC10393170; doi:10.1371/journal.pone.0289513)
Supplement: S2 File — The manual provides general information about the database, describes how to write queries, illustrates how to use the Map Module to visualize data. (PDF) [file pone.0289513.s004.pdf]

THE ROLE OF CULTURE IN EARLY EXPANSIONS OF HUMANS

ROAD Manual

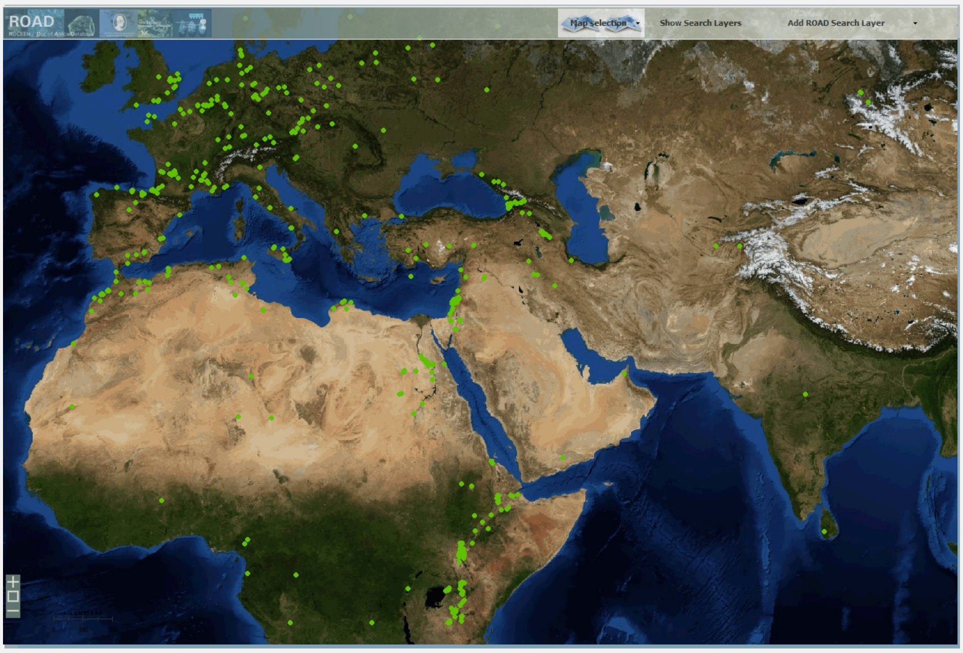

Table of contents

ROAD — The logical model 5

Localities, assemblages and geodata 6

Stratigraphy and dating 7

Detailed assemblage description 8

Bibliography 9

Data entry (under construction) 11

SQL Queries 13

SELECT and FROM clauses 13

SELECT clause and DISTINCT 14

ORDER BY clause 15

WHERE clause 16

Exercise Q-I 18

The ROADWeb Query Tool 19

FROM clause 19

SELECT and ORDER BY clauses 20

WHERE clause 20

Exercise Q-II 22

Joins 23

Tables 'locality' and 'assemblage' 24

Exercise Q-III 28

Joins in ROADWeb 29

Exercise Q-IV 30

Querying for age and dating with the ROADWeb Query Tool 31

Exercise Q-V 32

The ROAD Map Module 35

Answers (under construction) 41

Notes

ROAD — The logical model

ROAD contains published data about localities and assemblages of finds with information related to dating, geology and geography. Dating and geological data permit a chronological analysis of the datasets. Geographical data allow for the spatial analysis of datasets. Collected data cover the last three million years of geologic time (Plio-Pleistocene and Holocene). ROCEEH members enter the selected data from the literature and their own publications . The purpose of ROAD is to provide:

- Safe, long-term storage of a variety of archaeological, paleobiological and paleobotanical data
- Data for validation of models (for example agent-based models)
- Specific spatio-temporal data for visualization of expansion dynamics
- Specific data retrieval from the database
- Help in understanding the interaction of early humans and their environment by reconstructing of spatial and temporal patterns of hominin expansions

The structure of ROAD is divided into four main parts, described in more detail in the following pages:

a) Localities and assemblages;

b) Stratigraphy and dating;

c) Detailed assemblage descriptions

d) Bibliographic information

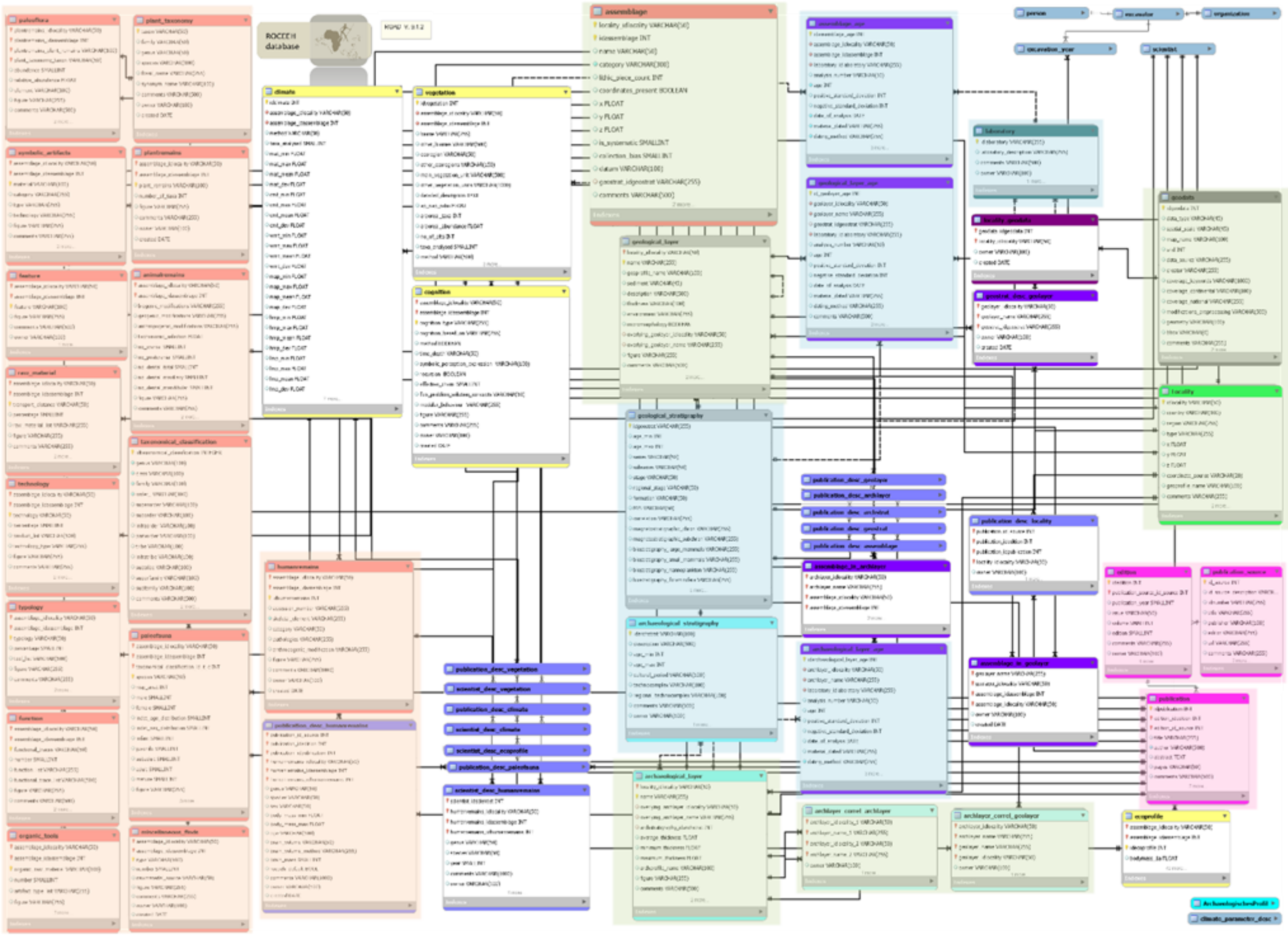

Localities, assemblages and geodata

This part of the database contains general information about localities including information about geology and geography, as well as a general description of the assemblages.

The locality table includes geographical coordinates for each locality. If you would like to display the results of your query on a map, the locality table will provide you with the required information.

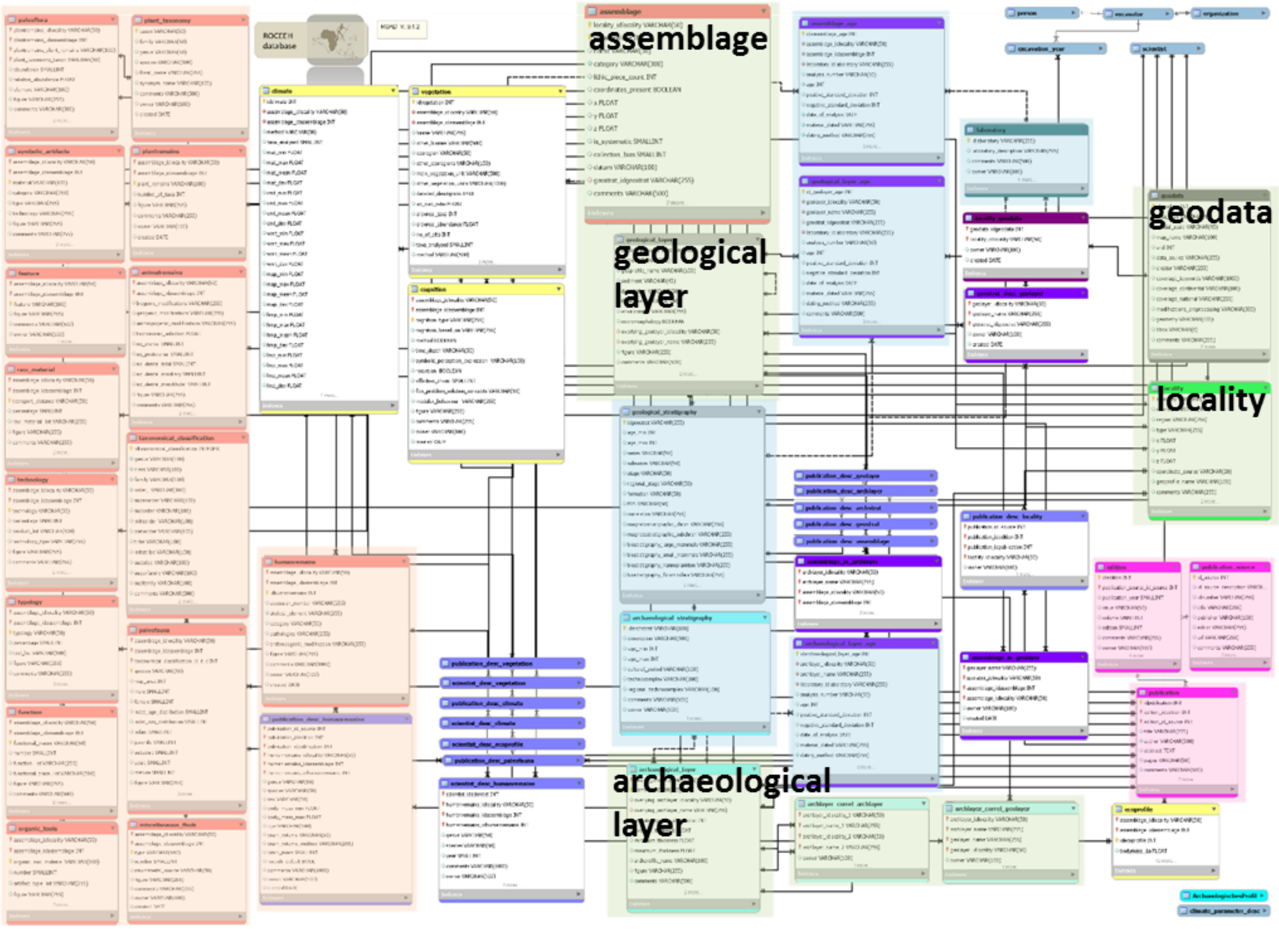

Stratigraphy and dating

Dating in ROAD includes stratigraphy and published data about the ages of sites, geological layers and assemblages.

The table ‘geological layer_age‘ contains information about the age of the geological layers. The age of a particular geological layer results from reports by an analytical laboratory for a single sample from the specific geolayer. When diverging dates for assemblages from the same layer are published, there may be a variety of age entries in the table ‘geological_layer_age‘.

The table ‘assemblage_age‘ contains information about the age of the assemblages. The age is based on results provided by an analytical laboratory for an individual sample from a particular assemblage.

The table ‘geological_stratigraphy‘ characterizes the geological age of a stratigraphic unit (consisting of one or more geolayers) in chronostratigraphical terms. This characterization of the geological age of a stratigraphic unit includes information about minimum and maximum ages. For a particular stratigraphic unit there can only be a single entry in the table geological stratigraphy. For most of the geological layers, relevant entries in the table ‘geological_stratigraphy‘ exist. The linking table ‘geostrat_desc_geolayer‘ establishes a connection between a stratigraphic unit and a geolayer.

Note that ROAD distinguishes between geological and archaeological stratigraphies. There is a corresponding set of tables for archaeological stratigraphy (i.e. ‘archaeological_stratigraphy‘ and ‘archaeological_layer_age‘) to store dates related to archaeological rather than geological layers. However, the link between the tables ‘archaeological_layer‘ and ‘archaeological_stratigraphy‘ has to be manually established by selecting a foreign key in the table ‘archaeological_layer‘.

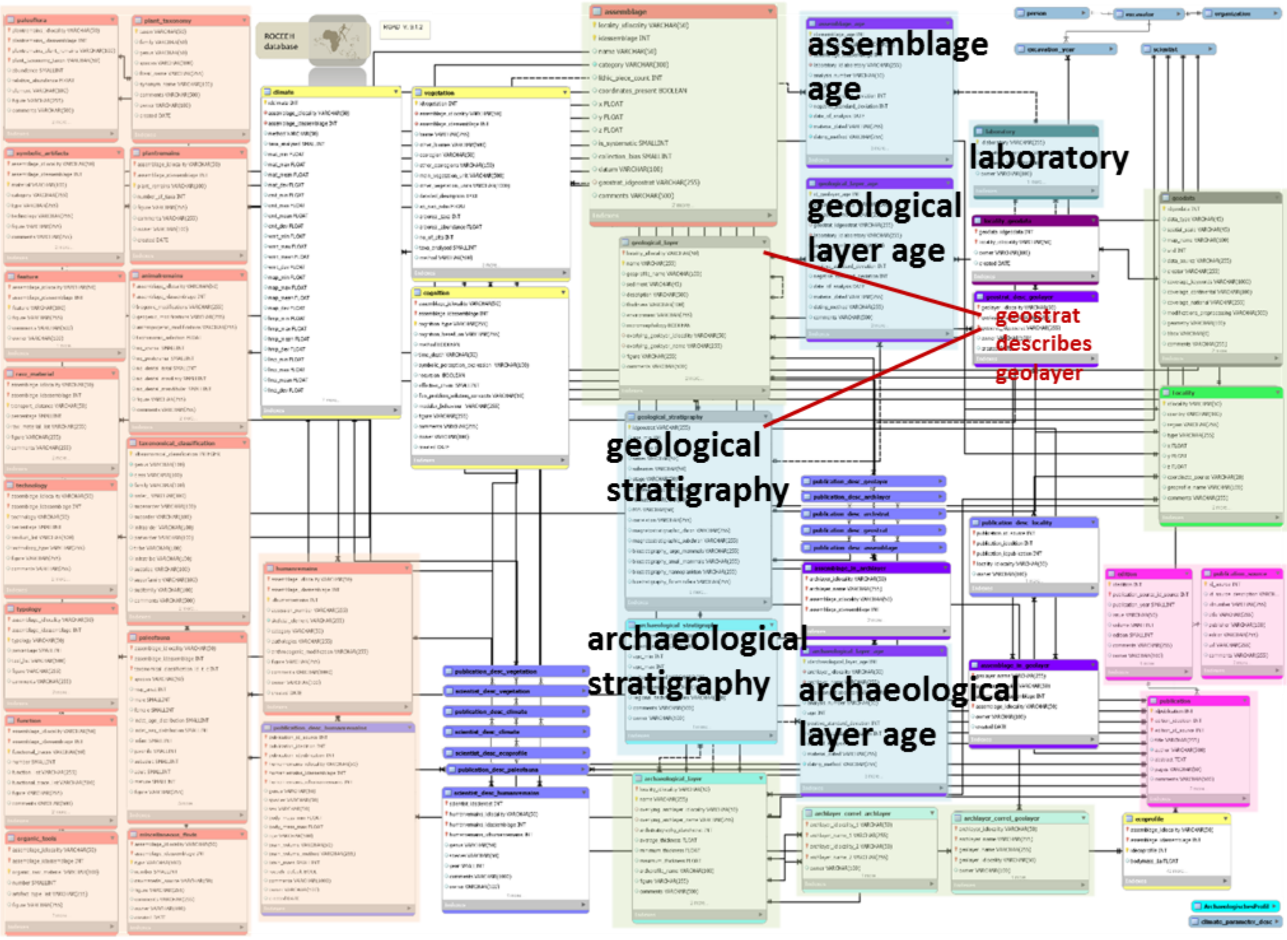

Detailed assemblage descriptions

Detailed assemblage descriptions include information based on interpretation and analysis of finds. ROAD categorizes assemblages of finds into several types: archaeological finds, human remains, faunal remains and botanical remains.

The analysis of archaeological finds yields information for further classification of the specimens, for example stone artifacts. Their description includes data on raw material, technology, typology and functional traces. Each of the four different ways to characterize an lithic assemblage are stored in different tables. Archaeological finds can also be symbolic artifacts which convey an abstract meaning. Symbolic artifacts are datasets that clearly represent art, music and/or ornaments. Their description is saved in the table ‘symbolic_artifacts‘.

Human remains are described in the table ‘humanremains‘. A human remain is a direct and substantial piece of evidence for the presence of fossil hominids at a particular locality. The description of human fossils in the table human remains includes information about skeletal elements, pathologies, and anthropogenic modifications. Every single human fossil represents an individual assemblage unless several elements belong to the same skeleton. Such cases occur in ROAD, but they are the exception rather than the rule.

The table ‘publication_desc_humanremains‘ provides further specifications about the specimens, for instance on taxonomic assignments, individual age and sex. In this way, different opinions about the classification of human fossils can be stored in ROAD. Since we collect all published variants, the final decision on fossil classification is left to the user. Thus, a straightforward query for a particular human taxa will retrieve all specimens if human taxonomy is left unsupervised. For this reason, unsupervised mapping of the distribution of human taxa may lead to ambiguous outcomes and is not usually recommended!

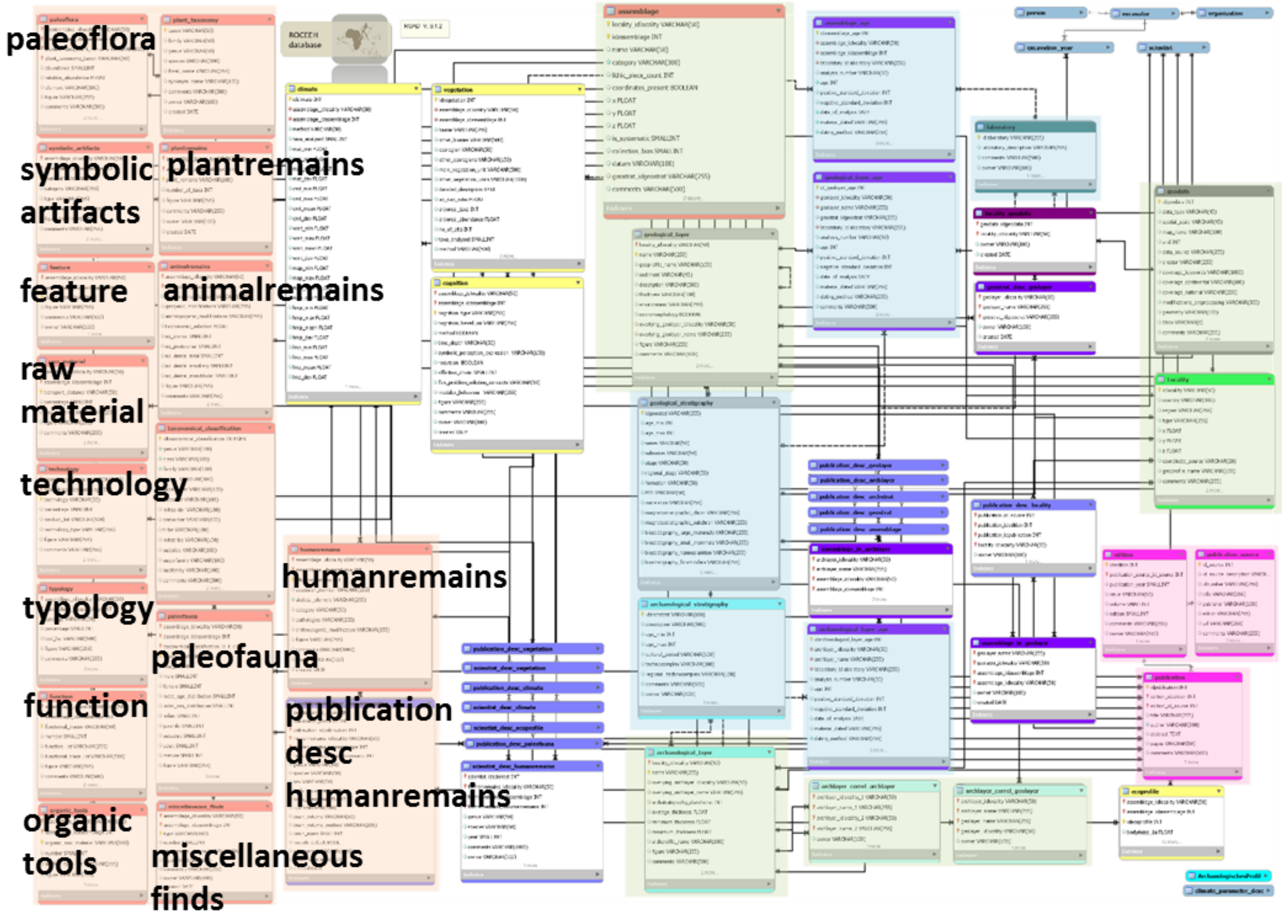

Bibliography

Bibliographic information describes a publication resource and typically contains bibliographic data such as author name(s), the title of the publication, the name of the publisher, the title of the journal, as well as data on volume, issue, publication year and an abstract.

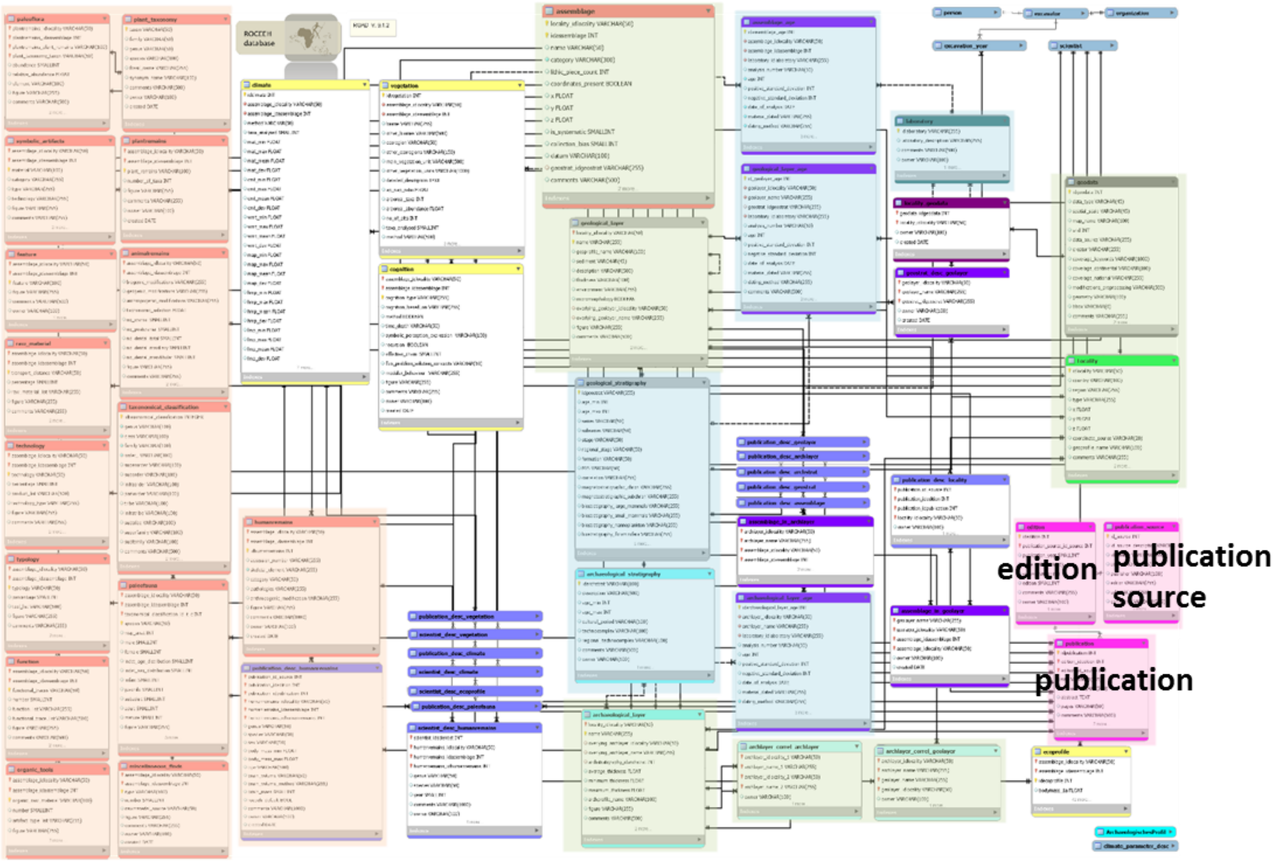

Notes

Data entry

Under construction

Notes

SQL queries

A database is not just a way of structuring data: it also defines a set of operations that can be performed on the data. For relational databases this set of operations uses a language called SQL (Structured Query Language). SQL statements are classified into several classes. One of these classes is called Data Manipulation Language (DML).

The DML class includes SELECT statements. All operations of this class operate on data. Data cannot be stored in a database without corresponding database objects. For example, a data record cannot exist without a table. A table, regardless of whether it contains data or not, is one database object. There are also operations for database objects. These operations belong to the DDL (Data Definition Language) of SQL operations.

Table: locality

**idlocality**	**country**	**type**	**x**	**y**
Blombos Cave	South Africa	Cave	21.218	-34.413
Haalenberg	Namibia	Rock shelter	15.467	-26.586
Swartkrans	South Africa	Cave	27.755	-25.969
Sibudu Cave	South Africa	Rock shelter	31.086	-29.522
Tiras 5	Namibia	Rock shelter	16.583	-26.208
Linksfield	South Africa	Open air	28	-26.17

An example table is shown above and is called ‘locality‘. The table ‘locality‘ has five columns (= attributes) and six rows (= data records). The table ‘locality‘ contains data about six localities. Four of these localities are located in South Africa and two are in Namibia. Two of them are of the type ‘Cave‘, three are of the type ‘Rock shelter‘ and the last one is of the type ‘Open air‘.

SELECT and FROM clauses

In order to retrieve data from the table, the table must be queried. An SQL SELECT statements is used to do this. The complete syntax of such queries may be quite complex. Its basic structure, however, is not complicated. We start with the basic select statement specification. This illustrates that we may write and perform powerful queries even though the basic statement is not difficult to understand.

```
SELECT
        …

FROM
        …

[WHERE … ];
```

The basic SQL SELECT statement on the right is divided into a select list (SELECT), a table list (FROM) and optional qualifications (WHERE). The select list (keyword SELECT) represents the part which lists the columns to be returned. It is also called the SELECT clause. If the query should return all columns of the queried table, we can use the alias ‘ * ‘ (asterisk) to denote all column names. The table list with the keyword FROM represents the part listing the tables from which we retrieve data. It is also called the FROM clause. With the simple query shown on the following page, we simply list all entries in the table ‘locality‘.

```
SELECT
        idlocality,
        country,
        type,
        x,
        y
```

SELECT clause

FROM clause

or alternatively:

```
SELECT
        *

FROM
```

SELECT clause

FROM clause

idlocality	country	type	x	y
Blombos Cave	South Africa	Cave	21.218	-34.413
Haalenberg	Namibia	Rock shelter	15.467	-26.586
Swartkrans	South Africa	Cave	27.755	-25.969
Sibudu Cave	South Africa	Rock shelter	31.086	-29.522
Tiras 5	Namibia	Rock shelter	16.583	-26.208
Linksfield	South Africa	Open air	28	-26.17

The result is the same no matter how the SELECT clause is specified.

SELECT clause and DISTINCT

```
SELECT
        *
FROM
        locality
WHERE
        type = 'Cave'
;
```

The following query retrieves all attributes from the table 'locality', which are of the type 'Cave'. (The WHERE clause will be described in further detail in one of the following sections).

idlocality	country	type	x	y
Blombos Cave	South Africa	Cave	21.218	-34.413
Swartkrans	South Africa	Cave	27.755	-25.969

```
SELECT
        country,
        type
FROM
        locality
WHERE
        type = 'Cave'
;
```

In this case both resulting rows are unique. However, if we restrict our selection to country and type, both of the rows are identical.

country	type
South Africa	Cave
South Africa	Cave

```
SELECT
      DISTINCT
      country,
      type
FROM
      locality
WHERE
      type = 'Cave'
;
```

To eliminate duplicate records in the results and to fetch only unique records the SQL keyword DISTINCT can be used in the SELECT clause. In this way, extended queries yield unique results. The result consists of only one row.

country	type
South Africa	Cave

```
SELECT
      DISTINCT
      idlocality,
      country,
      type
FROM
      locality
WHERE
      type = 'Cave'
;
```

The example below shows another SELECT query. In this case, however, the attribute 'idlocality' distinguishes both of the results. Adding DISTINCT in the SELECT clause does not lead to a smaller number of results, because both cases differ.

idlocality	country	type
Blombos Cave	South Africa	Cave
Swartkrans	South Africa	Cave

ORDER BY clause

```
SELECT
      idlocality,
      country,
      type,
      x,
      y
FROM
      locality
ORDER BY
      idlocality      } ORDER BY clause
;
```

If we wish to sort search results by one or more columns the ORDER BY clause has to be specified, as shown here:

idlocality	country	type	x	y
Blombos Cave	South Africa	Cave	21.218	-34.413
Haalenberg	Namibia	Rock shelter	15.467	-26.586
Linksfield	South Africa	Open air	28	-26.17
Sibudu Cave	South Africa	Rock shelter	31.086	-29.522
Swartkrans	South Africa	Cave	27.755	-25.969
Tiras 5	Namibia	Rock shelter	16.583	-26.208

WHERE clause

```
SELECT
        *
FROM
        locality
WHERE
        Idlocality =
'Sibudu Cave'
;
```

WHERE clause

A query can be additionally qualified by adding a WHERE clause specifying the rows which are wanted. The WHERE clause contains a Boolean expression (true/false value). In the example shown, the Boolean expression is: idlocality = 'Sibudu Cave'.

**idlocality**	**country**	**type**	**x**	**y**
Sibudu Cave	South Africa	Rock shelter	31.086	-29.522

A WHERE clause can be specified by using comparative or logical operators. In the example above, we used the comparison operator '=' . Other examples include: >, <, >=, =<, like, ilike, in, not in.

The operators 'like', 'not like' and 'ilike' require some explanation. With these operators we may search for strings, i.e. for partial data: For example, we may search for entries containing the letter 'a' in type. However, the string to be searched for needs to be bracketed by the wildcard '%', because any combination of letters and/or characters may come before or after the 'a'.

```
SELECT
        *
FROM
        locality
WHERE
        type  like '%a%';
```

**idlocality**	**country**	**type**	**x**	**y**
Blombos Cave	South Africa	Cave	21.218	-34.413
Haalenberg	Namibia	Rock shelter	15.467	-26.586
Swartkrans	South Africa	Cave	27.755	-25.969
Sibudu Cave	South Africa	Rock shelter	31.086	-29.522
Tiras 5	Namibia	Rock shelter	16.583	-26.208
Linksfield	South Africa	Open air	28	-26.17

If the percentage signs are omitted, the 'like' operator acts as an '=' operator. The 'not like' operator yields inverse results. It excludes all entries containing the letter 'a' from the resulting table. The operator 'ilike' can be used instead of 'like' to make the match insensitive to case.

The WHERE clause may include more than one condition. The following statement has two conditions which are combined with the OR operator. As a result, the SELECT statement lists all the records of the table locality which are of type 'Rock shelter' or 'Cave'. As the locality Linksfield is of type 'Open air', it is not retrieved by this query.

```
SELECT
	*
FROM
	locality
WHERE
	type = 'Rock shelter'
OR
	type = 'Cave';
```

idlocality	country	type	x	y
Blombos Cave	South Africa	Cave	21.218	-34.413
Haalenberg	Namibia	Rock shelter	15.467	-26.586
Swartkrans	South Africa	Cave	27.755	-25.969
Sibudu Cave	South Africa	Rock shelter	31.086	-29.522
Tiras 5	Namibia	Rock shelter	16.583	-26.208
Linksfield	South Africa	Open air	28	-26.17

To combine multiple conditions, the **AND** and **OR** operators should be used. The AND operator retrieves records fulfilling both conditions, the OR operator retrieves records complying with either one of them.

What happens if we replace the OR operator in the query by the AND operator? The OR operator is less restrictive and retrieves more records. If multiple ANDs and ORs are used in the WHERE clause, the AND operators are evaluated first, and then the OR operators after. If we wish to change the evaluation order, we need to use brackets.

```
SELECT
	*
FROM
	locality
WHERE
	country = 'South Africa'
AND
	type = 'Cave'
OR
	type = 'Rock shelter';
```

```
SELECT
	*
FROM
	locality
WHERE
	country = 'South Africa'
AND
	(type = 'Cave'
OR
	type = 'Rock shelter');
```

Compare both of these queries. Where is the difference?

The queries have the same SELECT clauses and the same FROM clauses, but the WHERE clauses are different. In the WHERE clause on the right hand query, brackets are used for the OR operator. These brackets change the evaluation order in the WHERE clause. In this query, the type = 'Cave' OR type = 'Rock shelter' criteria are evaluated first, and then the criterion country = 'South Africa' is applied. In the query shown on the left hand side, the criteria country = 'South Africa' AND type = 'Cave' are applied first, and the criterion type = 'Rock shelter' after. In view of this difference, will the results of both queries be the same?

idlocality	country	type
Blombos Cave	South Africa	Cave
Haalenberg	Namibia	Rock shelter
Linksfield	South Africa	Open air
Sibudu Cave	South Africa	Rock shelter
Swartkrans	South Africa	Cave
Tiras 5	Namibia	Rock shelter

idlocality	country	type
Blombos Cave	South Africa	Cave
Haalenberg	Namibia	Rock shelter
Linksfield	South Africa	Open air
Sibudu Cave	South Africa	Rock shelter
Swartkrans	South Africa	Cave
Tiras 5	Namibia	Rock shelter

Exercise Q-I

Table: Locality

**idlocality**	**country**	**type**	**x**	**y**
Blombos Cave	South Africa	Cave	21.218	-34.413
Haalenberg	Namibia	Rock shelter	15.467	-26.586
Swartkrans	South Africa	Cave	27.755	-25.969
Sibudu Cave	South Africa	Rock shelter	31.086	-29.522
Tiras 5	Namibia	Rock shelter	16.583	-26.208
Linksfield	South Africa	Open air	28	-26.17

1. Write a query to (i) find all localities of type 'Open air' or 'Cave' and (ii) order the query results by type.

2. Write a query: find all localities in South Africa with 'Cave' as a part of idlocality by using the 'like' family of operators.

3. Fill the gap, i.e. specify the attributes to be selected (…), so that the results consist of x rows:

```
SELECT
    DISTINCT
    ….
FROM
    locality
WHERE
    Country = 'South Africa'
```

	**Attributes**
1 row	
3 rows	
4 rows	

(*) The correct results of the exercises are shown at the end of this manual.

Queries — The ROADWeb Query Tool

The ROADWeb query tool allows the user to compose and perform their own SQL queries. The flexibility of the tool is among its advantages. It permits easy writing of SELECT queries for the ROAD database. However, one of the challenges is that knowledge of the ROAD structure is required and not all features of SQL SELECT queries can be used.

FROM clause

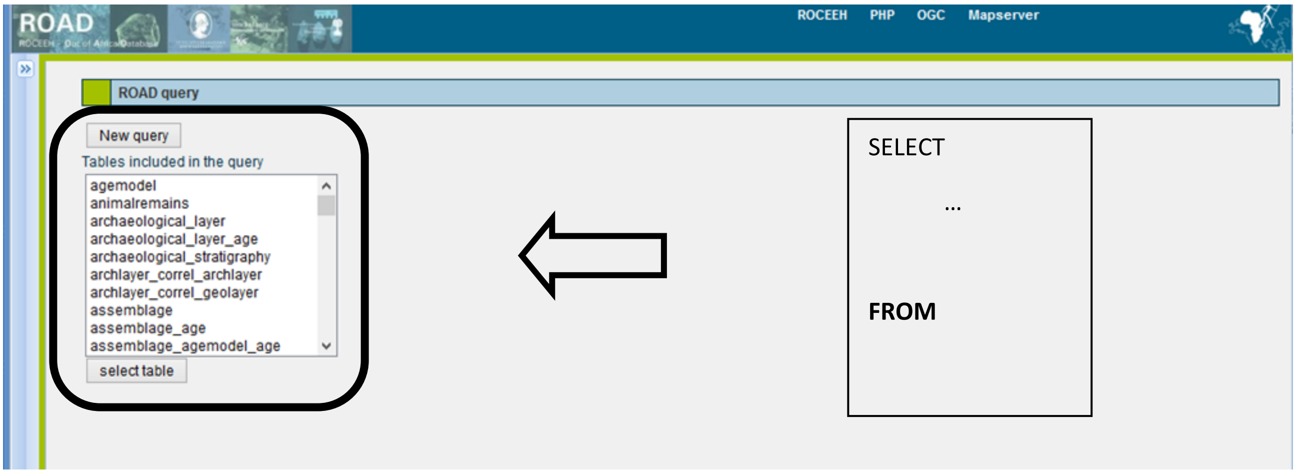

Login to the ROAD database and chose “query data“ from the menu on the left. A list with existing queries opens. Chose “new query“.

For every clause of the SELECT query, the ROADWeb query tool has one or more graphical elements. At first the tool shows the user a multiple selection box with ROAD tables. This step corresponds to the FROM clause. Choose tables and confirm by clicking on “select table“. Use the CTRL key to select multiple tables. After clicking “select table“ the table columns (= attributes) are displayed.

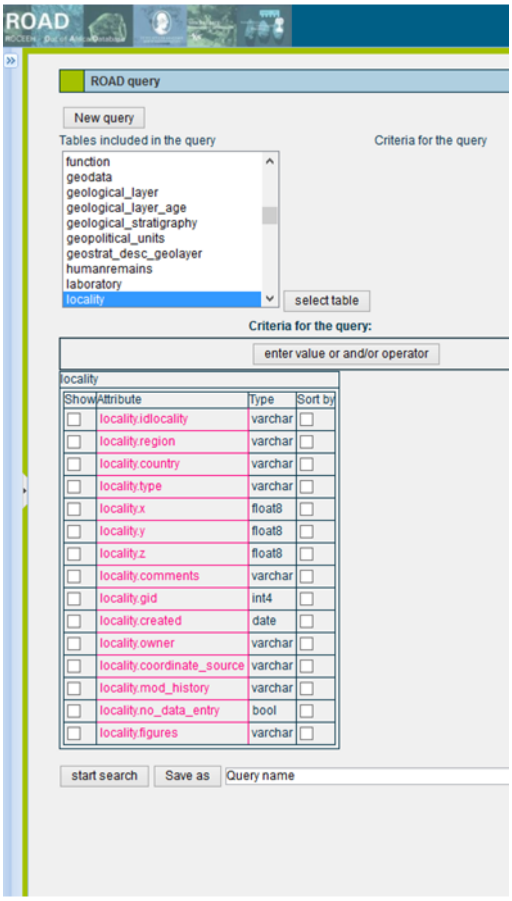

SELECT and ORDER BY clauses

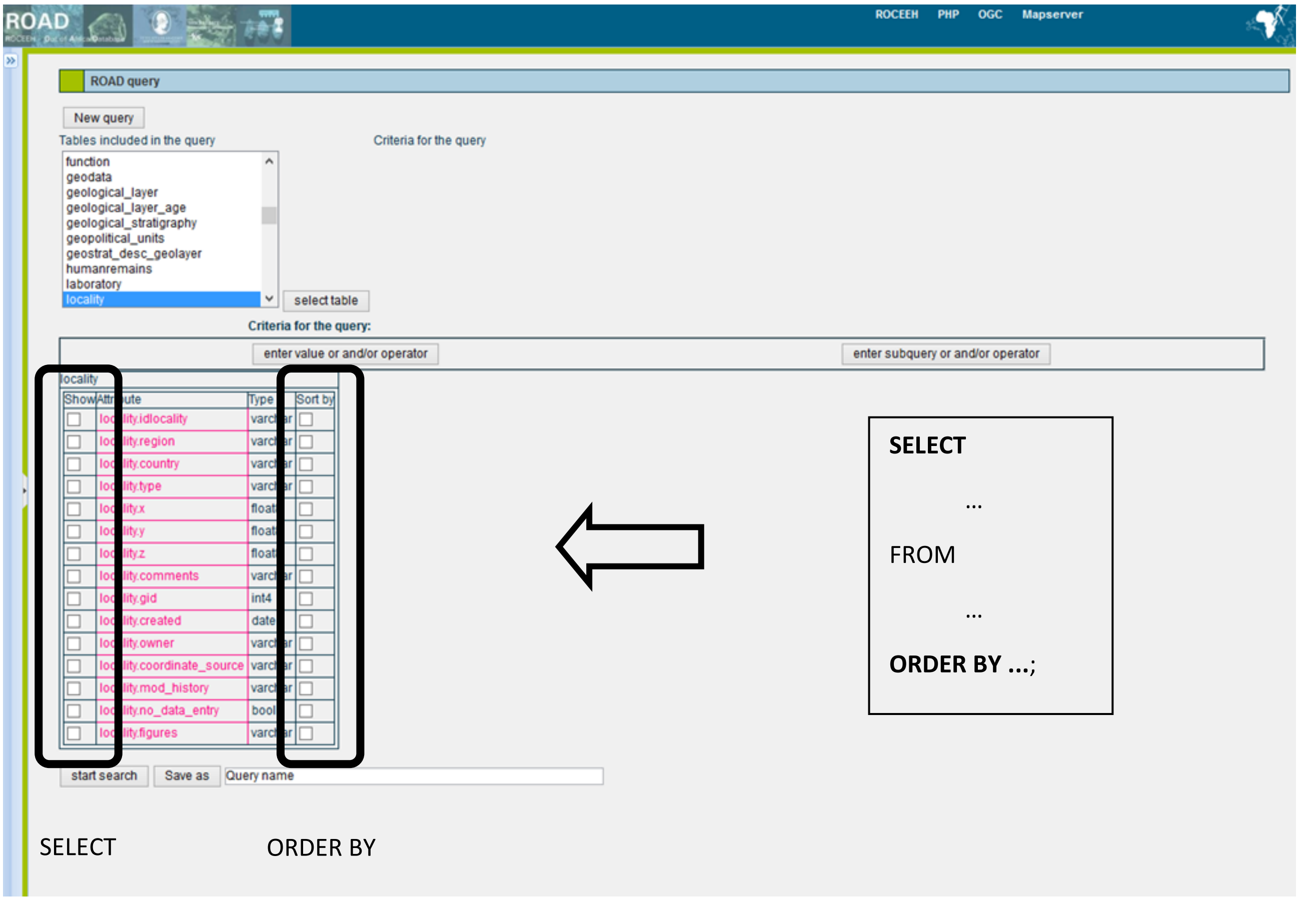

Next, the SELECT and ORDER BY clauses can be specified. Select each column to be displayed in the search results by clicking the checkbox on the left hand side. The checkbox on the right hand side corresponds to the ORDER BY clause. Selection of one sorting criterion is possible, but not required. Do not select more than one criterion from all tables in the query. The ORDER BY clause in the ROADWeb query tool does not imply a hierarchy, and so the query results won't be displayed.

WHERE clause

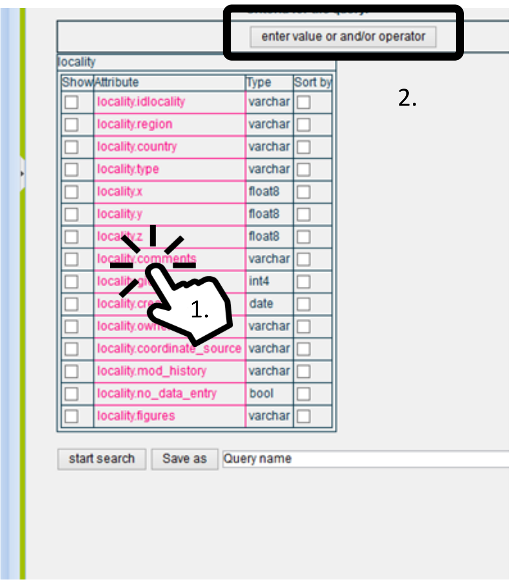

In order to create a condition in the WHERE clause, the user needs to perform two clicks:

1. Click on the column name (attribute in pink) and
2. then click on the button "enter value OR and/or OPERATOR".

The selection appears in the upper part of the window.

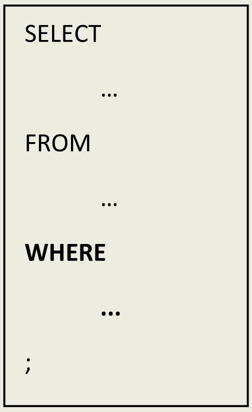

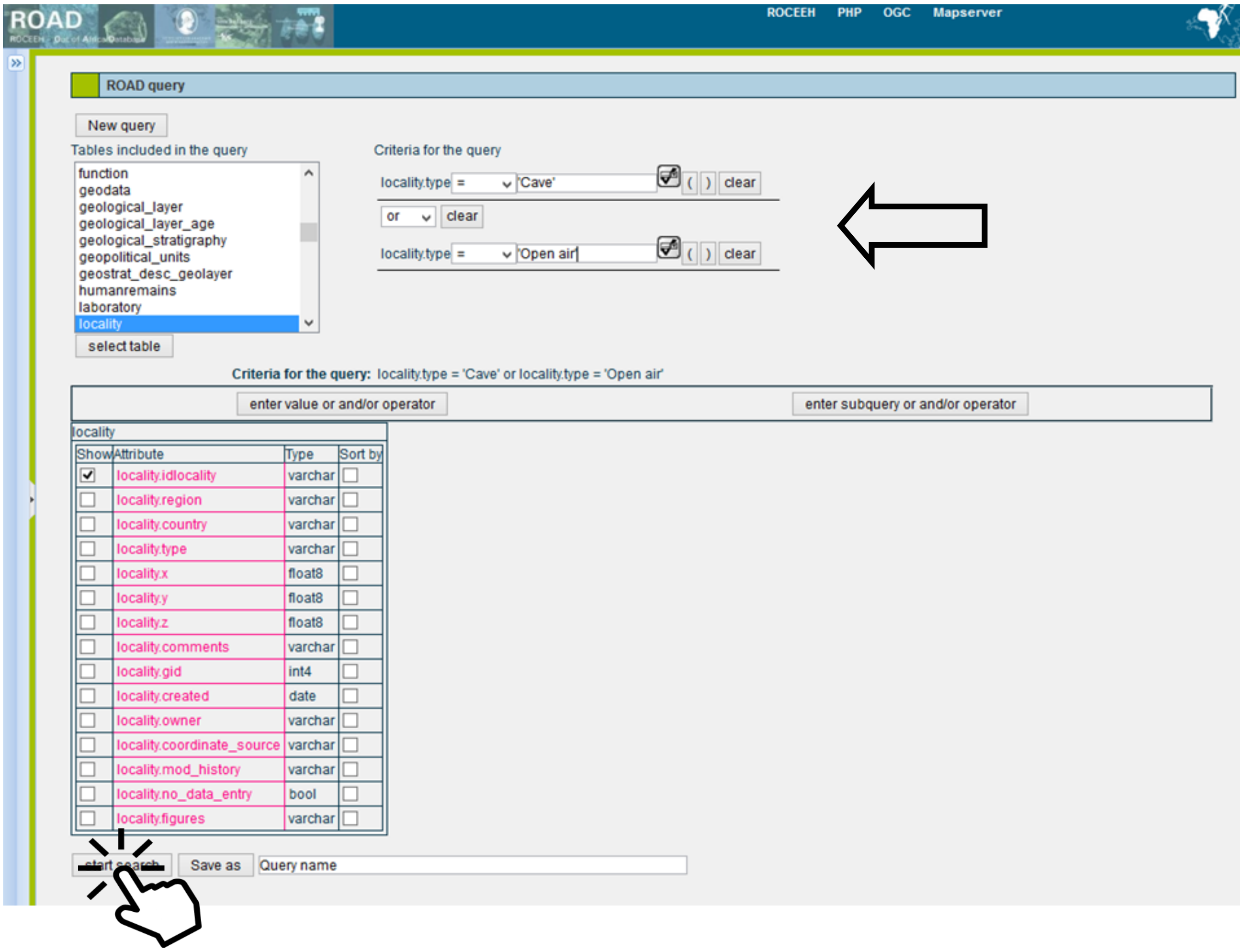

Now the comparison operator and the value to compare may be specified. When you design a query please keep in mind that sequence matters. The sequence of your criteria has a significant impact on your results. Particularly, in case of a complex query it may be good to structure your query before you start to compose it in the ROADWeb query tool.

Before you run the query by clicking on the button in the lower left, check the following items:

- Did you choose attributes to be displayed in the query results?
- Did you choose a single sorting criterion?
- Did you delete the last operator in your query?

If you answered all questions with ‘yes‘, then click “start search“ to run your query. The results will be displayed in a new window.

A last word on saving your query: ROAD offers two options, first, to save your query as such or, second, to save the results of your query. Before you save, think about what serves your purpose best. If you want to keep your query, maybe only changing some of the criteria in the WHERE clause next time, you may do this on the page shown above with the “save as“ button. Think about a clear name that allows you to find it again in the list. If you want to display the results of your query, for instance on a map, or if you wish to edit the results table of your query—maybe because it contains equivocal datasets—it might be better to save your results instead of the query structure. After you run the query, you will see a corresponding button under the results table. To save the data in an Excel table (csv format).

Understood so far? Great! That means you’re ready for another round of exercises.

Exercise Q-II

ROAD also includes a table called ‘locality‘, which is in fact the most important tables in the database.

1. **Write queries 1 and 2 from Exercise Q-I with the ROADWeb query tool. Note: values of the attribute ‘type‘ use lower case letters.**

> **EXCERCISE Q-I:**
>
> 1. **Write a query to (i) find all localities of type ‘Open air‘ or ‘Cave‘ and (ii) order the query results by type.**
> 2. **Write a query: find all localities in South Africa with ‘Cave‘ as a part of idlocality by using the ‘like‘ family of operators.**

2. **Find out with one query how many different locality types are stored in ROAD.**

3. **Find out with two queries if the number of all different combinations of genus and species stored in the table „publication_desc_humanremains“ is greater than the number of all different combinations of locality, assemblage, genus and species stored in the table „publication_desc_humanremains“**

(*) The correct results of the exercises are shown at the end of this manual.

Joins

Until now we worked exclusively with a single table—the locality table. Most databases, however, possess more than a single table. If a database has more than one table, we may wish to join two (or more) of them to retrieve the information we need. SELECT statements that work on two more tables are called Joins.

Join combines table entries from two or more tables—row by row and column by column. Let us take two examples, table 1 (blue) and table 2 (red):

col1_1	col1_2
1	bcd
4	efg
7	higklmno

```
SELECT
    *
FROM
    exampleTable1,
    exampleTable2
;
```

col2_1	col2_2	col2_3
1	a	bcd
4	b	qrst

Coll1_1	Coll1_2	Coll2_1	Coll2_2	Coll2_3
1	bcd	1	a	bcd
1	bcd	4	b	qrst
4	efg	1	a	bcd
4	efg	4	b	qrst
7	higklmno	1	a	bcd
7	higklmno	4	b	qrst

If we do not specify which fields are being joined, the result is called a "Cartesian join", in which all rows in the blue table are joined with all rows in the red table. This procedure corresponds to a multiplication of the table rows (3 x 2 = 6), wherein the table columns are unified. In practice, Cartesian joins make no sense. They are resource intensive, and they are usually performed by accident. They are, however, important for understanding how joins work in general. In order to establish joins between two tables we need to specify in the WHERE clause which attributes correspond to each other. Have a look at the example below which also joins both of our tables. This time the two joins are linked using the operator AND. Both conditions must be fulfilled. In this case, only a single case remains instead of the six resulting from a Cartesian join.

```
SELECT
    *
FROM
    exampleTable1,
    exampleTable2
WHERE
    col1_1 = col2_1
AND
    col1_2 = col2_3
;
```

Coll1_1	Coll1_2	Coll2_1	Coll2_2	Coll2_3
1	bcd	1	a	bcd

Only the first row in the table below meets the requirements of the join. In the case of joins #2, 3, 5, and 6, neither one of the join conditions are met. In join #4, the first condition is met, but not the second one. If tables are linked with joins, all conditions must be fulfilled.

Coll1_1	Coll1_2	Coll2_1	Coll2_2	Coll2_3	
1	bcd	1	a	bcd	✓
1	bcd	4	b	qrst	1 ≠ 4 and<br>bcd ≠ qrst
4	efg	1	a	bcd	4 ≠ 1 and<br>efg ≠ bcd
4	efg	4	b	qrst	efg ≠ qrst
7	higklmno	1	a	bcd	7 ≠ 1 and<br>h… ≠ bcd
7	higklmno	4	b	qrst	7 ≠ 4 and<br>h… ≠ qrst

Tables ‘locality‘ and ‘assemblage‘

In addition to the locality table (introduced at the beginning of the query section) we show another table, the table ‘assemblage‘. The assemblage table contains specific information about names and categories which are not mentioned in the locality table. The attributes ‘idlocality’ and ‘idassemblage’ represent unique identifiers and/or primary keys of the table assemblage. Let us first join the information on localities and their assemblages.

Table: Assemblage

idlocality	idassemblage	name	category
Sibudu Cave	1	106-1	plant remains
Sibudu Cave	2	B/Gmix, B/Gmix2 Inven-tar	feature, raw material
Blombos Cave	1	BBCM2	human remains
Haalenberg	1	Haalenberg Fauna 6	paleofauna
Haalenberg	2	MSA Inventar	raw material, miscellaneous finds, technology, typology
Swartkrans	1	Swartkrans Member 1 Fauna	paleofauna
Swartkrans	2	Member 3	organic tools, raw material, technology, typology
Swartkrans	3	Swartkrans Member 1	human remains
Tiras 5	1	MSA Inventar	raw material, technology, typology

```
SELECT
    *
FROM
    assemblage AS a
    locality AS b
WHERE
    a.locality = b.locality
```

This join has matching idlocality columns and relates meaningful data of the tables locality and assemblage. With this join we retrieve related information about assemblages and their respective localities.

Question: What happens if we eliminate the WHERE clause in the SELECT statement? How many records will appear in the result table?

Note that the locality Linksfield does not occur in the result table. It is included in the locality table, but not in the table assemblage. The SELECT statement moreover introduces the use of aliases for the names of tables. Aliases are introduced in the FROM clause by relating the name of a table with AS followed by the corresponding alias. Aliases may always be used. They help to identify a particular column and avoid ambiguities. In this case, ambiguity is caused by the column idlocality, which occurs two times in the result table, one time originating from the assemblage table, and another time from the locality table. Distinguishing between the idlocality columns is required. This is done by specifying the aliases in the column names.

Table: Assemblage | **Table: Locality**

idlocality	idassemblage	name	category	idlocality	country	type	x	y
Sibudu Cave	1	106-1	plant remains	Sibudu Cave	South Africa	Rock shelter	31.086	-29.522
Sibudu Cave	2	B/Gmix, B/Gmix2 Inventar	feature, raw material	Sibudu Cave	South Africa	Rock shelter	31.086	-29.522
Blombos Cave	1	BBCM2	human remains	Blombos Cave	South Africa	Cave	21.218	-34.413
Haalenberg	1	Haalenberg Fauna 6	paleofauna	Haalenberg	Namibia	Rock shelter	15.467	-26.586
Haalenberg	2	MSA Inventar	raw material, miscellaneous finds, technology, typology	Haalenberg	Namibia	Rock shelter	15.467	-26.586
Swartkrans	1	Swartkrans Member 1 Fauna	paleofauna	Swartkrans	South Africa	Cave	27.755	-25.969
Swartkrans	2	Member 3	organic tools, raw material, technology, typology	Swartkrans	South Africa	Cave	27.755	-25.969
Swartkrans	3	Swartkrans Member 1	human remains	Swartkrans	South Africa	Cave	27.755	-25.969
Tiras 5	1	MSA Inventar	raw material, technology, typology	Tiras 5	Namibia	Rock shelter	16.583	-26.208

```
SELECT
	*
FROM
	assemblage AS a
	locality AS b
WHERE
	a.locality = b.locality
AND
	category LIKE '%human remains%';
```

In addition to join conditions, the Join statements may have other conditions in their WHERE clause. Here we see a WHERE clause consisting of one join condition and another condition for column category combined with the AND operator.

Join conditions and other conditions have to be combined with the AND operator. If further conditions should be met, then brackets should be used in the WHERE clause.

Question: What happens if we replace the AND operator in the WHERE clause with the OR operator?

Table: Assemblage **Table: Locality**

idlocality	idassemblage	name	category	idlocality	country	type	x	y
Sibudu Cave	1	106-1	plant remains	Sibudu Cave	South Africa	Rock shelter	31.086	-29.522
Sibudu Cave	2	B/Gmix, B/Gmix2 Inventar	feature, raw material	Sibudu Cave	South Africa	Rock shelter	31.086	-29.522
Blombos Cave	1	BBCM2	human remains	Blombos Cave	South Africa	Cave	21.218	-34.413
Haalenberg	1	Haalenberg Fauna 6	paleofauna	Haalenberg	Namibia	Rock shelter	15.467	-26.586
Haalenberg	2	MSA Inventar	raw material, miscellaneous finds, technology, typology	Haalenberg	Namibia	Rock shelter	15.467	-26.586
Swartkrans	1	Swartkrans Member 1 Fauna	paleofauna	Swartkrans	South Africa	Cave	27.755	-25.969
Swartkrans	2	Member 3	organic tools, raw material, technology, typology	Swartkrans	South Africa	Cave	27.755	-25.969
Swartkrans	3	Swartkrans Member 1	human remains	Swartkrans	South Africa	Cave	27.755	-25.969
Tiras 5	1	MSA Inventar	raw material, technology, typology	Tiras 5	Namibia	Rock shelter	16.583	-26.208

```
SELECT
        category, country
FROM
        assemblage
        locality
WHERE
        assemblage.locality = locality.locality
AND
        category LIKE ‚%human remains%‘;
```

In this SELECT statement we have the same join condition, but without aliases a and b. To avoid ambiguity, we insert the table names instead of the aliases. From this SELECT statement we retrieve a result table with two rows and two columns.

**Table: Assemblage**	**Table: Locality**
**category**	**country**
human remains	South Africa
human remains	South Africa

Exercise Q-III

1. Show the result of the following SQL statement:

```
SELECT
    *
FROM
    exampleTable1,
    exampleTable2
WHERE
    col1_1=col2_1;
```

Coll1_1	Coll1_2	Coll2_1	Coll2_2	Coll2_3

2. What is the difference in the SELECT statement below and how many search results will we get?

```
SELECT
    *
FROM
    exampleTable1,
    exampleTable2
WHERE
    col1_1 = col2_1
OR
    col1_2 = col2_3;
```

3. Write a query: find all localities in Namibia with paleofauna assemblages (in category).

4. Write a query: find all localities of type ‘Cave‘ with stone artifacts as assemblages. Stone artifact categories are typology, technology or raw material.

(*) The correct results of the exercises are shown at the end of this manual.

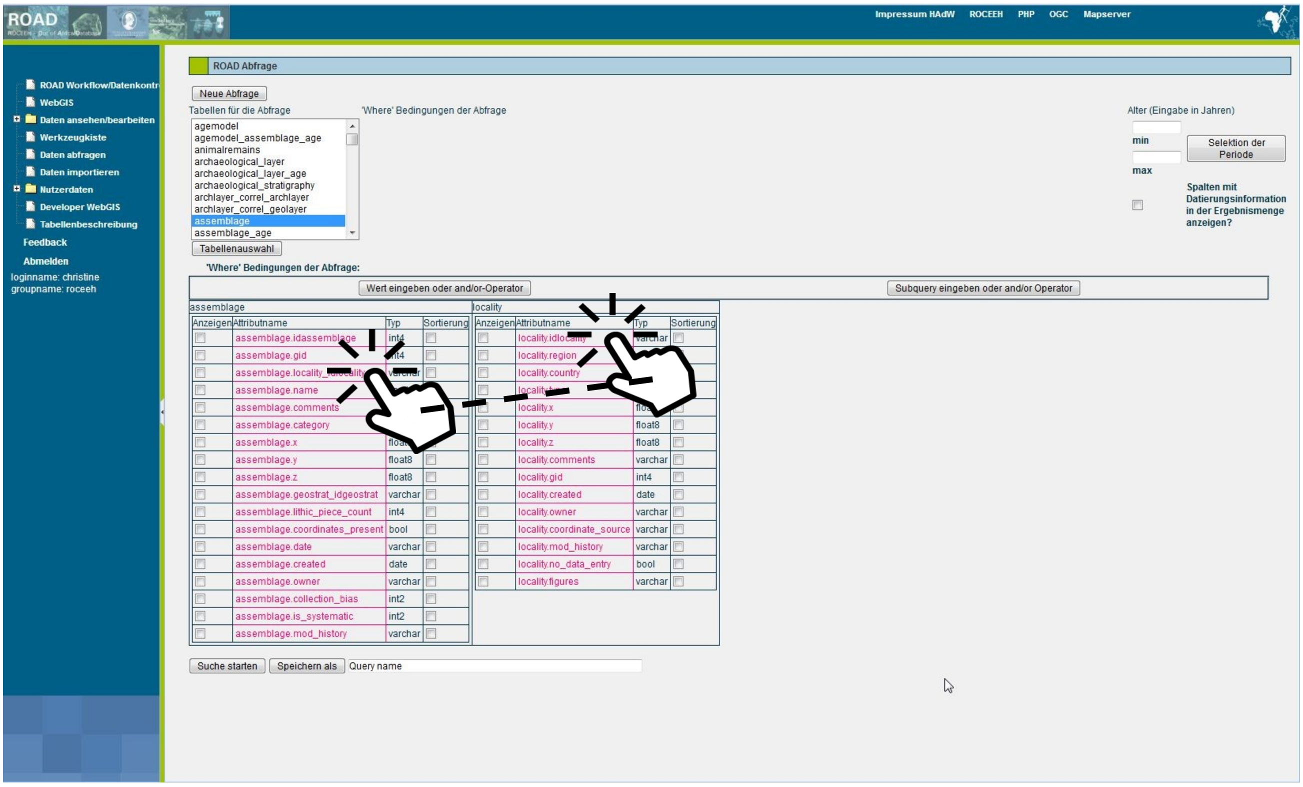

In ROADWeb join conditions are established, by selecting the tables required for a particular query. In the next step, the two tables are linked by clicking on the attributes which correspond to each other. As soon as a second attribute is selected, the join appears in the upper part of the window linked by an " = " sign.

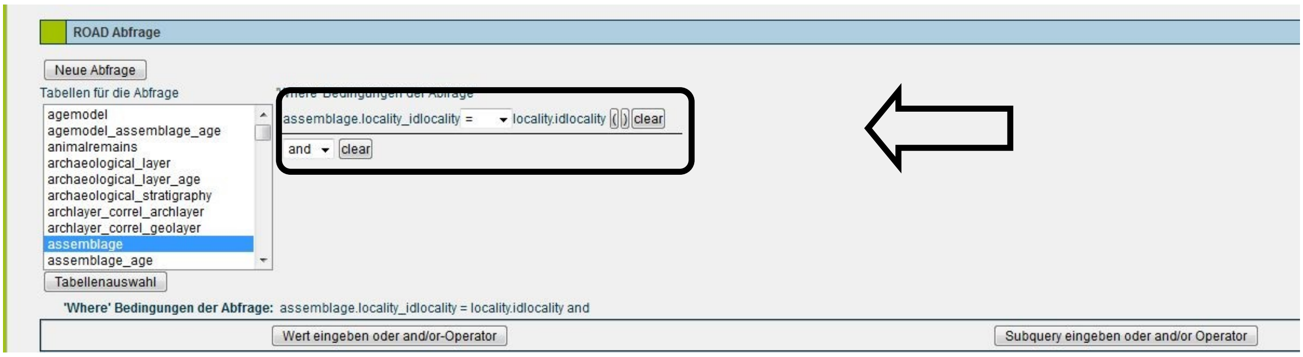

Please keep in mind that sequence matters, and this is also valid for joins. Should you wish to design a rather complex query, please sketch it on paper first and try to figure out the proper sequence of joins.

You may either save your query (to continue working on it later) or you may run the query and save the results (for instance if you wish to plot the results on a map.)

Query checklist:

- First add joins, then add WHERE clauses
- Link joins and other WHERE conditions using AND
- All brackets in place?
- Attributes chosen to be displayed in the results?
- Only one sorting criterion
- Delete last operator

Exercise Q-IV

1. Write queries 3 and 4 from Exercise Q-III using the ROADWeb query tool.

EXCERCISE Q-III:

3. Write a query: find all localities in Namibia with paleofauna assemblages.

4. Write a query: find all localities of type 'Cave' with stone artifacts as assemblages. Stone artifact categories are typology, technology or raw material.

2. Write a query to find out the classification (genus, species) of skeletal elements stored in human remains.
Note: This query requires information stored in further tables in addition to assemblage and locality. Please sketch the query before you start to write it with the ROADWeb query tool.

(*) The correct results of the exercises are shown at the end of this manual.

Querying for age and dating with the ROADWeb Query Tool

Chronological information in ROAD may be associated with a variety of units, for instance a geological layer, an assemblage and/or a stratigraphic unit, be it archaeologically or geologically defined. If you wish to perform a query that includes age and/or dates, you would need to query five tables.

Geological_stratigraphy	source of dating
Geostrat_desc_geolayer	linking table
Geological_layer	source of layer information
Assemblage_in_geolayer	linking table
Assemblage	source of assemblage information

However, the primary source of information about the chronology and the age of an assemblage is the table 'geological_stratigraphy'. Geological stratigraphy incorporates the quintessential information about dates thereby providing the narrowest possible age of a layer. In other words, 'geological_stratigraphy' contains an unambiguous summary of available dating records.

Read the following query and guess the result:

```
SELECT
        assemblage.locality_idlocality
FROM
        assemblage.
        assemblage_in_geoloayer,
        geological layer,
        geological_stratigraphy,
        geostrat_desc_geolayer
WHERE
        assemblage_locality_idlocality = assemblage_in_geolayer.assemblage_idloclaity
AND
        assemblage.idassemblage = assemblage_in_geolayer.assemblage_idassemblage
AND
        assemblage_iin_geolayer.geolayer_idlocality = geological_layer.locality.locality_idlocality
AND
        assemblage_in_geolayer.geolayer_name = geological_layer.name
AND
        geological_layer.locality_idlocality = geostrat_desc_geolayer.geolayer_idlocality
AND
        geological_layer.name = geostrat_desc_geolayer.geolayer_name
AND
        geological_stratigraphy.idgeostrat = geostrat_desc_geolayer.geostrat_idgeostrat
AND
        geological_stratigraphy.age_min <= 50000
AND
        geological_stratigraphy.age_max >= 50000
```

Exercise Q-V

1. **Write the query you just studied with the ROADWeb query tool. Compose the long WHERE clause.**

2. **Modify the query to list all localities with assemblages of the age 20,000 — 50,000 years.**

(*) The correct results of the exercises are shown at the end of this manual.

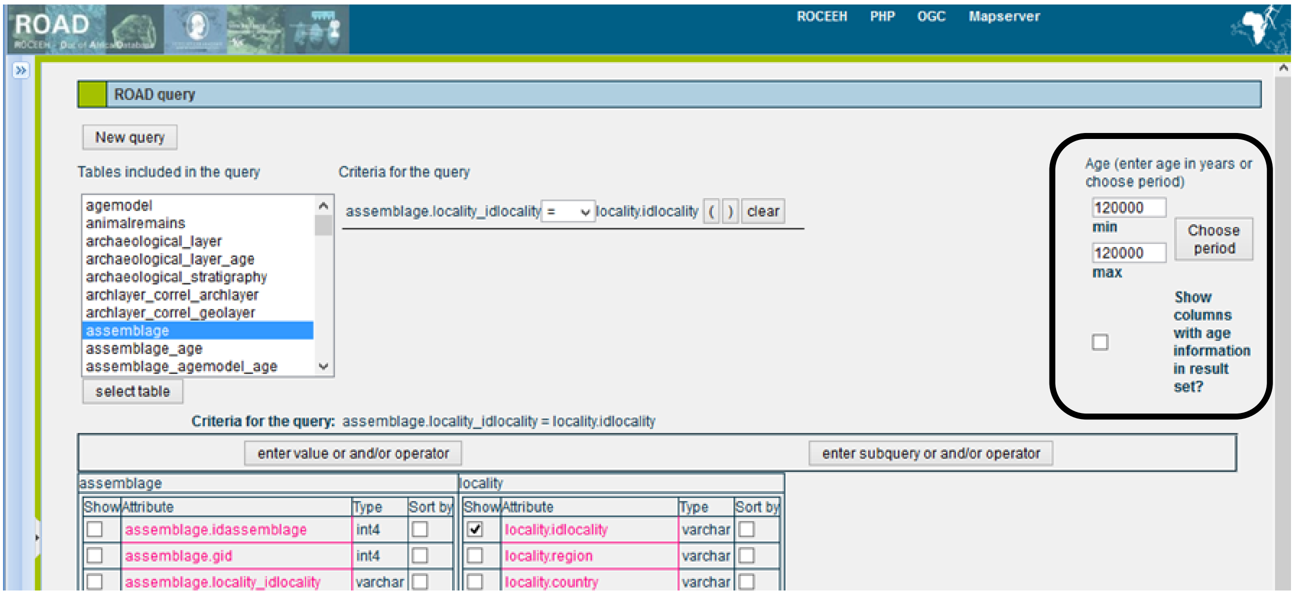

Fortunately, we developed a more convenient way to work with the ROADWeb query tool and compose the age query: the age box. The age box automatically appears in the upper right when tables associated with the assemblage table are selected. You just need to fill in a minimum and a maximum age. Entries in these cells define the 'search age interval'. Both fields must be filled out to perform the age query correctly! If one field is left blank, the results will include all assemblages regardless of age. If you wish to search for a single age, you may enter the same value in both fields. After the SQL query is performed, the generated SQL query is shown in a new window along with the search results.

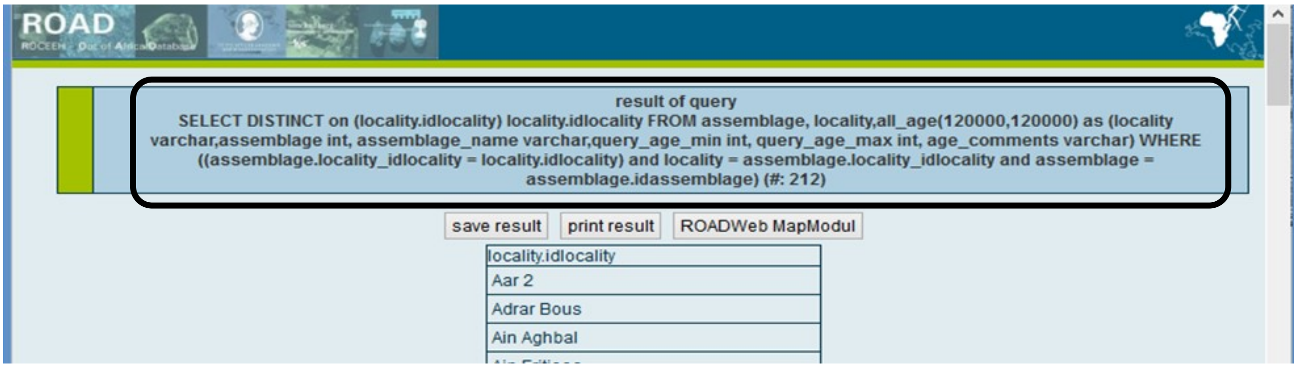

There is one more issue to be kept in mind, when searching for age intervals. Age interval search is “true“ if the queried age interval intersects with the database age interval, that is, in these four cases:

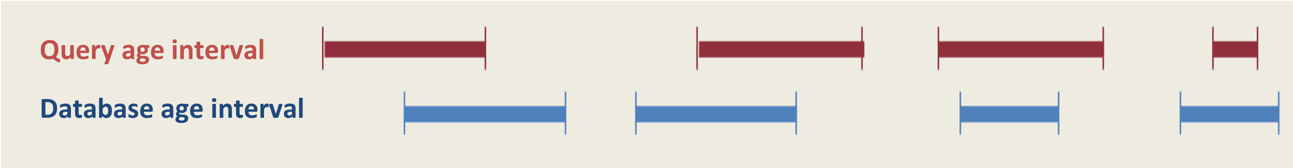

Searching for an age interval, the query results will eventually include localities and/or assemblages which are either younger or older than the queried age interval. This is because the given age interval intersects with the queried one.

If there is no overlap at all, the age interval search is “false”, as shown by the examples below:

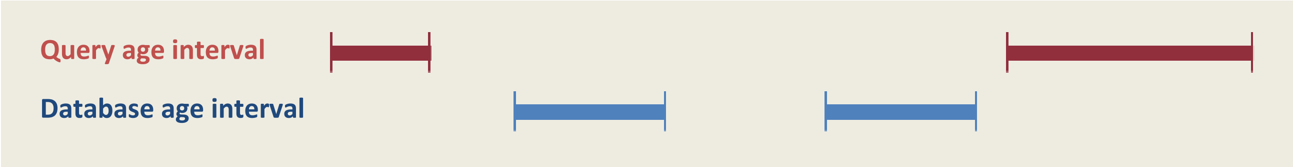

Notes

The ROAD Map Module allows the user to view various spatial information without additional knowledge of GIS software. This spatial information can be added as a layer to the given default map which already has a base layer.

There are two kinds of layers that can be added to a given map: 1) a layer with predefined content (WMS map layer), and 2) a layer with content that the user defines (search layer).

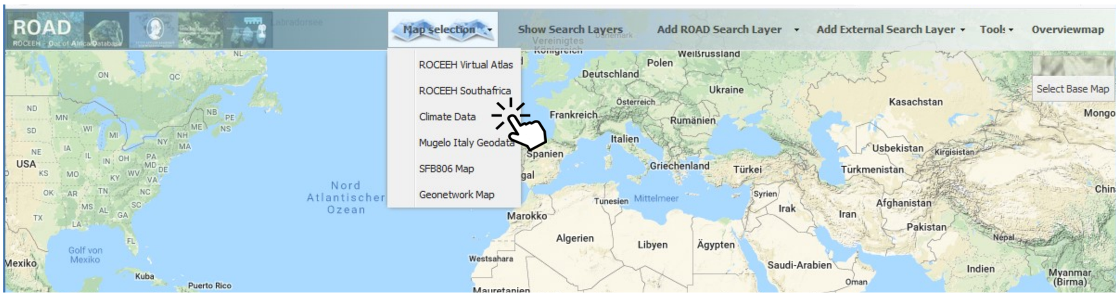

1. To add a WMS map layer, first select a WMS layer collection (WMS map). The menu item "Map selection" shows a list of available WMS maps. When a map in this list is selected, a window with layers of the selected map will be shown. To add the WMS map layer to the map, select a layer.

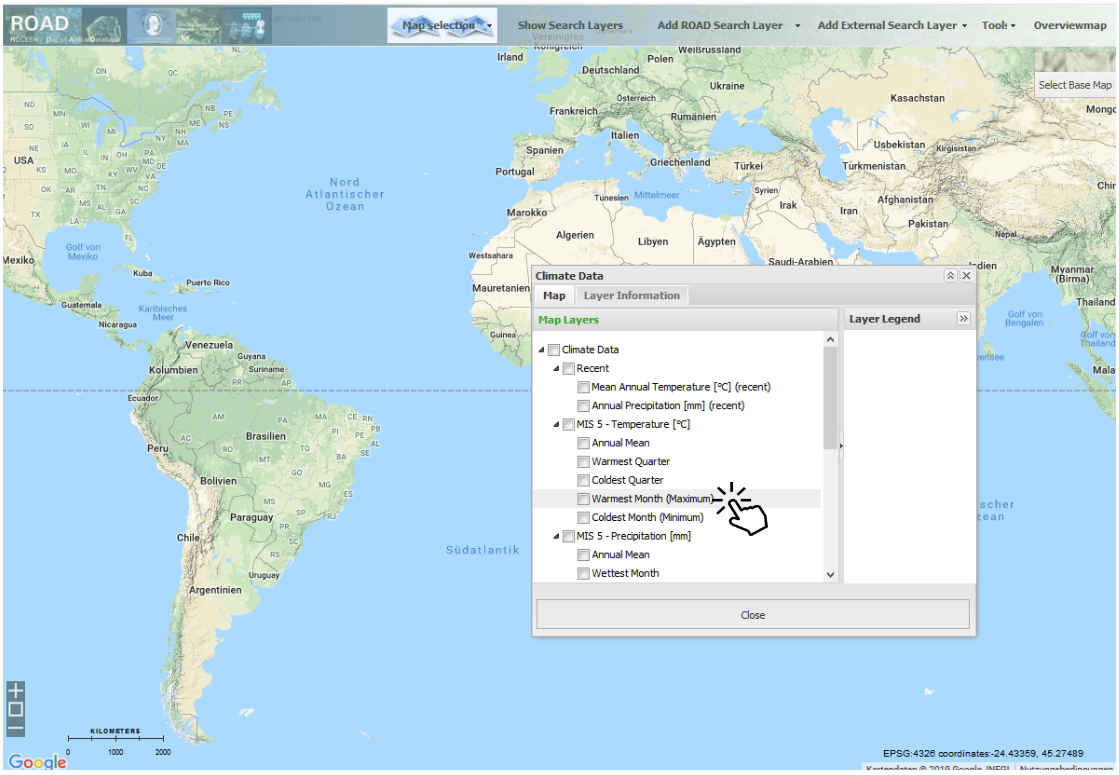

2. To add a search layer, you must first perform a search. The Map Module presently offers two search possibilities: a) search the ROAD database, and b) search a remote database.

a) The menu item "Add ROAD Search Layer" allows the user to use either a simple search template or search results already generated in a ROADWeb query. With the simple search template, the user can vary search basic parameters to perform a search in ROAD. In both cases the search results will be added as a layer to the map.

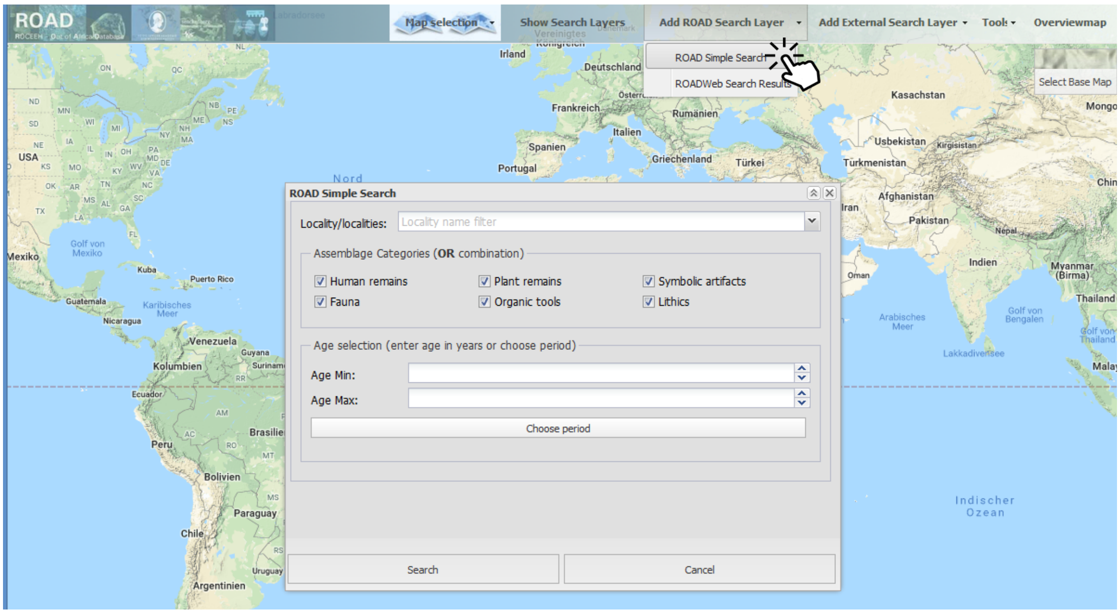

b) The menu item "Add External Search Layer" shows a list with available databases for search. The ROAD Map Module currently allows searches in two external databases: Neotoma and Neogene Quaternary Mammals Database (NQMDB). Neotoma covers the Pliocene-Quaternary part of Earth's history, during which humans evolved and modern ecosystems developed. NQMDB hosts lists of micro- and macro-mammals for Pleistocene fossil localities in western Eurasia. To get a search template, select a database from the list. The search template allows you to vary one or more search parameters and perform a search in the remote database. The search results will be added as a layer to the map.

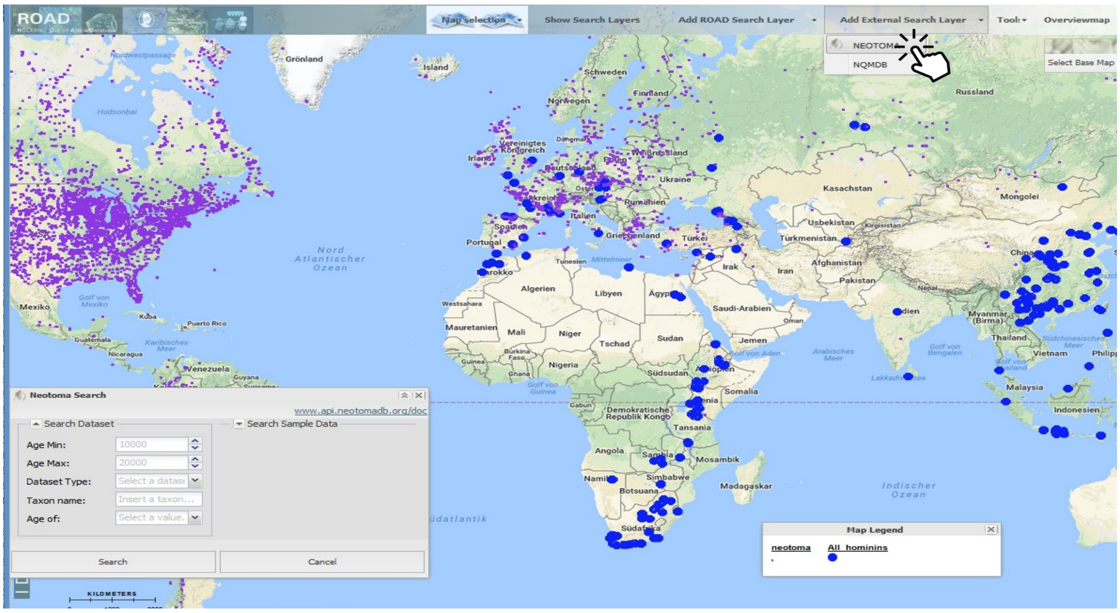

3. Each search layer of the map can be disabled or enabled by clicking on the layer in the “Search Layer Window”. In the same “Search Layer Window“ there are four buttons for each layer, which allows layer modifications. The first button changes the opacity of the layer, the second changes the symbol, and the third changes the size of the symbol.

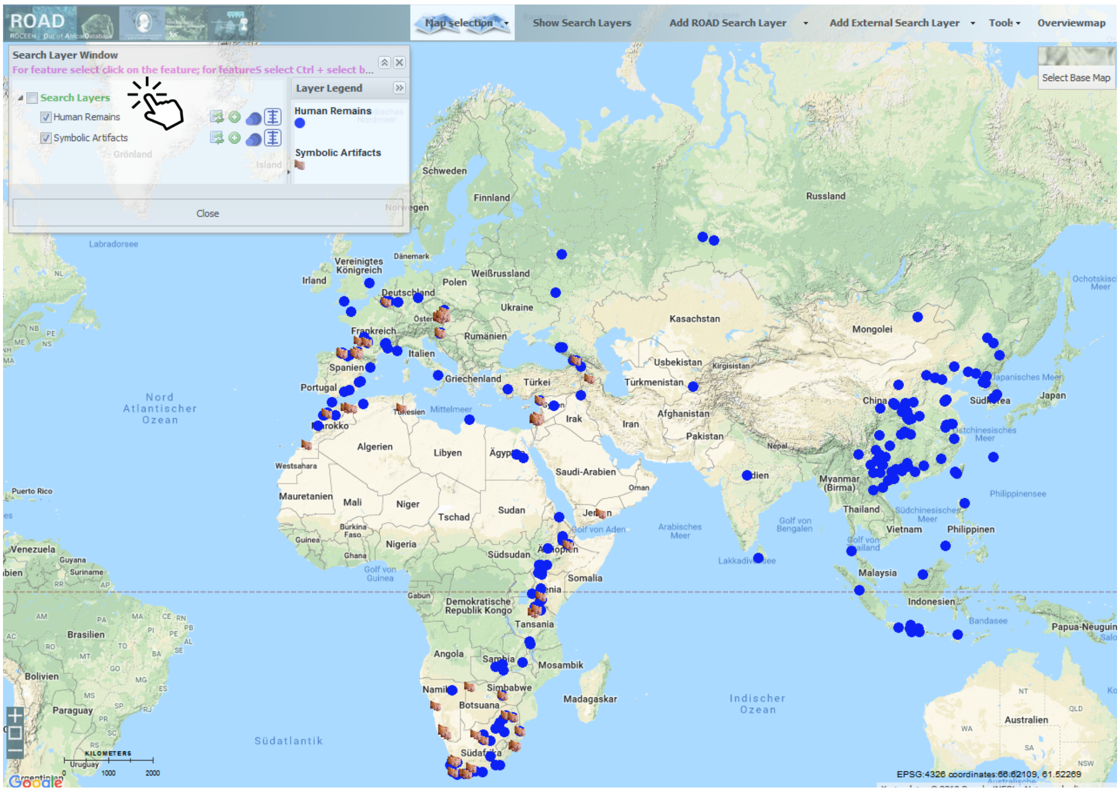

The fourth button is an experimental button that allows the user to dynamically query the age of the layer features. The slider allows you to change the time specifications for minimum and maximum age.

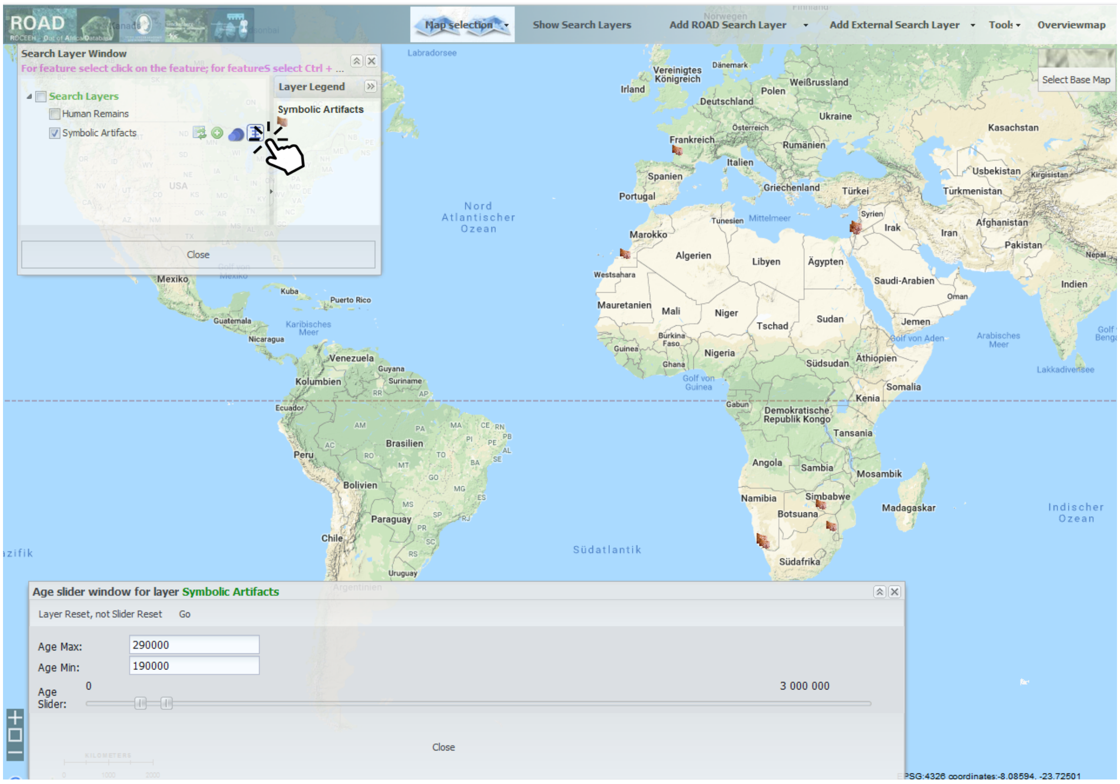

4. Each WMS layer of the map can be disabled or enabled by clicking on the layer in its WMS layer collection/map window. In the same window there is one button to change the layer opacity for each layer.

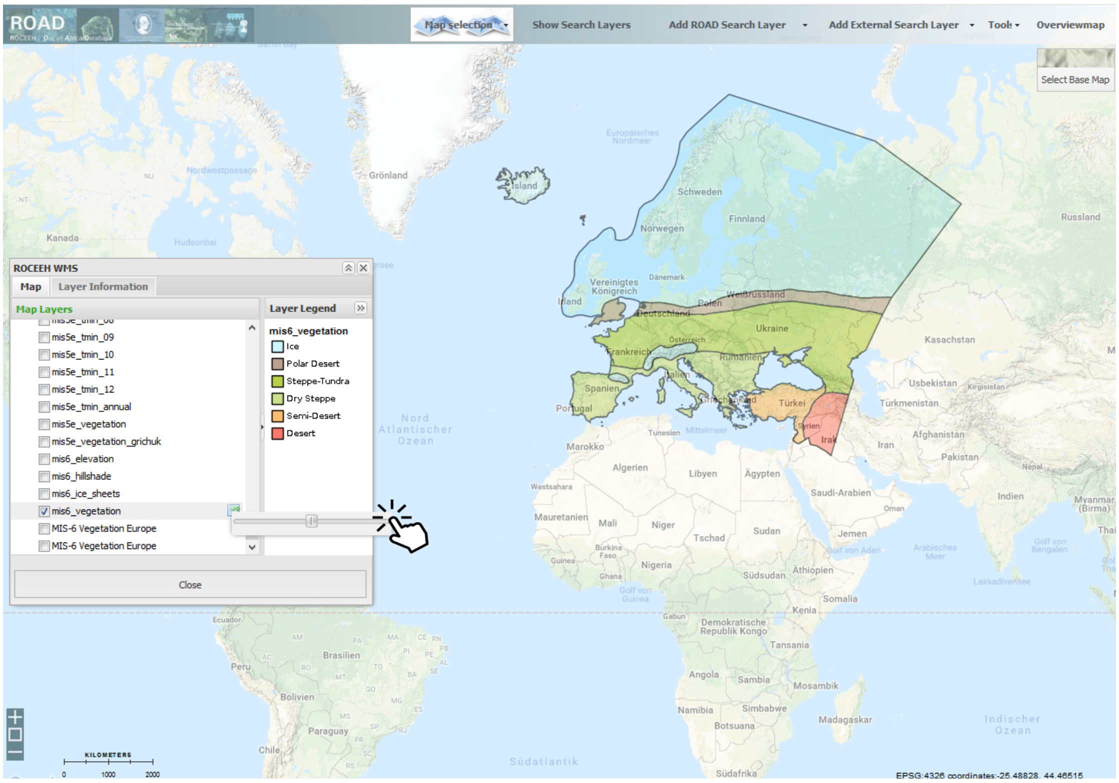

5. Legend

The legend explains all visible symbols and colors in the map. To display the whole legend of the map, select the menu item "Tools” and then “Create Map Legend Window".

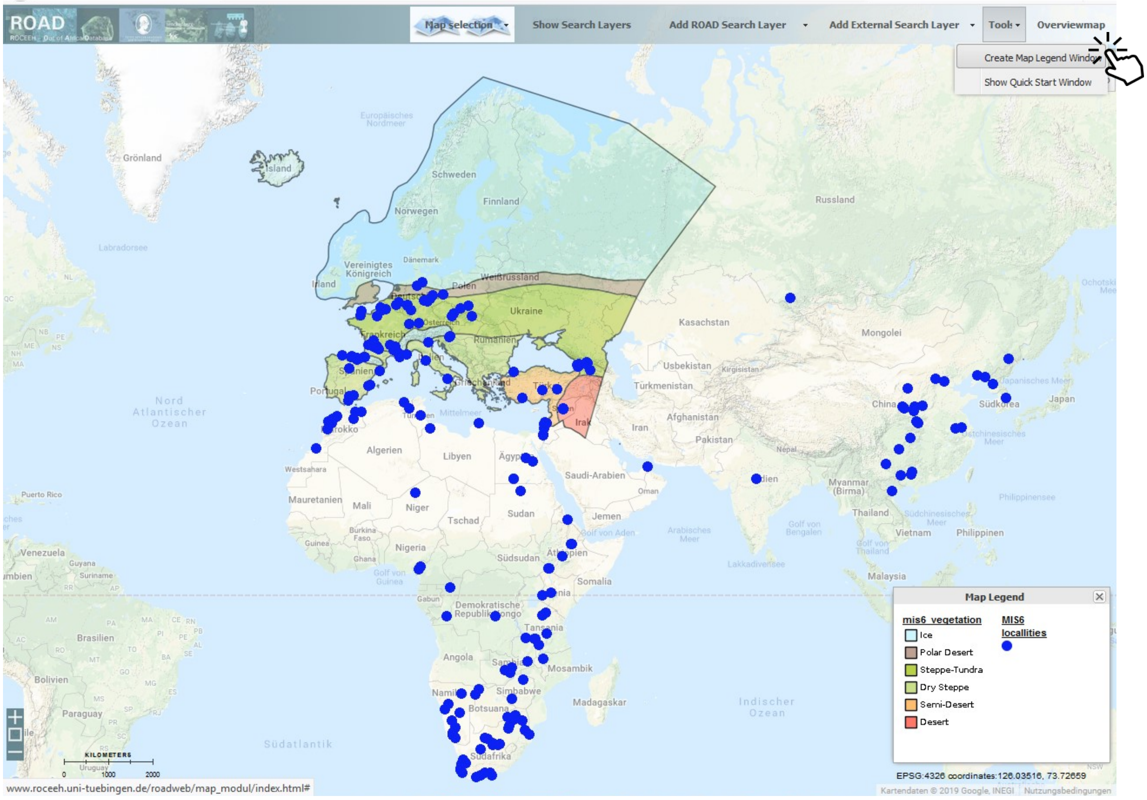

6. Reference Map

The reference map is an inset which provides an overview for the user. It displays the map extent within a red rectangle in the lower right corner of the map window. The user can activate the reference map by clicking the “Overview Map“ button in the tool bar; clicking it again deactivates this feature.

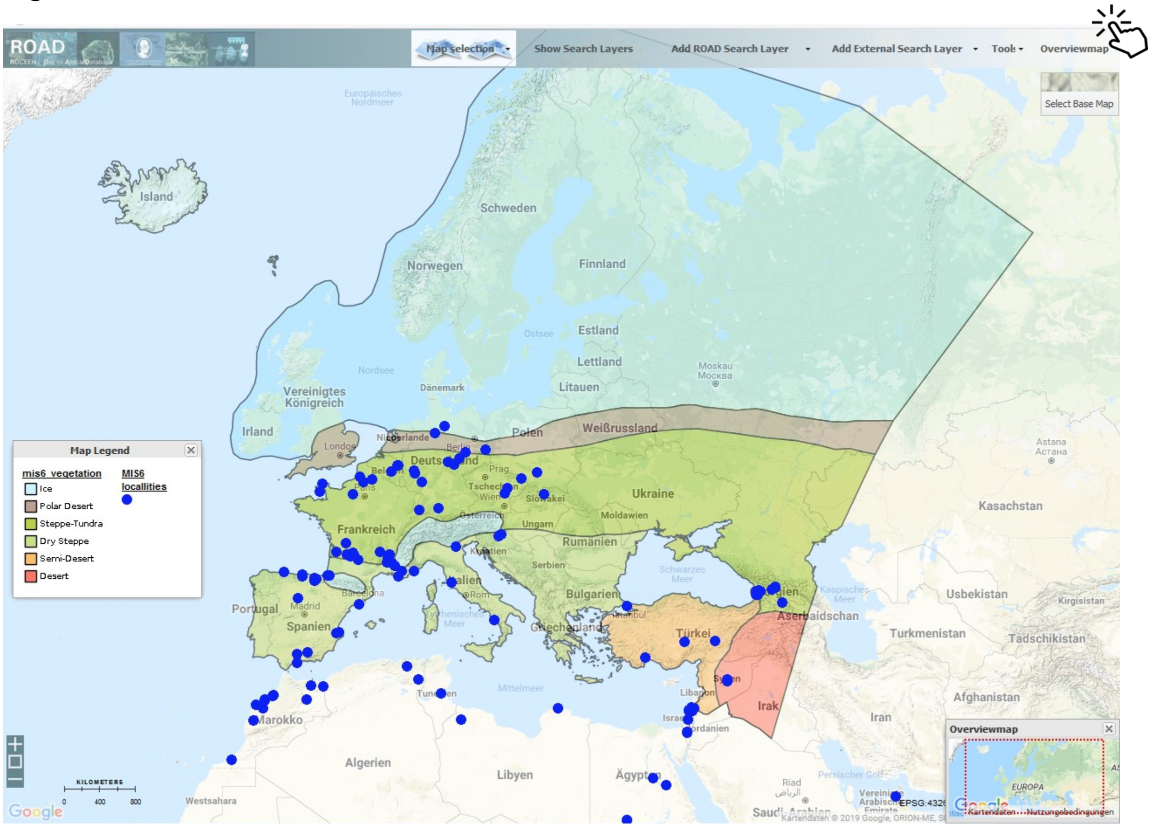

Notes

Answers

Under construction

CONTACT

The Role of Culture in Early Expansions of Humans
Heidelberg Academy of Sciences and Humanities

Senckenberg Research institute Frankfurt/Main
Eberhards Karls University of Tübingen

COORDINATORS

Miriam Haidle (scientific)
Julia Heß (administrative)

Senckenberg Research institute
Senckenberganlage 25
D-60325 Frankfurt/Main
miriam.haidle@uni-tuebingen.de
julia.hess@senckenberg.de
www.roceeh.net

THE ROLE OF CULTURE IN EARLY EXPANSIONS OF HUMANS

The Heidelberg Academy of Sciences and Humanities is a member of the Union of German Academies of Sciences and Humanities, which coordinates the Academies' Program. The research project, "The Role of Culture in Early Expansions of Humans," was incorporated into the Academies' Program in 2008.

April 2019
