# Supplementary material for: The ROCEEH Out of Africa Database (ROAD): A large-scale research database serves as an indispensable tool for human evolutionary studies
Source: PLoS One. 2023 Aug 1;18(8):e0289513. doi: 10.1371/journal.pone.0289513 (PMC10393170; doi:10.1371/journal.pone.0289513)
Supplement: S3 File — This document specifies the responsibilities of a user and serves as an application to set up a user account. (PDF) [file pone.0289513.s005.pdf]

ROCEEH Out of Africa Database (ROAD)

Data Use Policy

The research center "The Role of Culture in Early Expansions of Humans" (ROCEEH, www.roceeh.org), provides data available through the ROCEEH Out of Africa Database (ROAD) free of charge and under the open license CC BY-SA 4.0.

Accordingly, you are free to

- Share — copy and redistribute the material in any medium or format
- Adapt — remix, transform, and build upon the material for any purpose, even commercially.

Under the following conditions

- Attribution — You must give appropriate credit, provide a link to the license, and indicate if changes were made. You may do so in any reasonable manner, but not in any way that suggests the licensor endorses you or your use
- ShareAlike — If you remix, transform, or build upon the material, you must distribute your contributions under the same license as the original
- No additional restrictions — You may not apply legal terms or technological measures that legally restrict others from doing anything the license permits

An example for an appropriate citation is “CC BY-SA 4.0 ROCEEH Out Of Africa Database (ROAD)” or the following text in the acknowledgments: “Data were obtained from the ROCEEH Out of Africa Database (ROAD) (http://www.roceeh.org), and the work of the data contributors and ROAD community is gratefully acknowledged.”

Furthermore, users are not required, but should consider inviting a contributor of data as a co-author following the common rules of good scientific practice:

- If a user makes significant use of any individual contributor's data
- If any individual contributor's data comprises a substantial portion of a larger dataset analyzed
- If a contributor makes a significant contribution to the analysis of the data, or to the interpretation of the results.

We would appreciate it if you would send us a copy of the published work, or a link to the electronic resource. Your assistance helps the ROCEEH Team to document the usage of the ROAD database. With your signature you acknowledge the above mentioned terms and conditions of using ROAD.

Name (in block letters)

Date & place　　　　　　　　　　　　Signature

Please register me as a user of the ROCEEH Out of Africa Database (ROAD) and provide me with login data. ROCEEH will send you a password for ROAD after you complete and return this user registration form:

Title:

Name:

Surname:

Institution name:

Address:

Email:

Phone:

(For ROCEEH internal use only)

Access created:

Date:

Signature:
