# Supplementary material for: The ROCEEH Out of Africa Database (ROAD): A large-scale research database serves as an indispensable tool for human evolutionary studies
Source: PLoS One. 2023 Aug 1;18(8):e0289513. doi: 10.1371/journal.pone.0289513 (PMC10393170; doi:10.1371/journal.pone.0289513)
Supplement: S4 File — This feature makes important information about a locality available through the click of a button. The resulting PDF, for example, for Aghitu-3 Cave in Armenia demonstrates the utility of this function. (PDF) [file pone.0289513.s006.pdf]

ROAD Summary Data Sheet

for locality

Aghitu-3 Cave

The project "The Role of Culture in Early Expansions of Humans" (ROCEEH) is administered by the Heidelberg Academy of Sciences and Humanities. It has been promoted by the Joint Science Conference of the Federal Government and the governments of the states of the Federal Republic of Germany in the Academies' Programme. Funding is provided by the Federal Government of Germany (Federal Ministry of Education, Science and Research) and the states of Baden-Wurttemberg (Ministry of Science, Research and the Arts) and Hesse (Ministry of Science and the Arts).

Provided by ROCEEH 2023
under
CC BY-SA 4.0

THE ROLE OF CULTURE IN EARLY EXPANSIONS OF HUMANS

EBERHARD KARLS UNIVERSITÄT TÜBINGEN

SENCKENBERG world of biodiversity

HEIDELBERGER AKADEMIE DER WISSENSCHAFTEN
Akademie der Wissenschaften des Landes Baden-Württemberg

General Information

(locality first entered on 16th August, 2016)

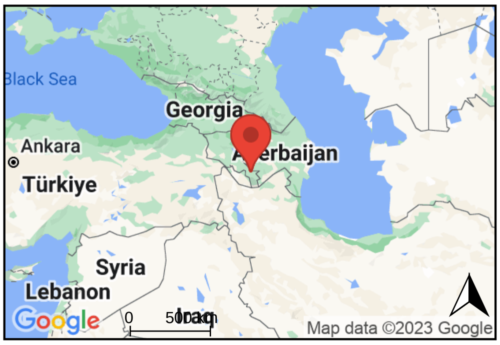

Name:	Aghitu-3 Cave
Synonyms:	No synonyms available
Country:	Armenia
Region:	Syunik
Coordinates:	39.51 N, 46.08 E
Type:	cave
Summary:	Aghitu-3 Cave is located at the base of a basalt outcrop along the Vorotan River valley. This UP site yields the earliest evidence for modern human behavior in Armenia dated between 39-24 ka cal. Notable finds include shell beads, an eyed bone needle and two more bone tools, plus many laminar stone tools made from obsidian sourced up to 250 km away and regional chert varieties. Subsistence focused on hunting of wild sheep/goat and horses.

Assemblages:
(for legend see last page)

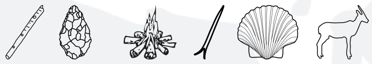

Age:

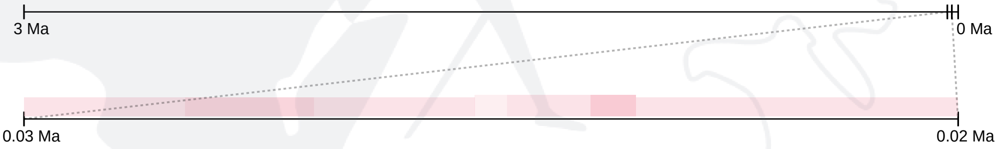

Geological Profiles

Profile(s): Main

GH 1
silt
Min Age 0
Max Age: 2000
thickness: 0.10-0.15 m

GH 2
silt
Min Age 0
Max Age: 2000
thickness: 0.10-0.20 m

GH 3
silt
Min Age 20000
Max Age: 25000
thickness: 0.5-1 m

GH 4
silt
Min Age 25000
Max Age: 25700
thickness: 0.15-0.30 m

GH 5
silt
Min Age 25000
Max Age: 25700
thickness: 0.10-0.20 m

GH 6
silt
Min Age 25000
Max Age: 25700
thickness: 0.05-0.10 m

GH 7
silt
Min Age 25000
Max Age: 25700
thickness: 0.20-0.25 m

GH 8
silt
Min Age 25700
Max Age: 27000
thickness: 0.20-0.30 m

GH 9
boulder
Min Age 27000
Max Age: 27500
thickness: 0.5-1.5 m

↓

GH 10
silt
Min Age 27500
Max Age: 32000
thickness: 2.0-2.5 m

GH 11
sand
Min Age 30000
Max Age: 34500
thickness: 1.0-1.5 m

GH 12
sand
Min Age 126000
Max Age: 300000
thickness: 0.20 m

Archeological Profiles

Profile(s): Main

AH I
historic
thickness: 0.1-0.2 m

AH II
historic
thickness: 0.1-0.2 m

AH III
upper paleolithic - eurasia
thickness: 0.5-1 m

AH IV
upper paleolithic - eurasia
thickness: 0.55-1 m

AH V
upper paleolithic - eurasia
thickness: 0.2-0.3 m

AH VI
upper paleolithic - eurasia
thickness: 2-2.5 m

AH VII
upper paleolithic - eurasia
thickness: 1-1.5 m

Assemblages

(last assemblage entered on 27th January, 2020)

Archeological Stratigraphy	Min Age	Max Age	Archeological layer	Geological layer	Assemblage	Category
Upper Paleolithic - Eurasia	20000	25000	AH III	GH 3	AH III assemblage	feature, miscellaneous finds, organic tools, raw material, symbolic artifacts, technology, typology
-	20000	25000	-	GH 3	Aghitu-3 Cave Fauna Layer AH III	animal remains, paleofauna
Upper Paleolithic - Eurasia	25000	25700	AH IV	GH 4	AH IV assemblage	raw material, technology, typology
-	25000	25700	-	GH 4	Aghitu-3 Cave Fauna Layer AH IV	animal remains, paleofauna
Upper Paleolithic - Eurasia	25000	25700	AH IV	GH 5	AH IV assemblage	raw material, technology, typology
-	25000	25700	-	GH 5	Aghitu-3 Cave Fauna Layer AH IV	animal remains, paleofauna
Upper Paleolithic - Eurasia	25000	25700	AH IV	GH 6	AH IV assemblage	raw material, technology, typology
-	25000	25700	-	GH 6	Aghitu-3 Cave Fauna Layer AH IV	animal remains, paleofauna
Upper Paleolithic - Eurasia	25000	25700	AH IV	GH 7	AH IV assemblage	raw material, technology, typology
-	25000	25700	-	GH 7	Aghitu-3 Cave Fauna Layer AH IV	animal remains, paleofauna
Upper Paleolithic - Eurasia	25700	27000	AH V	GH 8	AH V assemblage	raw material, technology, typology
-	25700	27000	-	GH 8	Aghitu-3 Cave Fauna Layer AH V	animal remains, paleofauna
-	27000	27500	-	GH 9	Aghitu-3 Cave Fauna Layer AH V	animal remains, paleofauna
Upper Paleolithic - Eurasia	27500	32000	AH VI	GH 10	AH VI assemblage	feature, raw material, technology, typology
-	27500	32000	-	GH 10	Aghitu-3 Cave Fauna Layer AH VI	animal remains, paleofauna
Upper Paleolithic - Eurasia	30000	34500	AH VII	GH 11	AH VII assemblage	raw material, technology, typology
-	30000	34500	-	GH 11	Aghitu-3 Cave Fauna Layer AH VII	animal remains, paleofauna

Human Remains

No data available.

Archeological Finds

Lithics

↓

Assemblage	Function	Technology	Raw material	Lithic piece count	Typology list
AH III assemblage	No	Yes	Yes	4452	**chipped tool**: burin 45, notch 22, perforator 6, scaled piece 7, scraper end 30, scraper side 16, tool backed 174, tool diverse 431, tool truncated 14<br>**non-tool**: core 110, debitage 3597
AH IV assemblage	No	Yes	Yes	5	**chipped tool**: tool backed 1, tool diverse 2<br>**non-tool**: debitage 2
AH V assemblage	No	Yes	Yes	13	**chipped tool**: tool diverse 4<br>**non-tool**: debitage 9
AH VI assemblage	No	Yes	Yes	355	**chipped tool**: burin 1, notch 1, scaled piece 1, scraper end 1, tool backed 5, tool diverse 67<br>**non-tool**: core 3, debitage 276
AH VII assemblage	No	Yes	Yes	155	**chipped tool**: burin 5, perforator 1, tool diverse 8<br>**non-tool**: core 4, debitage 137

Non Lithics

Assemblage	Symbolic artifacts (material: category - interpretation)	Organic tools (material: number - interpretation)	Miscellaneous finds (material: number)	Feature (interpretation)
AH III assemblage	**shell**: ornament - ornament 8	**bone**: 3 - needle eyed 1, pointed artifact 1, unknown 1	**mineral pigment**:	combustion feature
AH VI assemblage	-	-	-	combustion feature

Faunal Remains

Assemblage	Faunal list
Aghitu-3 Cave Fauna Layer AH III	**Bovidae**: Bovinae sp., Capra sp., Caprini sp., Ovis sp.; **Canidae**: Canis lupus, Vulpes vulpes; **Cervidae**: Cervus elaphus; **Cricetidae**: Arvicola amphibius, Arvicolinae sp., Chionomys sp.; **Dipodidae**: Allactaga sp.; **Equidae**: Equus sp.; **Leporidae**: Lepus capensis; **Muridae**: Cricetulus migratorius, Microtus spp.; **Ochotonidae**: Ochotona rufescens; **Suidae**: Sus scrofa;<br>**No family**: Aves indet., Passeriformes indet.
Aghitu-3 Cave Fauna Layer AH IV	**Bovidae**: Capra sp., Caprini sp., Ovis sp.; **Canidae**: Canis lupus; **Cricetidae**: Arvicola amphibius, Arvicolinae sp., Chionomys sp.; **Equidae**: Equus sp.; **Leporidae**: Lepus capensis; **Muridae**: Microtus spp.; **Ochotonidae**: Ochotona rufescens;<br>**No family**: Passeriformes indet.
Aghitu-3 Cave Fauna Layer AH V	**Bovidae**: Bovinae sp., Caprini sp., Ovis sp.; **Canidae**: Canis lupus; **Cricetidae**: Arvicola amphibius, Arvicolinae sp., Chionomys sp.; **Muridae**: Cricetulus migratorius, Microtus spp.; **Ochotonidae**: Ochotona rufescens;<br>**Alaudidae**: Alauda cf. arvensis; **No family**: Aves indet., Passeriformes indet.
Aghitu-3 Cave Fauna Layer AH VI	**Bovidae**: Bovinae sp., Capra sp., Caprini sp., Ovis sp.; **Canidae**: Canis lupus; **Cervidae**: Cervus elaphus; **Cricetidae**: Arvicola amphibius, Arvicolinae sp., Chionomys sp.; **Equidae**: Equus sp.; **Leporidae**: Lepus capensis; **Muridae**: Cricetulus migratorius, Microtus spp.; **Ochotonidae**: Ochotona rufescens; **Soricidae**: soricid sp.;<br>**Alaudidae**: alaudid sp. cf.; **Anatidae**: Anas crecca; **Apodidae**: Apus apus; **Phasianidae**: Alectoris cf.; **Scolopacidae**: scolopacid cf.; **No family**: Aves indet., Galliformes indet., Passeriformes indet.
Aghitu-3 Cave Fauna Layer AH VII	**Bovidae**: Caprini sp., Ovis sp.; **Canidae**: Canis lupus, Vulpes vulpes; **Cricetidae**: Arvicola amphibius, Arvicolinae sp., Chionomys sp.; **Dipodidae**: Allactaga sp.; **Equidae**: Equus sp.; **Leporidae**: Lepus capensis; **Muridae**: Cricetulus migratorius, Ellobius lutescens, Microtus spp.; **Ochotonidae**: Ochotona rufescens;<br>**Accipitridae**: Circus aeruginosus; **Alaudidae**: alaudid sp. cf.; **Anatidae**: Anas cf. platyrhinchos, Anas crecca, Anas platyrhinchos, Aythya cf. fuligula, Aythya sp.; **Corvidae**: Corvus corax; **Fringillidae**: fringillid cf.; **Phasianidae**: Alectoris cf., Alectoris chukar, Alectoris sp., Perdix perdix; **Rallidae**: Porzana porzana; **Scolopacidae**: scolopacid cf.; **No family**: Aves indet., Galliformes indet., Passeriformes indet.

Plant Remains

No data available.

Climate

No data available.

References

Kandel, A.W., Gasparyan, B., Allué, E., Bigga, G., Bruch, A., Cullen, V.L., Frahm, E., Ghukasyan, R., Gruwier, B., Jabbour, F., Miller, C.E., Taller, A., Vardazaryan, V., Vasilyan, D., Weissbrod, L., 2017. The earliest evidence for Upper Paleolithic occupation in the Armenian Highlands at Aghitu-3 Cave. Journal of Human Evolution 110, 37-68.

Kandel, A.W., Gasparyan, B., Nahepetyan, S., Taller, A., Weissbrod, L., 2014. The Upper Paleolithic Settlement of the Armenian Highlands. In: Otte, M., Le Brun-Ricalens, F. (Eds.). Modes de contacts et de déplacements au Paléolithique eurasiatique, Actes du colloque international de la commission 8 (Paléolithique supérieur) de l'UISPP, Université de Liège, 28-31 mai 2012, Presses Universitaires de Liège, Liège, pp. 39-60.

Citation

ROCEEH database (ROAD) 2023. Locality Aghitu-3 Cave. https://www.roceeh.uni-tuebingen.de/roadweb at 02.03.2023

Legend

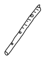 Symbolic artifacts

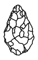 Lithics

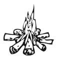 Feature

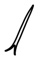 Organic tools

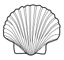 Miscellaneous finds

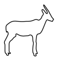 Faunal remains

Appeal

The data contained in this publication represent our interpretation of the published data. While we've taken great care to ensure the accuracy of the data presented in the ROAD Summary Data Sheets, we recognize that they may still contain errors. Therefore we ask for your help in improving the quality of the datasets. Please let us know if you have further information about a site or assemblage, or if you do not agree with our interpretations. We welcome these contributions and will make every effort to update the data. Please e-mail us at road@roceeh.net.
